# Gestational Age Dependence of the Maternal Circulating Long Non-Coding RNA Transcriptome During Normal Pregnancy Highlights Antisense and Pseudogene Transcripts

**DOI:** 10.3389/fgene.2021.760849

**Published:** 2021-11-22

**Authors:** Erica L. Kleinbrink, Nardhy Gomez-Lopez, Donghong Ju, Bogdan Done, Anton-Scott Goustin, Adi L. Tarca, Roberto Romero, Leonard Lipovich

**Affiliations:** ^1^ Center for Molecular Medicine and Genetics, Wayne State University, Detroit, MI, United States; ^2^ Perinatology Research Branch, Division of Obstetrics and Maternal-Fetal Medicine, Division of Intramural Research, Eunice Kennedy Shriver National Institute of Child Health and Human Development, National Institutes of Health, U.S. Department of Health and Human Services, Detroit, MI, United States; ^3^ Department of Obstetrics and Gynecology, Wayne State University School of Medicine, Detroit, MI, United States; ^4^ Department of Biochemistry, Microbiology and Immunology, Wayne State University School of Medicine, Detroit, MI, United States; ^5^ Department of Computer Science, Wayne State University College of Engineering, Detroit, MI, United States; ^6^ Department of Obstetrics and Gynecology, University of Michigan, Ann Arbor, MI, United States; ^7^ Department of Epidemiology and Biostatistics, Michigan State University, East Lansing, MI, United States; ^8^ Detroit Medical Center, Detroit, MI, United States; ^9^ Department of Basic Sciences, College of Medicine, Mohammed Bin Rashid University of Medicine and Health Sciences, Dubai, United Arab Emirates

**Keywords:** fetal development, RNA signature, gene networks, circulating lncRNA, gestational age

## Abstract

In the post-genomic era, our understanding of the molecular regulators of physiologic and pathologic processes in pregnancy is expanding at the whole-genome level. Longitudinal changes in the known protein-coding transcriptome during normal pregnancy, which we recently reported (Gomez-Lopez et al., 2019), have improved our definition of the major operant networks, yet pregnancy-related functions of the non-coding RNA transcriptome remain poorly understood. A key finding of the ENCODE (Encyclopedia of DNA Elements) Consortium, the successor of the Human Genome Project, was that the human genome contains approximately 60,000 genes, the majority of which do not encode proteins. The total transcriptional output of non-protein-coding RNA genes, collectively referred to as the non-coding transcriptome, is comprised mainly of long non-coding RNA (lncRNA) transcripts (Derrien et al., 2012). Although the ncRNA transcriptome eclipses its protein-coding counterpart in abundance, it has until recently lacked a comprehensive, unbiased, genome-scale characterization over the timecourse of normal human pregnancy. Here, we annotated, characterized, and selectively validated the longitudinal changes in the non-coding transcriptome of maternal whole blood during normal pregnancy to term. We identified nine long non-coding RNAs (lncRNAs), including long intergenic non-coding RNAs (lincRNAs) as well as lncRNAs antisense to or otherwise in the immediate vicinity of protein-coding genes, that were differentially expressed with advancing gestation in normal pregnancy: *AL355711*, *BC039551* (expressed mainly in the placenta), *JHDM1D-AS1*, *A2M-AS1*, *MANEA-AS1*, *NR_034004*, *LINC00649, LINC00861*, and *LINC01094*. By cross-referencing our dataset against major public pseudogene catalogs, we also identified six transcribed pseudogenes that were differentially expressed over time during normal pregnancy in maternal blood: *UBBP4*, *FOXO3B*, two *Makorin* (*MKRN*) *pseudogenes* (*MKRN9P and LOC441455*), *PSME2P2*, and *YBX3P1.* We also identified three non-coding RNAs belonging to other classes that were modulated during gestation: the microRNA *MIR4439,* the small nucleolar RNA (snoRNA) *SNORD41*, and the small Cajal-body specific ncRNA *SCARNA2.* The expression profiles of most hits were broadly suggestive of functions in pregnancy. These time-dependent changes of the non-coding transcriptome during normal pregnancy, which may confer specific regulatory impacts on their protein-coding gene targets, will facilitate a deeper molecular understanding of pregnancy and lncRNA-mediated molecular pathways at the maternal-fetal interface and of how these pathways impact maternal and fetal health.

## Introduction

Transcriptomes flux and change dramatically across both space and time throughout all organismal developmental programs, and during the execution of all normal cellular and organismal functions as well as in disease processes. In human pregnancy, dynamic regulation of the transcriptome occurs in the mother, as well within the fetus (Gao et al., 2018). These trends are reflected in the maternal circulation (Gomez-Lopez et al., 2019) and in the amniotic fluid (Tarca et al., 2020) throughout pregnancy. The transcriptome is a critical effector, and reflection, of the successful initial establishment of the prenatal maternal environment, and serves as both a multidimensional set of markers and a corresponding set of biological effectors for normal biological growth, and parturition. We recently found that prenatal cell-free fetal DNA (cfDNA) is associated with inflammation-induced cytokine release response in a murine model of preterm birth (Gomez-Lopez et al., 2020). After conducting a pilot study (Tarca et al., 2019b) to benchmark the Human Transcriptome Arrays (HTA 2.0) for identification of gestational age- and parturition-related expression changes in the coding and non-coding transcriptome, we have subsequently used this platform to profile a larger patient population at six time points throughout pregnancy in order to establish a baseline reference atlas of gestational age modulated gene expression. Furthermore, microbial genomes in fetal plasma (Witt et al., 2020) were recently detected from umbilical cord blood, highlighting the utility of next-generation sequencing to detect intra-amniotic infection and for personalized diagnostics of high-risk complications of preterm birth. Despite these key advances, little remains known about regulation of the non-coding transcriptome during pregnancy, including small (such as micro) and long non-coding RNAs, and the epigenetic as well as post-transcriptional, cis as well as trans regulatory effects of pregnancy-responsive ncRNAs on the proteins and at the key genomic loci that are functionally relevant to pregnancy and/or its complications. Many non-coding RNAs (lncRNAs, miRNAs, expressed pseudogenes, and other classes of non-coding transcripts) have been shown or implicated to play key roles in active regulatory contexts, albeit–especially for most of the still poorly characterized lncRNAs–to an uncertain extent. Nevertheless, it is by now generally appreciated that the non-coding regions of metazoan genomes exert substantial levels of functional control over cellular activities, do so dynamically under various biological conditions, and are hence hypothesized, and likely, to have direct and specific impacts on the transcriptomic and molecular-pathway responses (Dunham et al., 2012), including from early to term normal pregnancy. Although changes with gestational age of protein-coding genes were confirmed by targeted approaches (Tarca et al., 2019b), in this study, we aimed at characterizing using targeted approaches some of the most highly modulated ncRNAs. The roles of these genes will subsequently need to be functionally validated with direct follow-up gain- and/or loss-of-function studies of these ncRNAs in primary cell culture or organoid models of direct relevance to pregnancy.

## Materials and Methods

### RNA Extraction and Microarray Analysis

We previously published the study design (Gomez-Lopez et al., 2019), and here, we utilized the same samples that had been collected in the study cited. In brief, 4–6 blood samples per patient, at various points of the pregnancy timecourse were obtained from 49 patients undergoing normal pregnancy, throughout a longitudinal study conducted at the Center for Advanced Obstetrical Care and Research of the Perinatology Research Branch, NICHD/NIH/DHHS; the Detroit Medical Center; and Wayne State University School of Medicine. RNA was collected in PAXgene^®^ Blood RNA collection tubes (BD Biosciences, San Jose, CA), extracted, quantified and assessed for quality, as previously described (Gomez-Lopez et al., 2019). Although the PAXgene RNA tubes are intended to extract primarily cellular RNA, correlations between such expression changes and those determined by cell-free analysis (Tsang et al., 2017; Ngo et al., 2018) have been reported (Gomez-Lopez et al., 2019; Tarca et al., 2019a). Biotinylated cDNA was prepared and hybridized to Affymetrix ^®^ GeneChip™ Human Transcriptome Arrays 2.0 using the Affymetrix GeneChip™ WT Pico Reagent Kit, stained, and scanned with Affymetrix 3000 7G GeneChipTM Scanner with Autoloader, as previously described (Gomez-Lopez et al., 2019). The Affymetrix AGCC software was utilized to analyze the image files and to obtain raw intensity values for all gene signals present.

### Preprocessing and Expression Calling, and Differential Expression

The microarray data analysis was performed as previously described (Gomez-Lopez et al., 2019). Briefly, the Human Transcriptome Array data were summarized at the level of transcript clusters and tested for association with gestational age using linear mixed-effects models (Gomez-Lopez et al., 2019). In these models, the response variable was the log_2_ transformed gene expression as a continuous variable. Explanatory variables in the models were cubic polynomial terms of gestational age as fixed effects and a random intercept term for each woman, hence accounting for the repeated measures from each pregnancy. Likelihood ratios tests were used to determine if the gestational age terms significantly improved the model fit relative to an intercept only baseline model. Only genes determined to be expressed significantly above background were analyzed.

### Identification and Annotation of Timecourse Differentially Expressed ncRNA Hits

The Affymetrix HTA 2.0 platform includes probesets for over 22 k non-coding and about twice as many coding genes. Even the probesets designed to profile protein-coding gene expression, nevertheless contain probes that map uniquely to lncRNA genes. This is due to persistent misannotations of lncRNAs as mRNAs by automated pipelines during the construction of public databases, progressively increased representation of full-length cDNA clones (many of which are non-coding) from large-scale cDNA sequencing projects, and other complex factors (Orlov et al., 2007). Starting with the list of transcript clusters reported in Supplementary File S1 from our prior study (Gomez-Lopez et al., 2019), we manually verified all putative non-coding RNA gene designations. These include, but are not limited to, gene names starting from any of the following substrings–MIR, LINC, LOC, XR, and NR; named pseudogenes concordant with the Gencode nomenclature (parental gene name ending with number, letter P, pseudogene number); and purely alphanumeric gene names assigned by large-scale sequencing projects without any evidence of protein homology or known function. The latter, including gene names identical to Genbank accession numbers, can in principle, but do not necessarily have to, correspond to previously uncharacterized non-coding RNA loci from high-throughput transcriptome projects.

**FIGURE 1:**
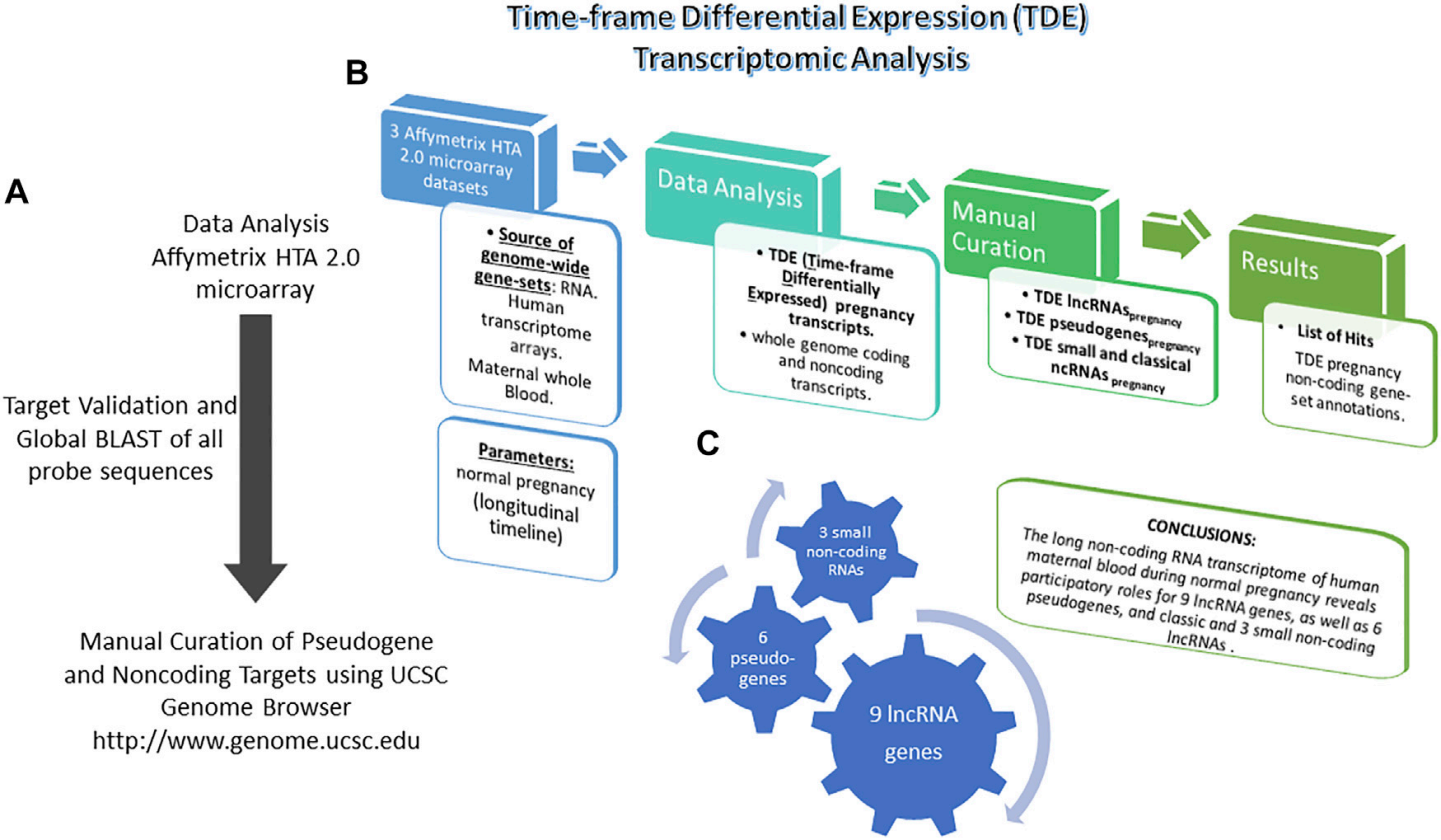
Pregnancy timecourse differential expression analysis, probe curation, filtering and manual annotation of non-coding targets. **(A)** Experimental approach. **(B)** Experimental phases. **(C)** Results of study.

Subsequently, we examined each gene locus concurrently in the Transcriptome Analysis Console software and the UCSC Genome Browser, in order to visually verify the genomic structure similarity between each Affymetrix probeset and its cognate, full-length, public-transcriptome-evidence-supported gene. We thoroughly examined full-length cDNA clones in the “Genbank mRNA” track, and all Expressed Sequence Tags (ESTs) in the “All Human ESTs” track, in order to accurately define the structure of the transcriptional units and to reconcile their strandedness and their exon-intron footprint along the genome with known protein-coding genes, known ncRNA genes (from the RefSeq, UCSC Genes, and Gencode tracks of the UCSC Genome Browser), and Affymetrix probeset mappings. During this process (Figure 1), we further excluded a large number (approximately 90%) of the initial leads, because our visual manual annotation of those loci suggested that the specific indicated transcripts were: protein-coding mRNAs, annotation artifacts, alternative splice variants, extended 5′ or 3′ untranslated regions (UTRs), and/or parts of aberrantly retained introns of known protein-coding genes. Lastly, we excluded transcript clusters with major genomic structure discrepancies relative to the UCSC Genome Browser-displayed transcripts at the loci. All our work utilized the hg19 human genome assembly, because that was the assembly version used for mapping the experimental results of the ENCODE and FANTOM consortia onto the human genome. Supplementary File S5 contains all UCSC Genome Browser supplementary figure illustrations of the corresponding lncRNA gene structures.

### Automated BLAST Searches of the Human Genome with Affymetrix Microarray Individual-Probe Queries

A local BLAST database was built from the primary human genome assembly GRCh37.p13 (GCA_000001405.14). 25 nucleotide long probe sequences from the Affymetrix HTA 2.0 microarray, derived as described above, were aligned against the locally installed human genome assembly using the blastn-short option of blastn, version 2.10.0. The hits with the highest score were counted for each probe sequence (Altschul et al., 1990; McGinnis and Madden 2004). We used the results of these searches to ascertain probe-to-lncRNA assignments and to identify the putative parental genes of transcribed pseudogenes represented by certain probes.

### Quality Control of Affymetrix Probesets

We manually checked for non-redundancy and sequence specificity of the Affymetrix probesets derived above. Also, we manually inspected local BLASTN alignments (the outputs of the searches described above) and concurrently curated them for pseudogenes and lncRNAs, in order to validate, and improve upon, the BLASTN and the UCSC Genome Browser BLAT results. All putative differentially expressed pseudogenes were validated by manual annotation to ascertain locus specificity of the underlying probesets.

### Analysis of Promoterome Data

Each pseudogene and non-coding RNA determined to be significantly changing with gestational age was used to query the FANTOM5 Consortium human hg 19 promoterome (ZENBU) database visually and manually in the FANTOM5 ZENBU Browser, in order to determine the transcriptional orientation, tissue specificity, and expression levels (tags per million, tpm) for each candidate transcribed pseudogene and non-coding RNA (Forrest et al., 2014). This FANTOM5 resource quantifies the entire human promoterome at a single-base resolution, providing one the most comprehensive analysis of oriented, single-end, stranded 5′ CAGE sequencing data available, and covering approximately 1,000 human tissues, and cell types. Genome coordinates on the hg19 human genome assembly, matching each TDE gene locus, were queried in the FANTOM Zenbu genome browser. The cell types and tissues with the highest expression levels (measured in tags per million, tpm) at the manually-determined transcription start site of the TDE transcript, and on the same strand as the transcript, were visualized in the FANTOM CAGE phase 1 and 2 human “pooled, filtered, with 3 or more tags per library” rle (relative log expression) -normalized track.

### Analysis of Regulatory Potential

We analyzed the regulatory potential of TDE lncRNAs in maternal blood circulation during normal pregnancy, using manual annotation of transcription factor binding sites (from the ENCODE Consortium chromatin immunoprecipitation followed by sequencing, i.e., ChIP-seq, tracks in the UCSC Genome Browser), histone modifications (ENCODE ChIP-Seq), and DNAse I hypersensitive sites in the UCSC Genome Browser (GRCh37/hg19). The ENCODE Regulation Track was configured to display (“show”), and the following subtracks were set to “full” display mode: Layered H3K4me1, H3K4Me3, H3K27ac, and DNase clusters. We visually reviewed the evidence for open chromatin (DNAse I hypersensitive sites), promoters (H3K4Me3), enhancers (H3K4Me1, H3K27Ac), and PRC2-repressed sites (H3K27Me3) in the vicinity of all TDE lncRNA and expressed-pseudogene transcription start sites and throughout the gene bodies of all these non-coding transcriptional units.

### Experimental Validation of Selected Timecourse Differentially Expressed (TDE) ncRNA Transcripts Using Quantitative Real-Time PCR

We used total RNA from longitudinal samples collected from women undergoing normal pregnancy. The first-strand cDNA was prepared using SuperScript III First-Strand Synthesis System (Cat# 18080051, ThermoFisher/Life Technologies). The lncRNA expression levels were determined by Taqman quantitative real-time PCR (Taqman qPCR). Taqman primer/probe sets were obtained from ThermoFisher. We used catalog (predesigned) primer/probe sets when available, and custom-designed primer/probe sets for targets lacking predesigned sets (Supplementary File S1). The Ct values were averaged over three RT-qPCR technical replicates per primer/probeset per sample.

Gene expression measurements from Taqman RT-qPCR experiments (−ΔCt = Ct_reference_−Ct_target_) for 6 target lncRNAs were analyzed to assesses the correlation with microarray-based gene expression results in the same patient samples. From the original microarray study (49 patients, 261 samples), a subset of 126 samples were selected to include the first, last and a mid-trimester sample from 42 patients randomly selected from among the 49 in the original study (Supplementary File S2). Since the hypothesis of association between lncRNAs and gestational age was derived from the larger microarray dataset, correlation between microarray and Taqman RT-qPCR data would be considered validation of the lncRNA vs gestational age association. Spearman correlation coefficients and significance *p*-values were obtained using the R statistical language and environment (www.r-project.org). In addition, the association between RT-qPCR gene expression data and gestational age at blood draw was also directly tested using linear mixed effects models to account for longitudinal samples from the same patients (Gomez-Lopez et al., 2019). A two-tailed *p*-value<0.05 was considered a significant result.

## Results

RNA from serial maternal blood samples was profiled using microarrays and expression was quantified at whole-transcript level as well as at the individual-exon level. Of the 614 transcript clusters with expression above background and significant correlation with gestational age at blood draw, 104 were initially assigned to the non-coding category (Gomez-Lopez et al., 2019). The longitudinal profiles of these non-coding RNAs belonged to four broad patterns: Up over time, Down over time, Up then Down, and Down then Up. Among the genes most highly modulated during gestation, there were nine long non-coding RNAs (lncRNAs) and long intergenic non-coding RNAs (lincRNAs) (*AL355711*, *BC039551*, *JHDM1D-AS1*, *A2M-AS1*, *NR_034004*, *LINC00649*, *LINC00861*, *LINC01094*, *MANEA-AS1*) (Table 1) and six transcribed pseudogenes (*UBBP4*, *FOXO3B*, *MKRN9P*, *LOC441455*, *PSME2P2*, *YBX3P1*) (Table 2), as well as three classical non-coding regulatory RNAs (*MIR4439*, *SNORD41*, and *SCARNA2*) (Table 3).

**TABLE 1:** Nine differentially expressed long non-coding RNAs (lncRNAs) and long intergenic non-coding RNAs (lincRNAs). *p*-values and fold changes of time course differentially expressed [TDE] ncRNA transcripts (LincRNAs and antisense lncRNAs) in normal pregnancy. For genes with multiple Affymetrix transcript clusters, only the cluster with the lowest *p*-value is shown.

Gene name	Chromosomal coordinates (Human genome assembly, GRCh37/hg19)	Probeset log_2_FC	Adj. p	*p*
AL355711	chr21:43719104–43720919	−0.64	8.88E-18	1.07E-20
BC039551	chr4:153855668–153857989	0.62	1.03E-10	7.72E-13
JHDM1D-AS1	chr7:139876984–139879440	0.75	8.22E-10	8.17E-12
A2M-AS1	chr12:9217773–9220,651	−0.32	2.09E-08	3.25E-10
NR_034004	chrX:70998019–71004228	−0.38	2.25E-08	3.55E-10
LINC00649	chr21:35303516–35343487	−0.35	1.55E-07	3.21E-09
LINC00861	chr8:126953376–126963441	−0.40	1.70E-07	3.59E-09
LINC01094	chr4:79567148–79605655	0.36	3.11E-05	1.35E-06
MANEA-AS1	chr6:96023059–96025326	−0.34	0.069712732	0.01564686

**TABLE 2:** Six Differentially Expressed Pseudogenes. *p*-values and fold changes of TDE transcribed pseudogenes during normal pregnancy For genes with multiple Affymetrix transcript clusters, only the cluster with the lowest *p*-value is shown. Log2FC is the log2 ratio of the highest and lowest average expression value from 10 to 40 weeks of gestation. The sign is positive if value at 40 weeks is higher than the value at 10 weeks, and negative otherwise.

Gene name	Chromosomal coordinates (Human genome assembly, GRCh37/hg19)	Probeset log_2_FC	Adj. p	*p*
UBBP4	chr17:21729873–21731760	0.38	7.91E-07	2.05E-08
FOXO3B	chr17:18569236–18576494	0.66	2.24E-15	4.27E-18
MKRN9P	chr12:88176663–88178488	0.55	1.44E-11	8.61E-14
LOC441455	chr9:99488103–99489749	0.51	3.21E-11	2.08E-13
PSME2P2	chr13:49345232–49346006	0.32	2.29E-11	1.44E-13
YBX3P1	chr16:31579088–31580845	0.44	7.70E-06	2.72E-07

**TABLE 3:** TDE small and classical non-coding RNAs (ncRNAs) in normal pregnancy. *p*-values and fold changes of timecourse differentially expressed [TDE] ncRNA transcripts are shown.

Gene name	Chromosomal coordinates (Reference genome: Human assembly, GRCh37/hg19)	Probeset log_2_FC	Adj. p	P
MIR4439 (regulatory ncRNA)	chr2:225875178–225875257	0.41	0.002076881	0.00019796
SNORD41 (regulatory ncRNA)	chr19:12817263–12817332	0.41	0.008612927	0.00110102
SCARNA2 (ncRNA)	chr1:109642815–109643234	−0.32	0.004429002	0.00049718

We previously reported that transcripts (predominantly, of protein-coding genes) that are differentially expressed with gestational age in normal pregnancy were enriched in genes located on chromosome 14. The differentially expressed non-coding RNA genes, however, were fairly evenly distributed across the human genome (Tables 1, 2, 3). This might imply more diverse lncRNA etiologies of developmental processes that are not tied to a single chromosome-14 immune response locus.

We used the Functional Annotation of the Mammalian Genome (FANTOM)5 Consortium dataset (Forrest et al., 2014) to interrogate the gestational age-associated (TDE) lncRNAs based on their specificity to 1,000 human organs, tissue types, and primary cell cultures in the FANTOM single-base-resolution promoterome resource, accessed through FANTOM5’s ZENBU interface. The majority of these TDE lncRNAs are robustly expressed in the circulating immune cells, as would be expected, given that these RNAs were isolated from maternal peripheral blood. Supplementary File S5 contains all FANTOM5 ZENBU Browser supplementary figure illustrations of the corresponding lncRNA gene structures.

We found that expression profiles of the TDE lncRNAs and lincRNAs modulated during gestation in normal pregnancy are typically tissue-specific, as is expected of functional lncRNAs, and were broadly consistent with pregnancy-associated functions. This supports the contention that these ncRNAs are relevant to the physiology of GA progression in the maternal-fetal environment, rather than being false positives or expression byproducts of GA-responsive protein-coding loci.

Summarily, expression profiles determined by single-stranded 5′Cap Analysis of Gene Expression (CAGE) RNAseq in approximately 1,000 human tissues and cell types represented in the FANTOM5 ZENBU Promoterome Expression dataset (Forrest et al., 2014) indicate strongly specific expression of TDE lncRNAs in immune cells, in particular lymphocytes and monocytes. This is consistent with direct functional roles of these non-coding RNAs in maternal blood, as opposed to purely being biomarkers of GA with functions in other tissues. As transcriptomic biomarkers (Forrest et al., 2014), these lncRNAs, along with their co-expressed mRNAs, define the transcriptome response to pregnancy over advancing GA (Table 1). Additional functional studies are needed in order to determine conclusively whether these TDE lncRNAs have specific regulatory roles in gestation, fetal growth, and/or at the maternal-fetal interface.

Because of the inherent false positive and false negative findings in genome-scale expression microarray experiments, and because of the genomic landscape complexity as well as the comparatively low expression levels of lncRNA genes, a *de facto* standard emerging in the lncRNA transcriptomics field over the past 2 decades centers on experimental validation of microarray-based lncRNA differential expression results using an independent method in the same samples. Quantitative real-time PCR (RT-qPCR) typically serves as such an independent method. The generally lower expression levels of lncRNAs compared to protein-coding mRNAs make lncRNAs less suitable overall for sybrGreen RT-qPCR. Accordingly, we subjected our top TDE lncRNAs to Taqman RT-qPCR validation, because this method performs better with low-expression targets while the sequence-specificity of the fluorescent Taqman probe guards against false positives. Of the 126 samples selected for RT-qPCR experiments, for 116 there was enough RNA available and analyses were successfully performed.

**FIGURE 2:**
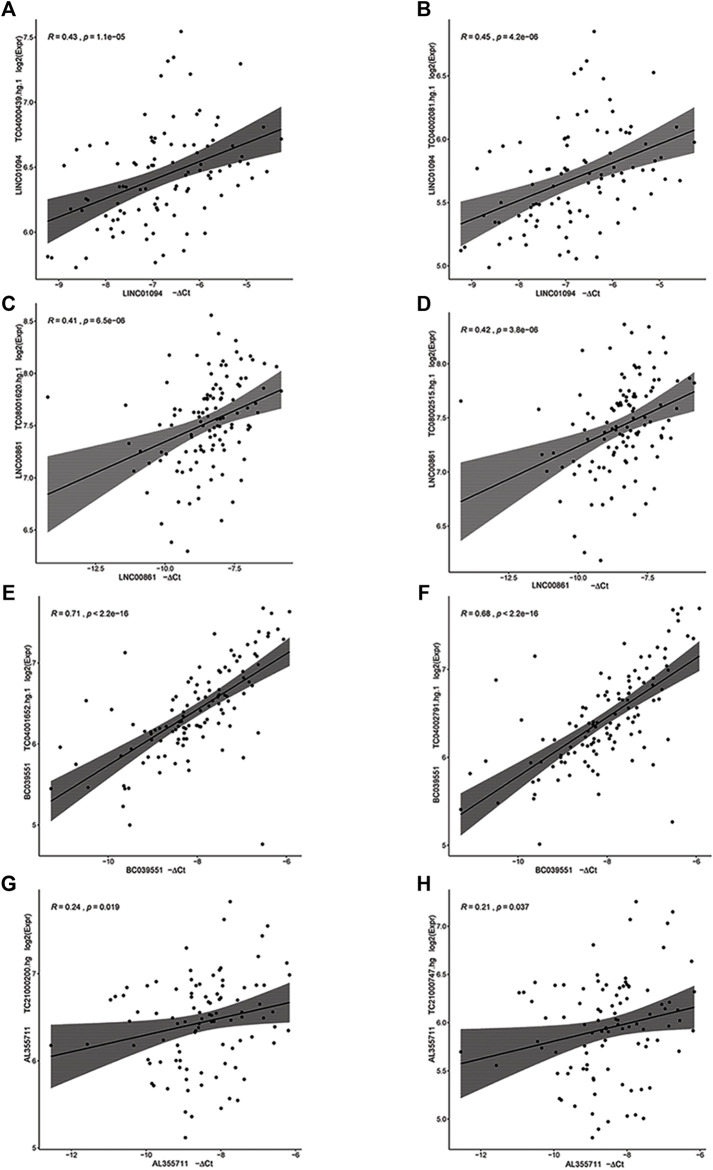
Correlation between microarray (Y-axis) and RT-qPCR (X-axis) log2 gene expression for four lncRNA genes. A linear model fit and 95% confidence intervals of the mean are also shown. **(A,B)** *LINC01094*, **(C,D)** *LINC00861*, **(E,F)** *BC039551*, **(G,H)** *AL355711.*

**FIGURE 3:**
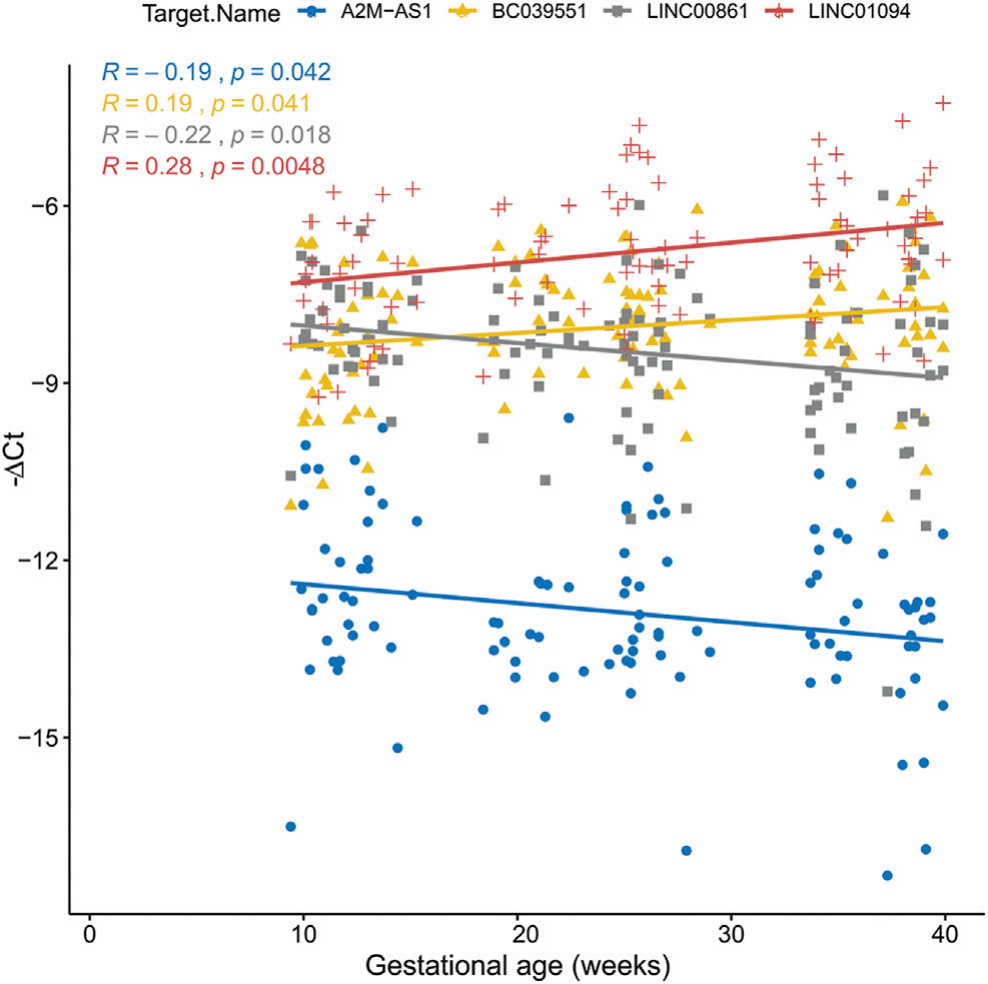
Non-coding RNA changes as a function of gestational age. The figure shows the mean −ΔCt (over three replicate measurements) in each of the 116 samples as a function of gestational age. Lines represent linear mixed-effects models fit of the data.

Of the six lncRNAs tested by RT-qPCR for significant association with gestational age, five were successfully validated (Supplementary Files S3, S4). For four of them (*LINC01094*, *LINC00861*, *BC039551*, and *AL355711*) RT-qPCR expression was significantly correlated with microarray data, based on which the hypothesis was generated (all *p* < 0.05) (Figure 2). For *LINC01094*, *LINC00861*, *BC039551*, and *A2M−AS1*, a direct association with gestational age was also established based on linear mixed effects models in which the gestational age was modeled as a function of gestational age at blood draw and random effects were allowed to account for within patient repeated measurements (*p* < 0.05) (Figure 3).

### Long Non-Coding RNAs and Long Intergenic Non-Coding RNAs Differentially Expressed Over the GA Timecourse of Normal Pregnancy

1) *AL355711*. (*chr21:43719104–43720919*). The single-exon *AL355711* transcriptional unit lies downstream of the known protein-coding gene *ABCG1* and is transcribed in the same direction as the ABCG1 gene. ABCG1 encodes an ATP-binding cassette placental lipid transporter (Körner et al., 2012). Importantly, ABCG1 has a firmly established relevance to diseases of pregnancy: ABCG1 is downregulated in pre-eclampsia, its suppression is thought to contribute to dysregulation of lipid balance at the maternal-fetal interface in pre-eclampsia (Baumann et al., 2013), and downregulation of this transporter is hence likely to impact the health of the growing fetus. However, in contrast to this evidence for a role of ABCG1 in pre-eclampsia, the literature to date lacks any studies of ABCG1 in normal pregnancy. The downstream lncRNA neighbor of *ABCG1*, *AL355711*, is ubiquitously expressed in FANTOM5 CAGE data (Supplementary Figure S1). FANTOM5 RNA expression reads, and the presence of DNase I hypersensitive sites (DHSs) in ENCODE Consortium data for the region, indicate that a potential transcription start site (TSS) exists downstream of *ABCG1*, where *AL355711* resides. The distinct nature of this transcriptional unit is strongly supported by its transcription start site in FANTOM5 CAGE data, which corresponds to an ENCODE DNAse I hypersensitive site. These two features clearly indicate that *AL355711* is not a 3′UTR extension of *ABCG1* but, rather, is a standalone non-coding transcriptional unit. The transcript may have functional roles that are independent of *ABCG1*, but given the evidence for *ABCG1* roles in pre-eclampsia, an alternative hypothesis is that *AL355711* may interact with, and/or regulate, *ABCG1* through cis-regulatory mechanisms. One possibility is that *AL355711* may act as an enhancer RNA (eRNA); eRNAs are lncRNAs transcribed off enhancers (lncRNA promoters) that regulate the expression of nearby, and sometimes distant, protein-coding genes.
2) *BC039551.* (*chr4:153855668–153857989*)*.* Intriguingly, the highest expression of this lncRNA in the FANTOM5 catalog of 1,000 human tissues, organs, and cell types was observed in the placenta (Supplementary Figure S2)—suggesting that a function in pregnancy is highly likely, in view of the fact that expression of this lncRNA rises, slowly but significantly, over GA. This lncRNA is antisense (Wood et al., 2013) to, and/or (depending on the splice isoform used for annotation) shares a bidirectional promoter with, the adjacent protein-coding gene *FHDC1*, which encodes an inverted formin with actin-dependent functions in ciliogenesis that does not currently have any known pregnancy-associated phenotypes.
3) *JHDM1D-AS1*. (*chr7:139876984–139879440*). This lncRNA is antisense to the protein coding gene, *JHDM1D*, which encodes a Jumonji-domain-containing histone demethylase required for brain development. *JDHM1D* is, just like *ABCG1*, associated with pre-eclampsia (Luo, 2018), but its functions during normal pregnancy have not been elucidated. We have recently identified expression of this lncRNA in amniotic fluid as well as in amniocytes cultured from fetal membranes (A.-S. Goustin, manuscript in preparation), pointing to a wider spectrum of possible functions in pregnancy. In FANTOM5 data (Supplementary Figure S3), expression of this lncRNA is detected primarily in whole blood as well as in specific types of immune cells (e.g., monocytes, neutrophils). This expression profile is potentially reflective of the broad interplay between immunity and pregnancy.
4) *A2M-AS1*. (*chr12:9217773–9220651*)*.* This lncRNA is antisense to the alpha 2 macroglobulin gene. *A2M-AS1* is expressed in monocyte-derived dendritic cells in Heliscope CAGE data in the FANTOM5 Human hg 19 promoterome database (Supp Fig. 4), while its overlapping protein-coding gene transcribed in the opposite direction, *A2M* (hg19 chr12:9,220,304-9,268,558), also has relatively strong expression in monocyte-derived dendritic cells (sense signal: 0.79 rle, antisense signal: 23.9 rle). This expression profile is broadly concordant with the well-characterized connection between immunity and pregnancy, including immune tolerance of the semi-allograph fetus; circulating CD14+ monocytes are known to be activated in the complications of pregnancy (Naccasha et al., 2001), including preterm labor (Gervasi et al., 2001).
5) *NR_034004* ***.*** (*chrX:70998019–71004228*)*.* This lincRNA lacks appreciable expression in any FANTOM5-represented tissues or cell lines (Supp Fig. 5). In this study, it was for the first time detected in maternal blood circulation, where its expression steadily and significantly decreased with advancing gestation. In the absence of additional expression data and nearby or overlapping genes, there is no known evidence to pinpoint a specific function. However, one of the goals of this work, was to use the pregnancy transcriptome to discover novel potential functional contributors to, and predictors of gestational age. The expression profile of this previously wholly-uncharacterized lincRNA places it in that category. The biological reasons behind its significant TDE in pregnancy warrant further study.
6) *LINC00649*. (*chr21:35303516–35343487*). Similarly to some of the above lncRNAs, this lincRNA is expressed in immune cell types: CD14+ monocytes, CD4+ and CD8+ T-cells, and NK cells (Supp Fig. 6). Our UCSC Genome Browser-based visualization of this locus showed that some transcript isoforms of *LINC00649* share a bidirectional promoter with, while other isoforms of *LINC00649* are transcribed antisense to, the *ATP5O* gene, which is expressed in the placenta and has been used as an expression control in human placental tissue (Hůlková and Zeman 2011). Since lncRNAs sharing bidirectional promoters with protein-coding genes are frequently co-expressed with those genes (Engström et al., 2006), while antisense lncRNAs of protein-coding genes often serve as positive, and sometimes as negative, regulators of their overlapping protein-coding genes (Katayama et al., 2005) possible placenta-associated functions of *LINC00649* should be investigated.
7) *LINC00861*. (*chr8:126953376–126963441*)*.* This lincRNA is expressed mainly in T cells, NK cells, and other immune cell types (Supp Fig. 7, Supp Fig. 29).
8) *LINC01094.* (*chr4:79567148–79605655*)*.* This lincRNA’s expression profile includes the brain, spinal cord, and monocytes (Supp Fig. 8)
9) *MANEA-AS1.* (*chr6:96023059–96025326*)*. MANEA-AS1 is* expressed at a low level in thymus and lymphocytes. This antisense lncRNA is also detectable in various cancers, as well as in normal liver and brain (Supp Fig 9, Supp Fig 23). It is expressed in several reproductive tissues, as well as in the arteries and bladder. There is no known pregnancy function for its sense overlapping gene MANEA, which encodes a mannosidase. Summarily, this antisense lncRNA, with a small but significant decrease of expression level over the gestational age span, belongs to the category lncRNAs not previously associated with physiologic processes in pregnancy, that have, for the first time, been identified as modulated in maternal circulation with advancing gestation.

One common thread connecting all 10 of these TDE lncRNAs is their expression in tissues, cell types, and conditions that are directly relevant to pregnancy and its complications: placenta, amniocytes, various immune cell types including CD14+ monocytes; and multiple direct associations with pre-eclampsia. This strongly supports our contention that these TDE lncRNAs circulating in maternal blood are not mere biomarkers of GA, but are blood-expressed lncRNAs that have functional roles in pregnancy in other tissues–which presumably explains their differential expression in maternal blood detected herein.

### Seven Transcribed Pseudogenes Differentially Expressed Over the GA Timecourse of Normal Pregnancy

Pseudogenes are incomplete and/or mutated copies of protein-coding genes that have limited or absent protein-coding capacity due to their partial length and/or the sequence substitutions that distinguish them from the functional protein-coding genes which gave rise to them (also called “parental genes”). Pseudogenes are broadly divided into two types: unprocessed pseudogenes, which arise through duplications of large parts of genomic sequence followed by divergence of the pseudogene according to the molecular clock at the neutral substitution rate, and processed pseudogenes, which are the outcome of endogenous reverse transcription (by reverse transcriptases encoded in ERV repeats in the genome or as a consequence of retroviral infection) of cellular mRNAs into intronless cDNA copies that become genomically integrated. Pseudogenes of both types, while previously considered to be dysfunctional genomic remnants of protein-coding gene copies, are frequently transcribed into RNA, and transcribed pseudogenes have been recently shown to regulate their cognate parental genes in a sequence-specific fashion and to engage in multiple epigenetic and post-transcriptional gene regulation mechanisms (Milligan et al., 2016). Several recent reports demonstrate the functional contributions of pseudogenes to disorders of pregnancy, including pre-eclampsia (Lv et al., 2018; Tong et al., 2018; Li and Li 2020). However, the normal pregnancy expression profile of transcribed pseudogenes has not been globally interrogated prior to our study.

1) *UBBP4.* (*chr17:21729873–21731760*)*.* This transcribed pseudogene is an expressed non-retropseudogene whose parental gene is also differentially expressed in normal pregnancy with GA in our previously published mRNA dataset. UBB, its parental gene, was identified through network analysis as 1 of 5 Hub genes associated with pre-eclampsia (Jiang et al., 2015) (Supp Fig. 10, Supp Fig 24). Considering the evidence that expressed pseudogenes may regulate their cognate parental genes (Milligan et al., 2016) the potential significance of UBBP4 as a modulator of pre-eclampsia deserves further study.
2) *FOXO3B*. (*chr17:18569236–18576494*)*. FOXO3B* is a transcribed retropseudogene, located sense to and inside an intron of a *ZNF*, i.e., zinc finger protein, gene. This pseudogene is expressed in the uterus, and is supported by a full-length mRNA (cDNA clone) from a leiomyosarcoma (cDNA). Its parental gene is also TDE in normal human pregnancy, and is expressed in numerous reproductive tissues in GTEx RNAseq data in the UCSC Genome Browser: ovary, fibroblasts, endocervix, uterus, ectocervix, fallopian tubes, vagina, and mammary tissue (Supp Fig. 11, Sup Fig 25). The parental gene *FOXO* is mentioned in more than 30 studies that highlight its relationship to pregnancy.
3) *MKRN9P*. (chr12:88176663-88178488). *MKRN9P* (Makorin pseudogene 9) is expressed mostly in the lung and cancers at low levels in FANTOM5 CAGE data. The parental gene, encoding Makorin, is also TDE in our GA normal pregnancy timecourse dataset (Supp Fig 12, Supp Fig 26).
4) *LOC441455*. (*chr9:99488103–99489749*). This lncRNA is expressed in numerous tissues in GTEx and represents the transcript of another makorin ring finger protein 1 pseudogene.
5) *PSME2P2*. (*chr13:49345232–4934600*) is a retropseudogene expressed in an ovary tumor (according to EST data in the UCSC Genome Browser), while its parental gene is also TDE in our dataset (Supp Fig 13, Fig 22). GTEx RNA-seq of 8555 samples shows expression of this pseudogene in the spleen (8.3 TPM, transcripts per million), adrenal gland (6.7 TPM), pituitary (6.4 TPM), cervix- endocervix (6.1 TPM), small intestine- terminal ileum (6.1 TPM), lung (5.4 TPM), thyroid (5.4 TPM), and uterus (4.9 TPM).
6) *YBX3P1*. (*chr16:31579088–31580845*). Y-box binding protein 3 pseudogene 1 (a transcribed putative retropseudogene), also known as CSDAP1, is predominantly expressed in testis and skeletal muscle in GTEx (Supp Fig. 14). Interestingly, the parental gene is TDE in our normal pregnancy GA timecourse, as is this pseudogene*.*
7) *LINC00861.* Long intergenic non-protein coding RNA 861 (chr8:126953376*–*126963441) is expressed in most blood components in FANTOM 5′CAGE an also in whole blood and spleen by GTEx (Supp Fig. 7 and Supp Fig. 29)*.* Summarily, at least 2 of these 7 TDE transcribed pseudogenes are clearly associated with pregnancy and pre-eclampsia through the known functions of their parental genes. For the additional TDE transcribed pseudogenes a circumstantial association with pregnancy functions via expression in immune cells is plausible given their expression profiles, which indicate transcription in the uterus, cervix, and other female reproductive organs and tissues.

### Three Other Non-Coding ncRNAs Differentially Expressed with GA in Normal Pregnancy

Expression levels of microRNA-4439 (*MIR4439*) (chr2:225875178-225875257) are shown in (Supp Fig. 15) in the FANTOM5 Consortium ZENBU Browser view of human tissues and cell types with the highest expression of MIR4439 ranked at the top, as are the profiles for *SNORD41* (a C/D-box small nucleolar RNA which belongs to a class of regulatory RNAs responsible for ribosomal RNA precursor editing) (chr19:12817263-12817332) (Supp Fig. 16) (Schulten et al., 2017), and *SCARNA2* (Supp Fig. 17), (*Homo sapiens* small Cajal body-specific RNA 2, chr1:109642815-109643234) (Gérard et al., 2010).

## Discussion

Recent advances in sequencing and global unbiased analyses of tissue and timecourse transcriptomic signatures can help reveal the functions of lncRNAs in gene networks, including those in the cell and tissue types most pertinent to pregnancy and maternal-fetal medicine. The high tissue-specificity of TDE lncRNAs in pregnancy would make them potentially attractive therapeutic targets, if the deregulation of any specific TDE lncRNAs were to be discovered during disorders or complications of pregnancy. Expression profiling of these TDE lncRNAs, which we have now characterized in the normal pregnancy GA timecourse, should be also studied in pregnancy complications.

A search of Pubmed-indexed publications for the co-occurrence of terms “gestational age” and “lncRNA” results in a list of 49 publications, as of the time of this writing. However, to date, only four of those publications have investigated the transcriptome (Finn et al., 2014; Gormley et al., 2017; Hussey et al., 2020; Wang et al., 2020), instead of focusing on specific-gene case studies. Specific components of the human lncRNA transcriptome in gestation have recently been elucidated. RNA sequencing to compare normal pregnancy with fetal growth restriction (FGR) (Wang et al., 2020) pinpointed *RP11-552M6.1*, *LINC01291*, and *ASGR1* (umbilical cord blood) and *SFRP2*, *miR-432-5p*, and *miR-1306-3p* (maternal peripheral blood). Of these non-coding targets functionally associated with and active in FGR (Wang et al., 2020) one was, a Gencode lncRNA originally found, pertinently, in fetal liver in 1999 (Genbank accession number AF090889, found in our UCSC Genome Browser-assisted manual annotation of this locus). *RP11-552M6.1* is antisense to the protein-coding gene *Kruppel-like factor 12* (*KLF12*). *KLF12* is ubiquitously expressed in numerous organs and tissues, including placenta and uterus (RNA-Seq Data from GTEx, data not shown) and few reported roles in pregnancy. *KLF12* is a significant quantitative trait locus from multiple GWAS (Genome-Wide Association Studies) for a host of non-pregnancy associated outcomes such as cancers (Petersen et al., 2010), lipids (Kathiresan et al., 2007), eye disorders (Macgregor et al., 2010), sudden cardiac arrest (Aouizerat et al., 2011), and heart function abnormalities (Sotoodehnia et al., 2010). GWAS is a highly reliable, robust, and well-established method that utilizes population genetics and genomic epidemiology to identify new contributing candidate genes, in an unbiased manner, for common diseases that have a genetic component; these genes can be functionally validated in the laboratory for development into biomarkers or drug targets (Manning et al., 2020). The antisense intronic lncRNA of the *KLF12* gene, *RP11_552M6.1*, is a promising functional target, because *KLF12—*despite its promiscuous expression*—*is known to repress *AP-2*, the *alpha*-a developmental specific transcription factor. We can envision a role for this locus in FGR of pregnancy that involves tissue- and developmental stage-specific antisense-driven and regulation of the ubiquitously expressed *KLF12* gene by the lncRNA. Similarly, we might posit a role for *MIR1306* and *miR-432-5p* in post-transcriptional processing affecting mRNA stability in abnormal pregnancy outcomes, especially for *MIR1306,* because it is highly expressed in reproductive organs, including the uterus and vagina (data not shown, GTEx).

Despite the growing number of studies of lncRNA in the disorders of pregnancy, the circulating lncRNAome of normal pregnancy over gestational age in humans has been comparatively little investigated. Our study is the first to analyze the lncRNAome over time during normal pregnancy. In addition to GA-dependent lncRNAs in maternal circulation, our analysis, has uncovered other regulatory ncRNAs that were differentially expressed with GA in normal pregnancy in maternal blood: *MIR4439* (*log*_*2*_ *FC =0.41*, *adj.p= 0.0021*, *p= 0.0002*), which was originally discovered in human B-cells (Jima et al., 2010), *SNORD41* (*log*_*2*_ *FC =0.41*, *adj. p= 0.0099*, *p= 0.0011*), and *SCARNA2* (*log*_*2*_ *FC* = −0.32, adj. *p* = 0.0044, *p* = 0.0005). GA was associated with opposite trends in expression levels of the 3 DE regulatory ncRNAs, suggesting selective effects on the network effectors specific for each regulatory ncRNA throughout the timeframe of gestation. For instance, *MIR4439* expression dropped in expression roughly mid-gestation and slowly rose again over time until birth. On the other hand, *SNORD41* rose subtly but significantly over time, while the lincRNA *SCARNA2* (non-coding RNA) dropped rather dramatically over the entire course of pregnancy.

Several of TDE lncRNA genes are robustly expressed in circulating CD14+ monocytes, a cell type strongly functionally associated with preterm labor (Gervasi et al., 2001; Pique-Regi et al., 2019), and other pregnancy complications (Gervasi et al., 2001; Gervasi et al., 2002; Oggé et al., 2010). *LINC00649* (chr21:35303516-35343487) (*log*_*2*_ *FC*= −0.35, adj. *p* = 1.55E-07, *p* = 3.21E-09) is expressed in immune cell types (as are several other TDE lncRNAs we found), including CD14+ monocytes, CD4+ and CD8+ T-cells, and NK cells. No apparent literature links *LINC00649* to any aspects of pregnancy. *LINC00649* was shown to be an adverse prognostic marker in AML and is a putative part of a competing endogenous RNA (ceRNA) network (Guo et al., 2020) that includes key AML regulators.

These preliminary results warrant a comprehensive *in-silico* analysis and experimental validation of miRNA targets, the ncRNA-protein interactions, and their corresponding gene networks associated with pregnancy outcomes. The limited information currently available on *LINC00649* complicates predictive analyses (since we do not know which networks encoded at and regulated by this locus are actually utilized during pregnancy). *LINC00649* directly harbors a GWAS SNP, rs35184820, significantly associated with menarche (age at onset) in 242,000 European-ancestry individuals (Kichaev et al., 2019). Other GWAS SNPs reside within and near this lncRNA–but not at *ATP5O*, which alludes to direct functional significance of the lncRNA itself rather than a bystander effect from *ATP5O*–are reported for hematocrit, erythrocyte counts, glomerular filtration rate, and FEV/FEC ratio. *LINC00649* shares a bidirectional promoter with, and (depending on the splice isoform and on the transcription start site used) is antisense to, *ATP5O*, which encodes an F-type ATPase mitochondrial matrix protein associated with type II diabetes (T2D) in the context of its skeletal muscle mRNA expression profile (Rönn et al., 2009). This gene is also reported among the top 20 genes upregulated in benign epithelial ovarian cysts compared to normal ovarian tissue [see Table 2; (Liu et al., 2017)] but no prior work investigated its role over GA during pregnancy in humans.

*LINC00861* (*log*_*2*_ *FC* = −0.40, adj. *p* = 1.70E-07) is another lincRNA differentially regulated over GA that displays a strong immune cell expression profile, as does *LINC00649*, including expression in T cells, NK cells, and other immune cell types, according to RNAseq data from the GTEx Portal multiple tissue expression database. Thus, the detection of this lincRNA in maternal blood is expected, and the lincRNA might be relevant to the complications of pregnancy. Intriguingly, the region immediately adjoining this lincRNA transcriptional unit harbors the GWAS SNP rs16901004, significantly associated with endometriosis in 171 European-ancestry cases and 2,934 European-ancestry controls (Sobalska-Kwapis et al., 2017). Previous reports have implicated this lincRNA in survival in ovarian cancer (Zheng et al., 2020), recurrence of hepatocellular carcinomas (Lv et al., 2018), as a clinical marker in breast cancer (Zhang et al., 2017), and in non-smoking COPD (Qian et al., 2018). A role in gestational-age dependent physiology is slightly perplexing in light of the lincRNA’s strong predilection for associating with cancer-related phenotypes and as a biomarker in oncological clinical disease (Zhang et al., 2017; Lv et al., 2018; Zheng et al., 2020). In view of the classical “cancer/stem cell” similarity of the oncotranscriptome with the transcriptome of pluripotent cells and broadly of development, one possibility is a role for the lincRNA in growth and matrix stability in cell and various tissues, but not as a marker for actual carcinogenic and transformative process *per se*. Additional studies are necessary to clarify the functional basis of our characterization of this lincRNA as a maternal circulating transcriptome biomarker of GA.

The forkhead box FO3B pseudogene (*FOXO3B)* (*log*_*2*_ *FC* ***=*** 0.66, adj. *p* = 2.24E-15) is a GA-associated transcribed retropseudogene whose genomic context and parental-gene functions are suggestive of a role in pregnancy. *FOXO3B* is highly expressed in whole blood (like many of our other TDE, GA-responsive lncRNAs and pseudogene transcripts) and in neutrophils (Supp Fig. 11, 25). The FOXO transcription factor genes (*FOXO*s), which include this pseudogene’s parental gene, are highly associated with pregnancy and its adverse outcomes in humans [for a review see Brosens et al. (2009)], while in mice the *FOXO*s are required for growth of the preimplantation embryo as a consequence of their involvement in apoptotic processes and cell cycle arrest (Kuscu et al., 2019). The balance of expression levels between the 1 and 3a *FOXO* transcription factors is protective against oxidative damage during endometrial decidualization (Kajihara et al., 2006). These pivotal roles for *FOXOs* are expected, given that the conserved “forkhead” family of transcription factors [those containing a forkhead box (FOX) DNA binding domain] includes the FOXOs, and their deregulation originally empowered the discovery of the *Drosophila melanogaster* mutant phenotype (*fkh*) that subsequently led to one of the first identifications of an important developmental family of transcription factors [for a review see Hannenhalli and Kaestner (2009)]. The parental gene is DE with GA as well. The FOXO family of transcription factors have long been known as regulators of the cellular changes that accompany maternal tissue growth and remodeling throughout the gestation process (Kajihara et al., 2006; Brosens et al., 2009). Based on its expression pattern, the *FOXO3B* pseudogene is one of the most striking examples of the DE genes with GA in this study, being present in many maternal reproductive tissues according to GTEx expression profiles- including ovary, fibroblasts, endocervix, uterus, ectocervix, fallopian tubes, EBV-transformed lymphocytes, vagina, testes and mammary tissue- a highly specific expression profile consistent with a function in gestation. We are the first to report that this pseudogene is also differentially expressed with GA during pregnancy. The combination of this pseudogene’s TDE status in pregnancy and the prominence of the female reproductive system for the expression profile strongly alludes to a yet-to-be-characterized function in pregnancy for this transcribed pseudogene.

In addition to the *FOXO3B* pseudogene, we identified several GA-responsive (TDE) pseudogenes expressed in maternal circulation during normal pregnancy: *UBBP4*, *MKRN9P*, *LOC441455* (also a makorin pseudogene), *PSME2P2*, and *YBX3P1*. The past decade has seen the emergence of multiple experimentally validated modalities of pseudogene-dependent gene regulation, including networks where pseudogene transcripts function as ceRNAs, which are typically non-coding or pseudogene transcripts that compete with their sequence-cognate mRNAs for binding miRNAs and regulatory factors (Salmena et al., 2011; Bosson et al., 2014; Qu et al., 2015), an initially speculative hypothesis which has garnered progressively more extensive support (Vitiello et al., 2020). The net effect of ceRNA networks is, typically, a sequestration of free miRNAs, through miRNA direct binding to cognate binding sites localized in pseudogene transcripts instead of the 3′UTRs of the cognate mRNAs, thereby de-inhibiting the original miRNA-driven post-transcriptional downregulation of those particular mRNAs. The transcription of the corresponding lncRNA or pseudogene is a prerequisite for this process. That we report a large number of TDE, GA-responsive genes in normal pregnancy which fall into this category highlights the heretofore-unrecognized, and potentially extensive, functions of pseudogene-mediated transcriptional networks in the processes of gestation and parturition, and potentially in pregnancy complications in both the mother and fetus.

The lncRNA *JHDM1D-AS1*, another TDE GA-responsive hit in our analysis, is antisense to the well-characterized protein-coding gene *JHDM1D*, which encodes a histone demethylase that demethylates H3K9me2 and H3K27me2, and that binds the classic active-promoter, transcription start site histone modification H3K4me3 (Horton et al., 2010; Qi et al., 2010; Tsukada et al., 2010). Reduced expression levels of *JHDM1D* in the placenta accompany pre-eclampsia (Luo et al., 2018), but a function for this locus during normal pregnancy, if any, remains unknown. RNAi with si*JHDM1D*, to mimic the reduction in expression levels seen with pre-eclampsia, induced apoptosis in the HTR-8/SVneo trophoblast cell line (Luo et al., 2018), and may increase methylation at HLA-G where JHDM1D binds (Tang et al., 2015; Luo et al., 2018), resulting in a concomitant drop in *HLA-G* as well, hypothetically also during pre-eclampsia. This pattern correlates with the reduced expression of HLA-G proteins, an immune change found commonly in maternal serum and placental tissues during pre-eclampsia (Yie et al., 2004). We have identified the expression of this antisense lncRNA in amniotic fluid, and we have RT-qPCR-validated the expression of this lncRNA in amniocytes isolated from cultured membranes (A.-S. Goustin, manuscript in preparation), indirectly supporting a likely specific role for this antisense transcript in pregnancy. Here, we report for the first time that *JHDM1D-AS1* is dynamically regulated over time during gestation in maternal peripheral blood, slowly rising over increasing GA and slightly prior to birth (*log*_*2*_ *FC = 0.63*, *adj. p = 1.62E-09*).

LncRNAs are, as a rule, globally expressed at lower levels than are mRNAs of protein-coding genes, while their tissue- and cell-type-specificity equal or exceed those of protein-coding genes and can be used as windows into function (Derrien et al., 2012). In agreement with those fundamental early findings of the ENCODE Consortium, here we found that, while expression profiles of TDE lncRNAs and lincRNAs in FANTOM5 Consortium datasets were low for some though not all of the genes, their expression was overall highly tissue- and organ-specific, often in patterns strongly suggestive of a functional rather than purely a blood-biomarker association with pregnancy. The tissue-specificity of some of the GA responders, especially those with relatively high expression levels in one or relatively few tissues, indicates that—if their functions can be proven—they and their downstream targets may prove potentially attractive for therapeutics. Knowing the expected trajectory of gene expression during normal pregnancy will empower disease diagnostics through personalized transcriptomics; RNAseq of individual-patent samples is affordable, will pinpoint significant deviations from the baseline that we have characterized here, and will define the directions of the desired therapeutic adjustments (up or down, and specific GA) for each gene. Summarily, here we have provided the first ever atlas of lncRNA, lincRNA, antisense lncRNA, and transcribed-pseudogene differential expression in maternal peripheral blood over the timecourse of normal human pregnancy, advancing the field beyond mere transcriptomic biomarkers and toward the discovery of direct pregnancy roles of the lncRNAome. Generating testable predictions is easier for antisense lncRNAs and expressed pseudogenes than for true intergenic lincRNAs, thanks to the known roles of their overlapping sense protein-coding genes and parental protein coding-genes respectively. Therefore, these categories of lncRNAs serve as a valuable starting point in adjusting our focus from a Universe of circulating biomarkers to a constellation of new therapeutic targets for the prevention of pregnancy complications and adverse neonatal outcomes (Huang et al., 2018).

## Data Availability

The original contributions presented in the study are included in the article/Supplementary Material, further inquiries can be directed to the corresponding authors.
