# Supplementary figures and images for: Gestational Age Dependence of the Maternal Circulating Long Non-Coding RNA Transcriptome During Normal Pregnancy Highlights Antisense and Pseudogene Transcripts
Source: Front Genet. 2021 Nov 22;12:760849. doi: 10.3389/fgene.2021.760849 (PMC8645989; doi:10.3389/fgene.2021.760849)

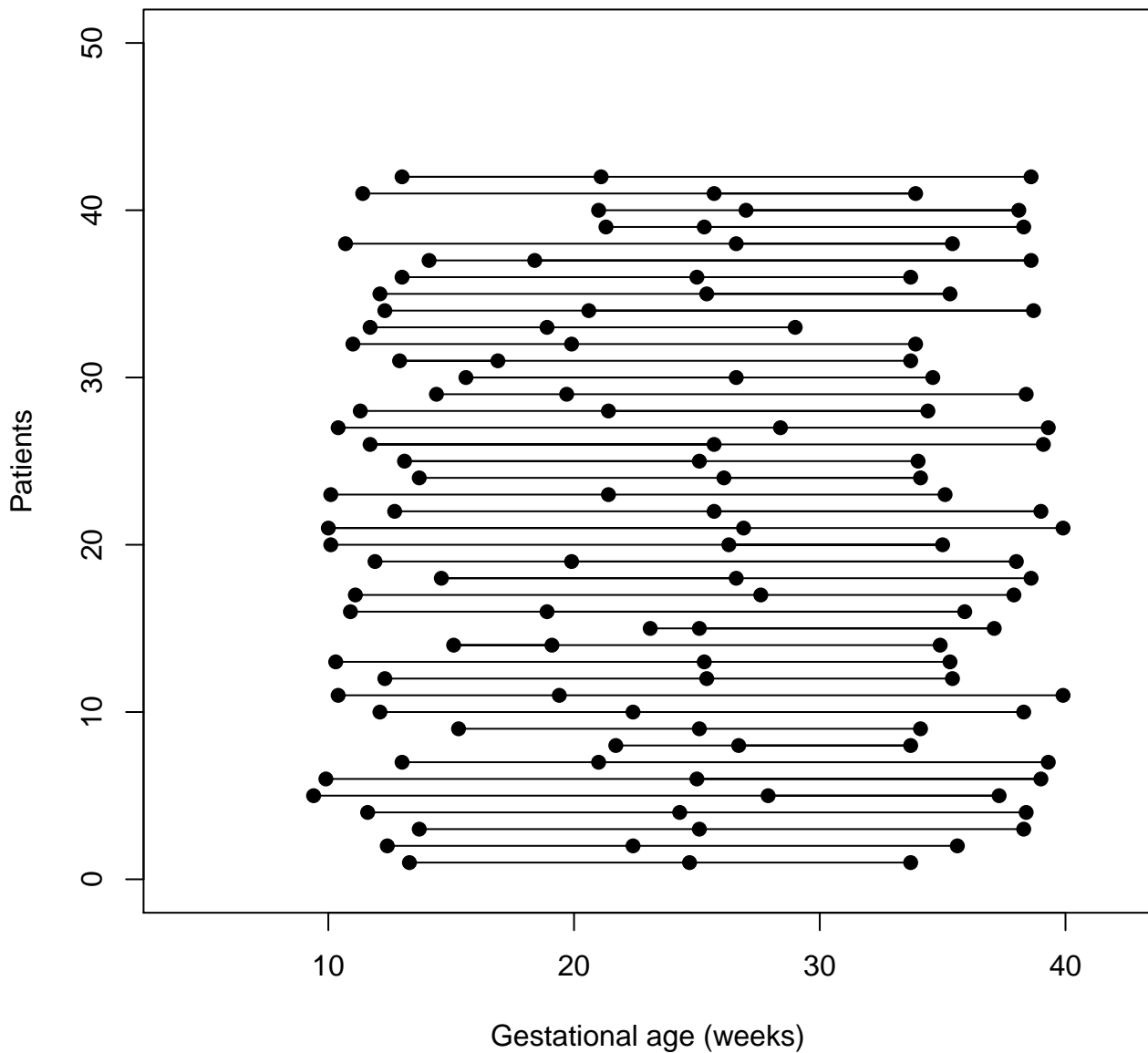

Supplement: Supplementary file 1 [file DataSheet2.PDF]

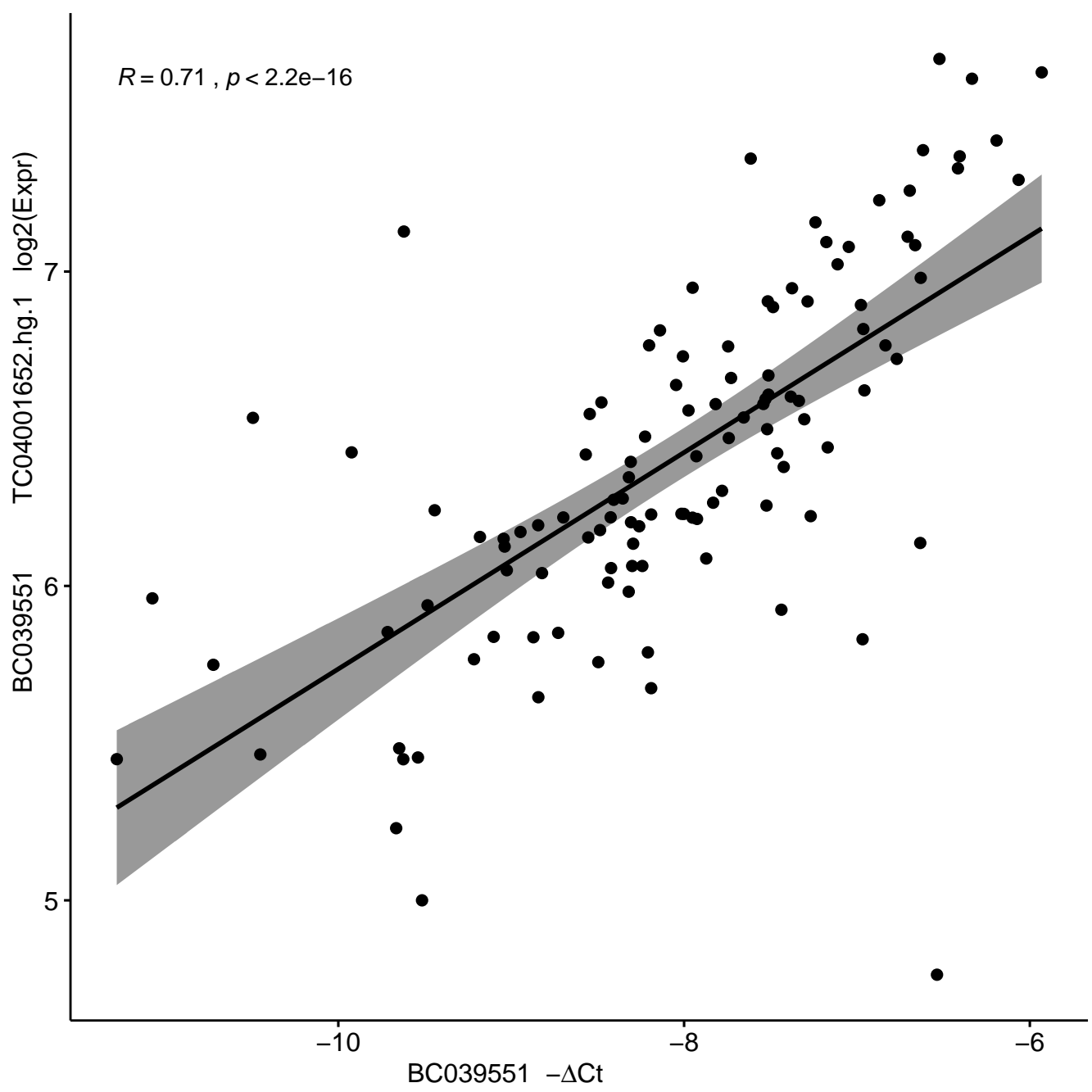

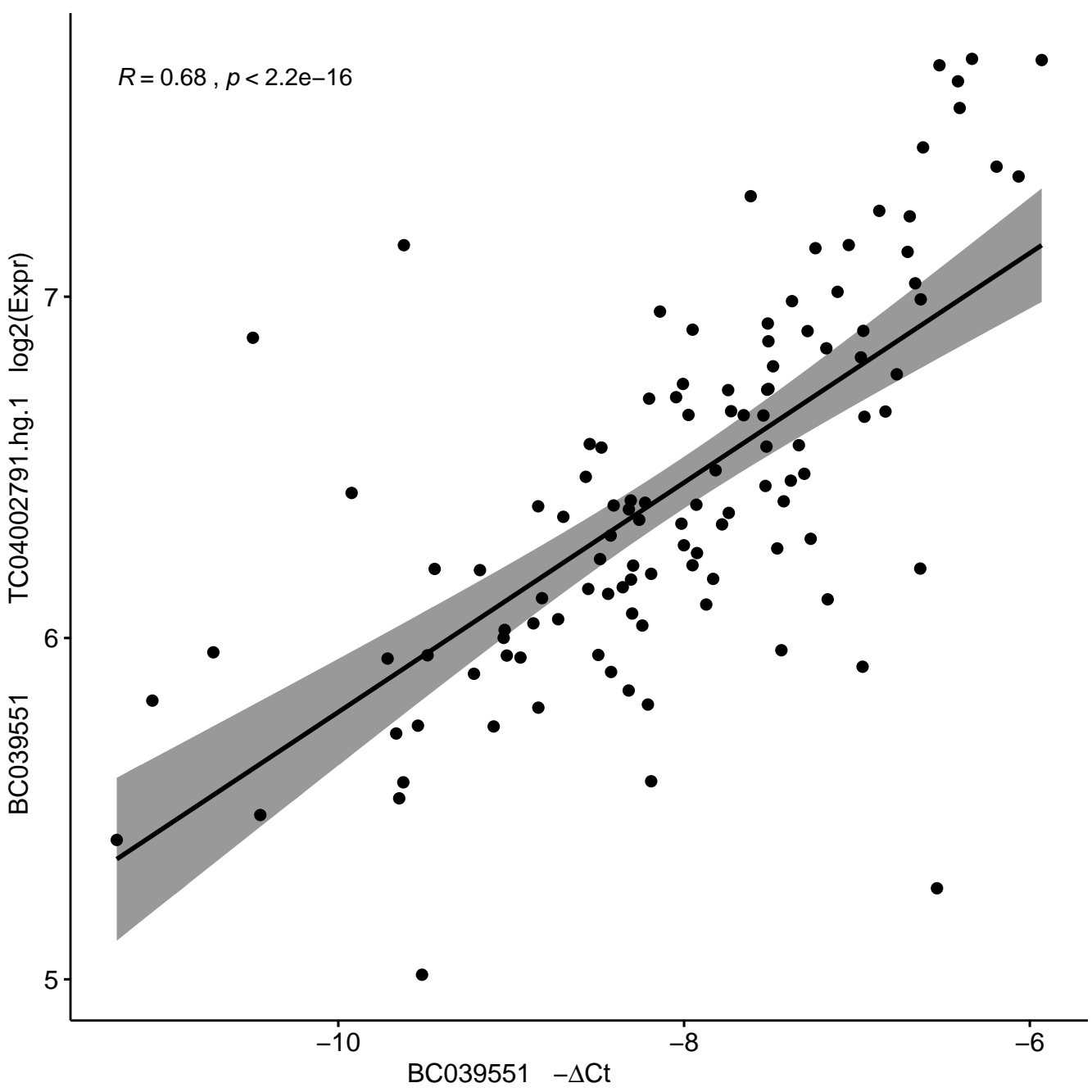

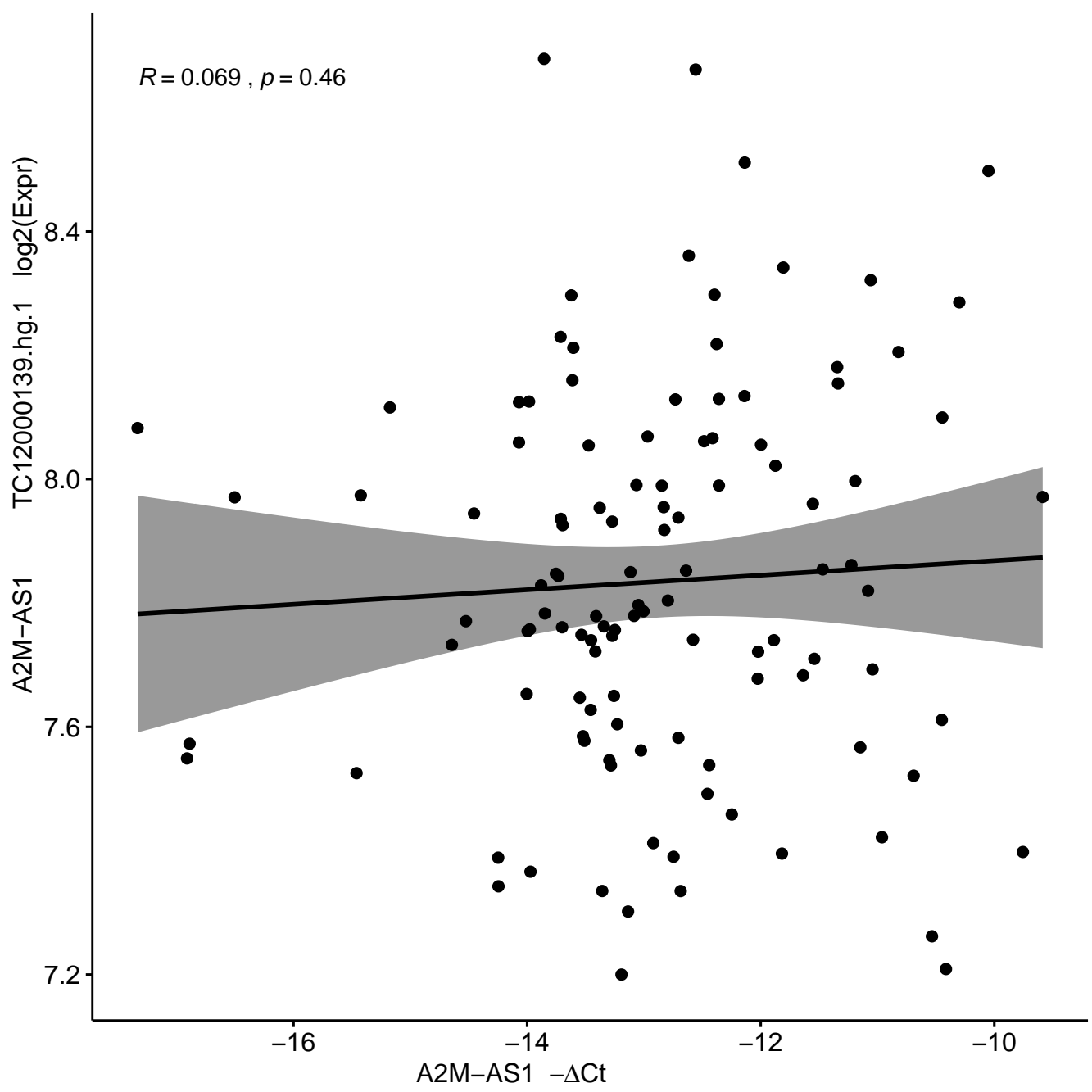

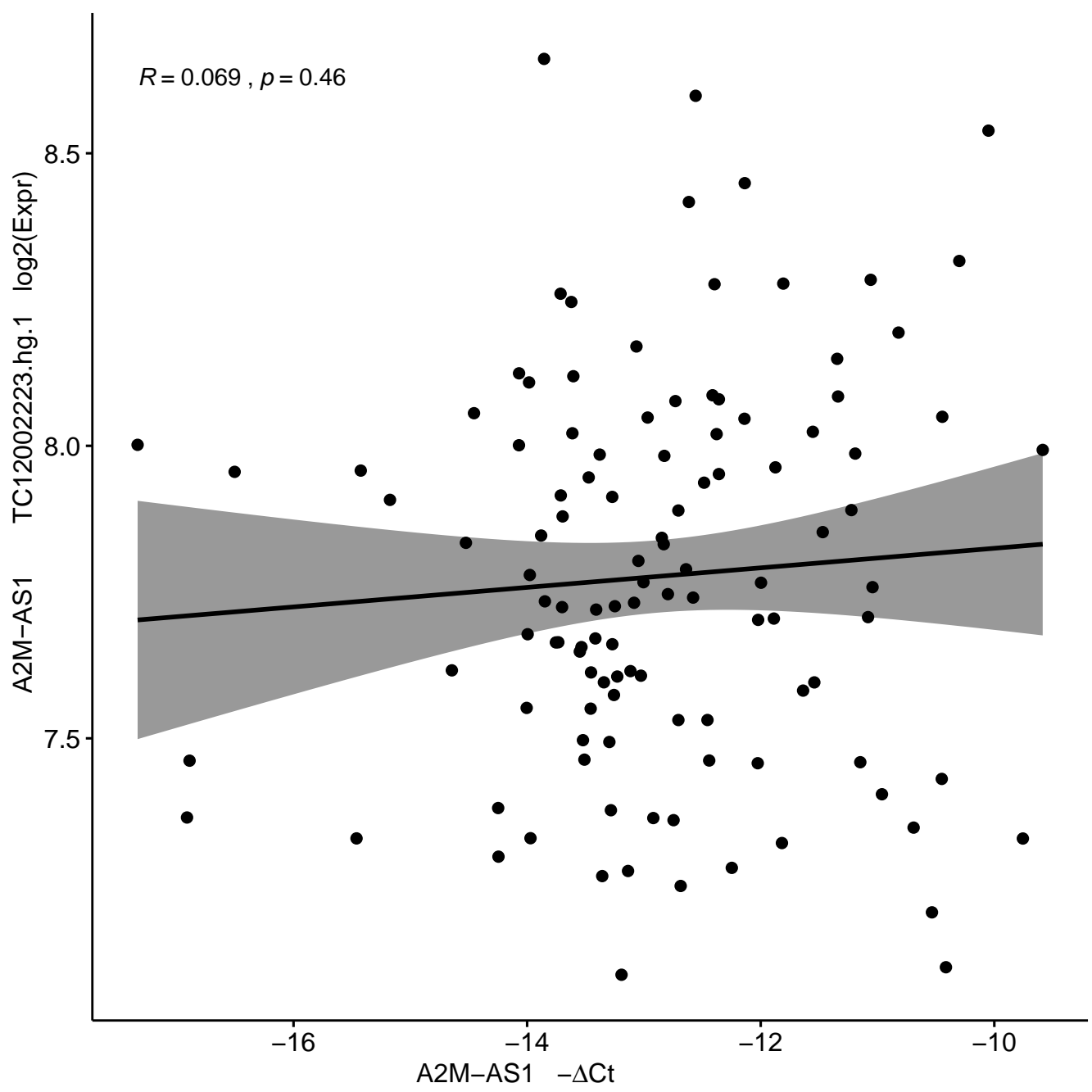

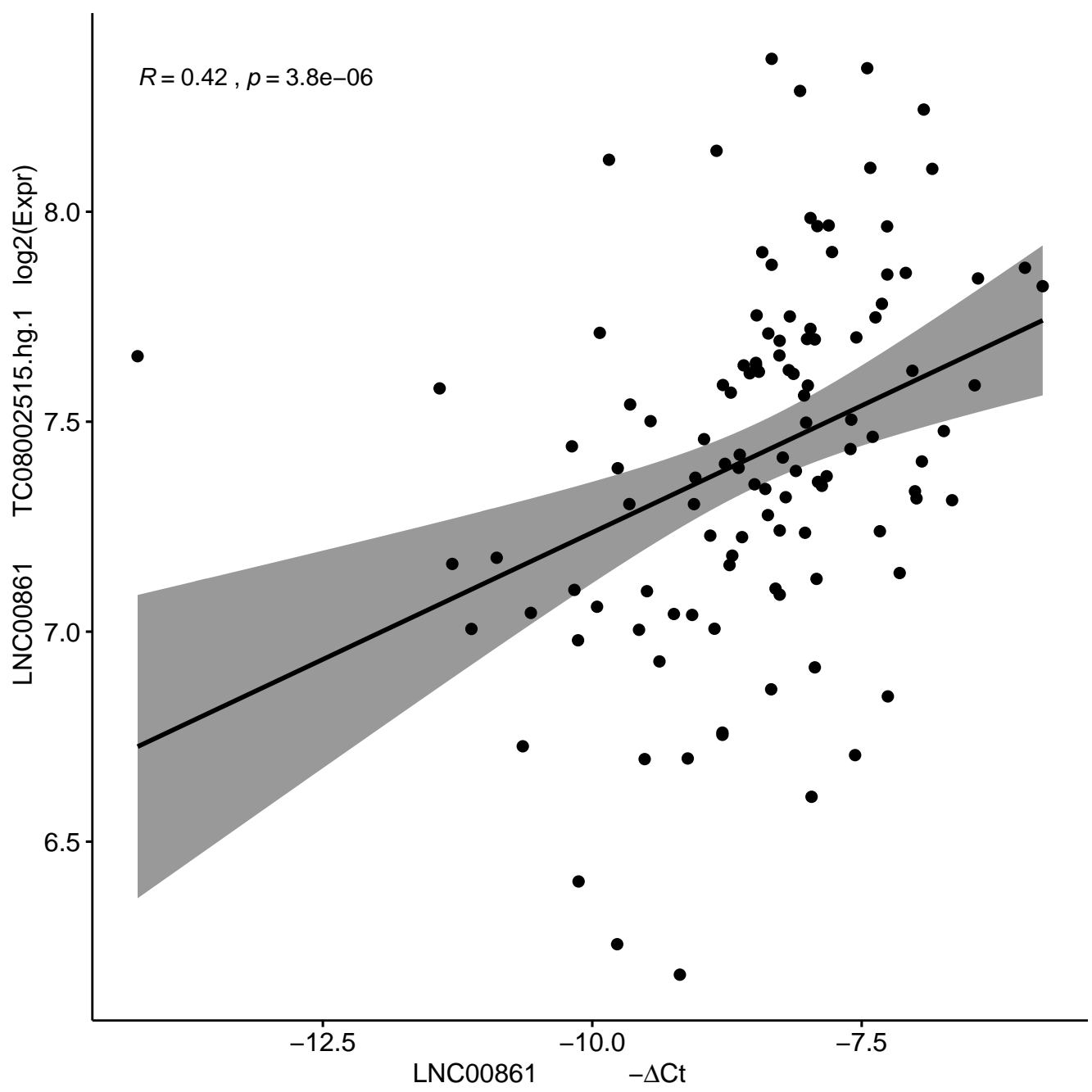

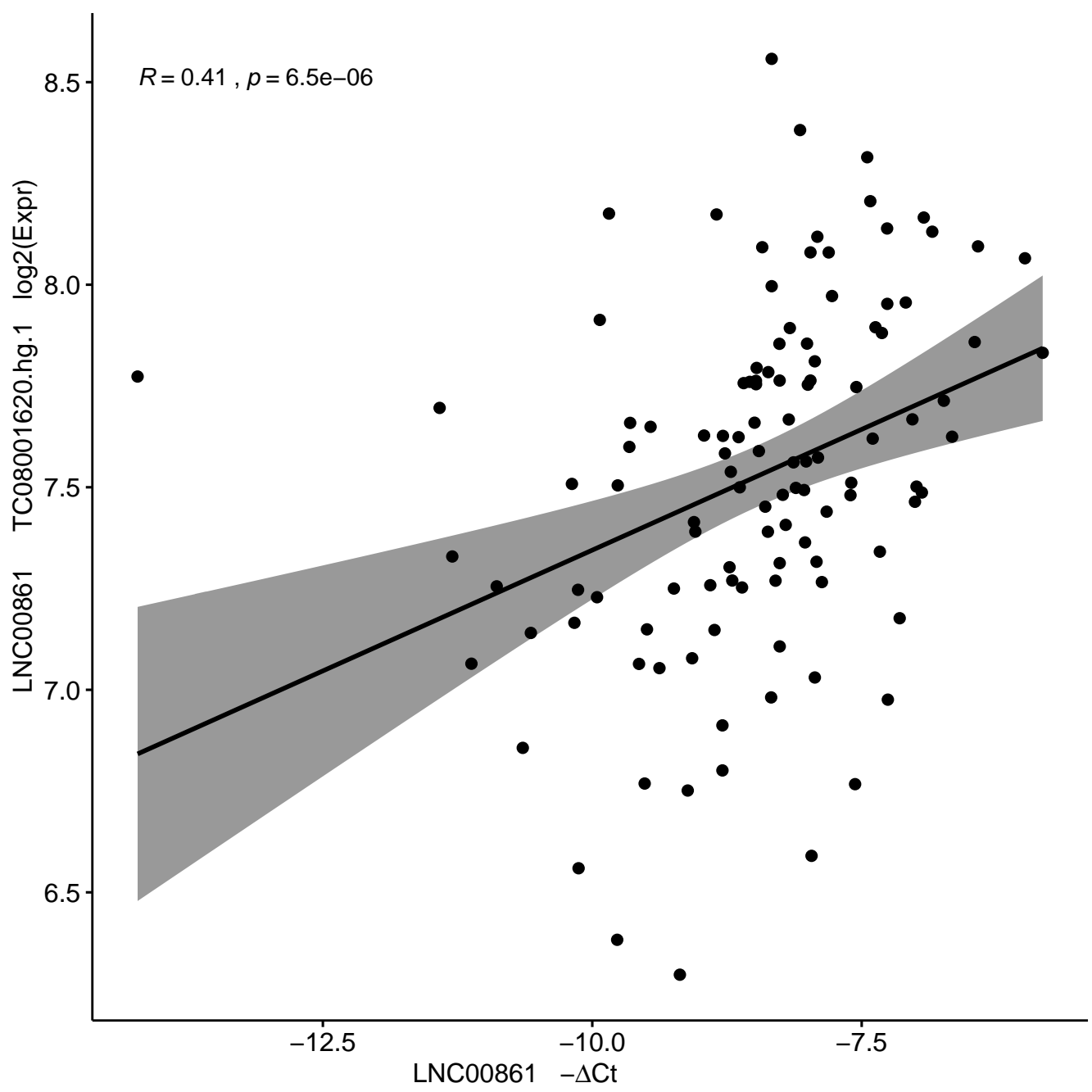

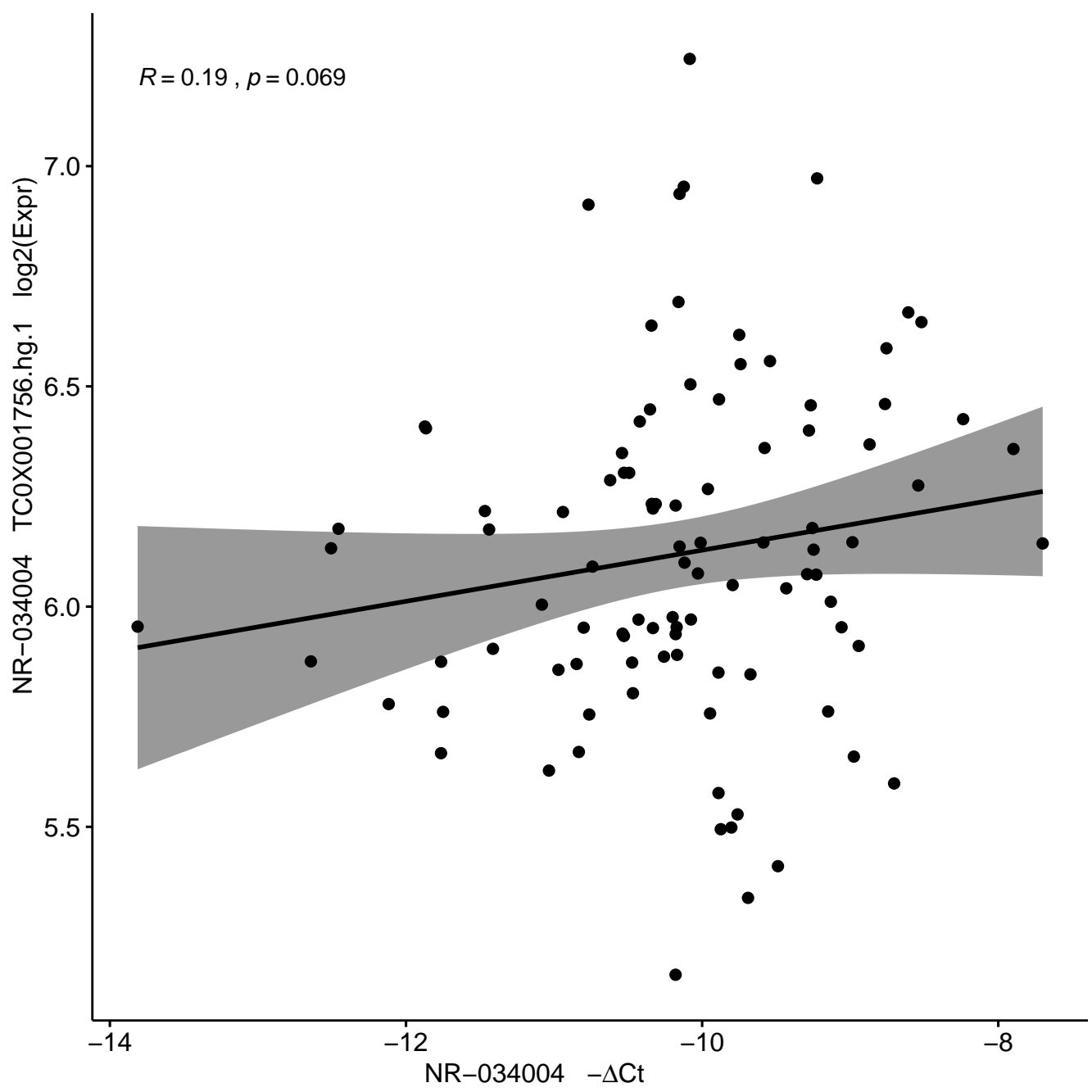

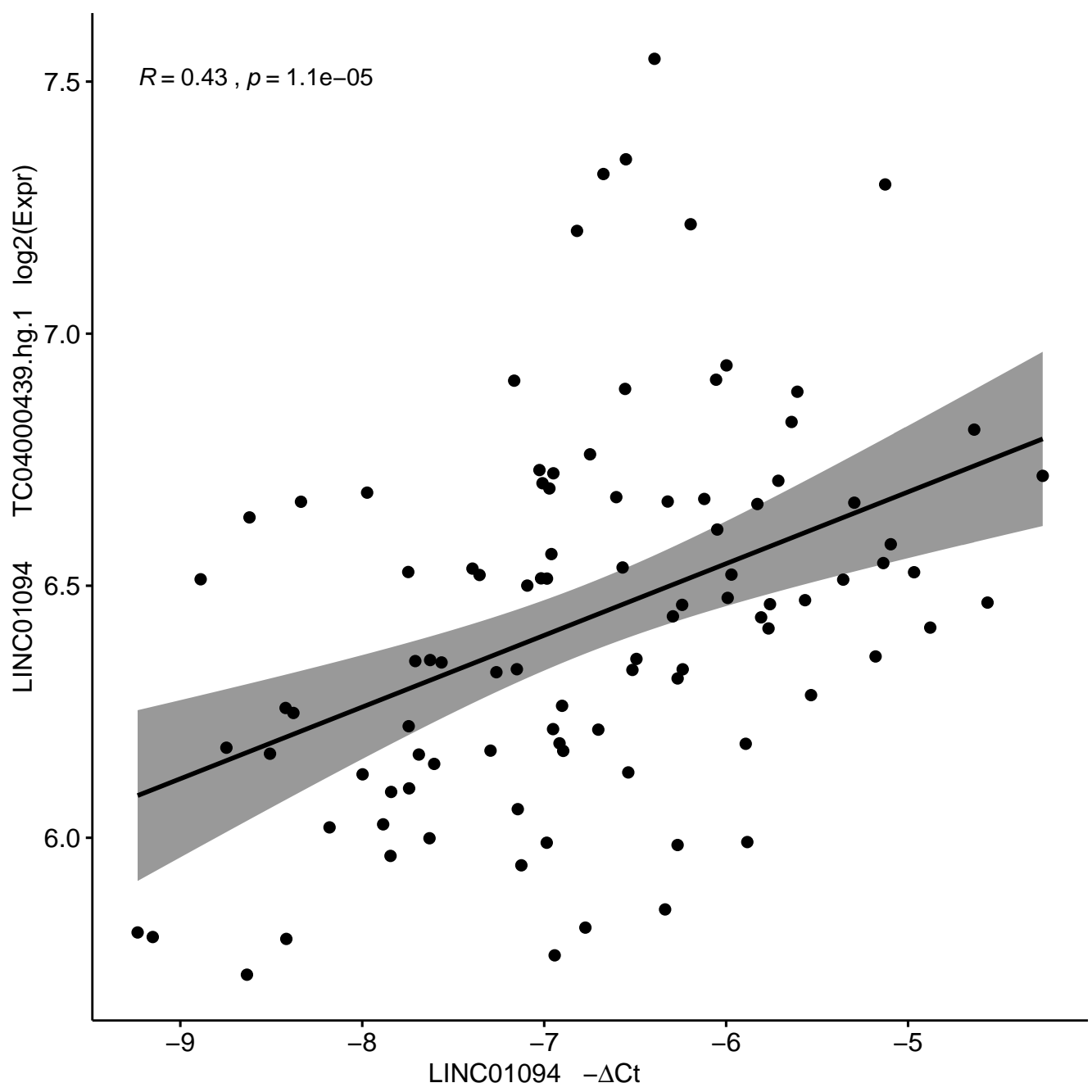

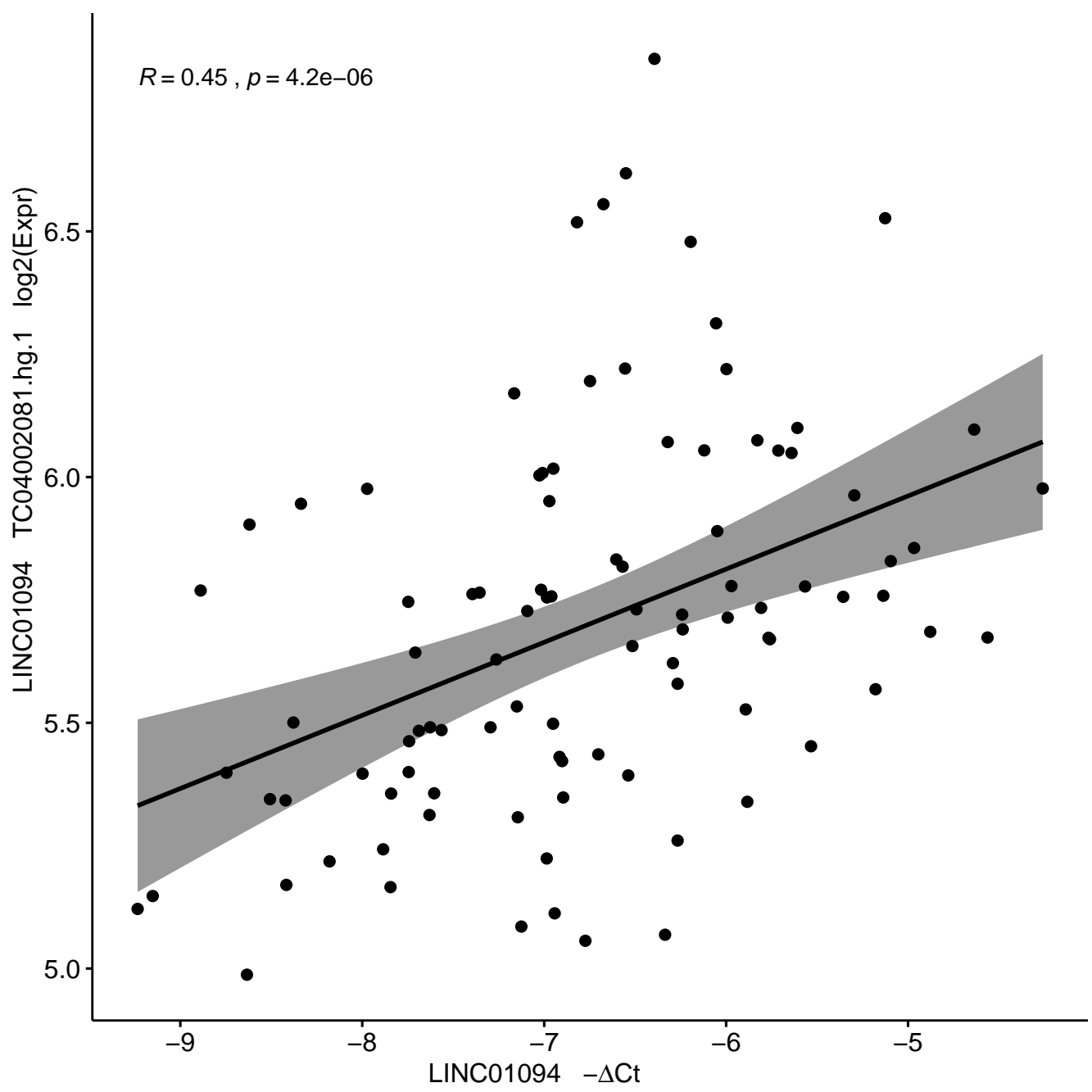

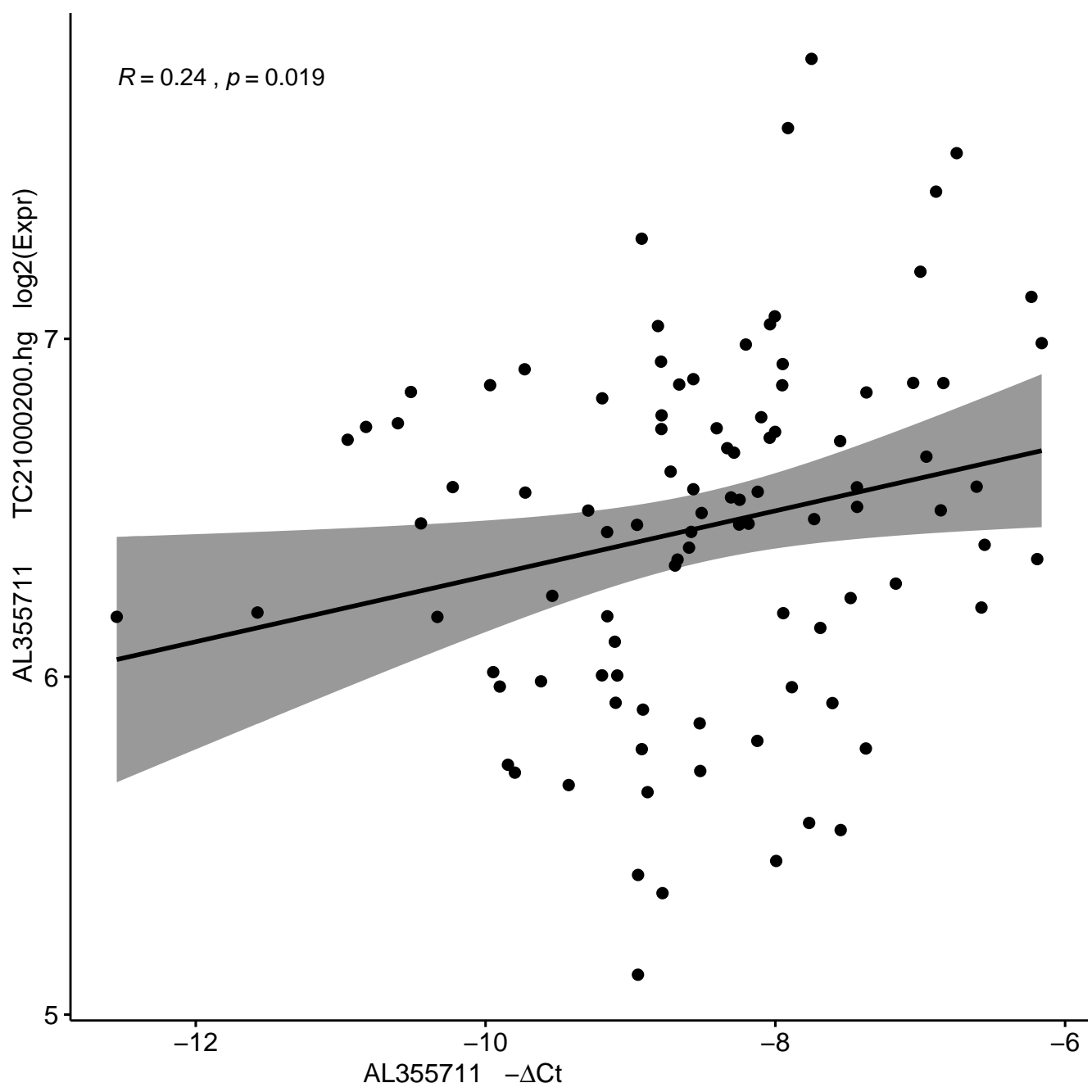

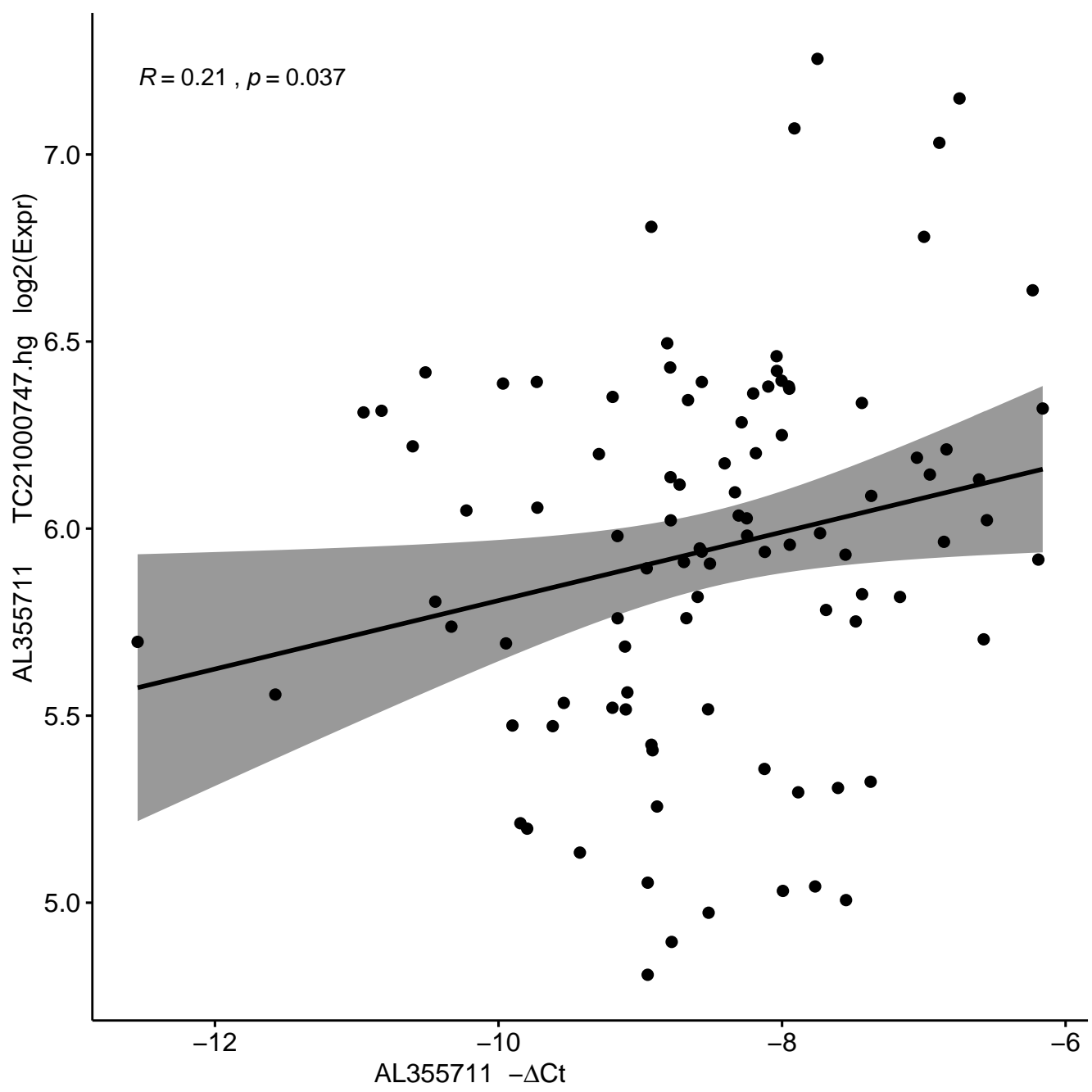

Supplement: Supplementary file 2 [file DataSheet4.PDF]

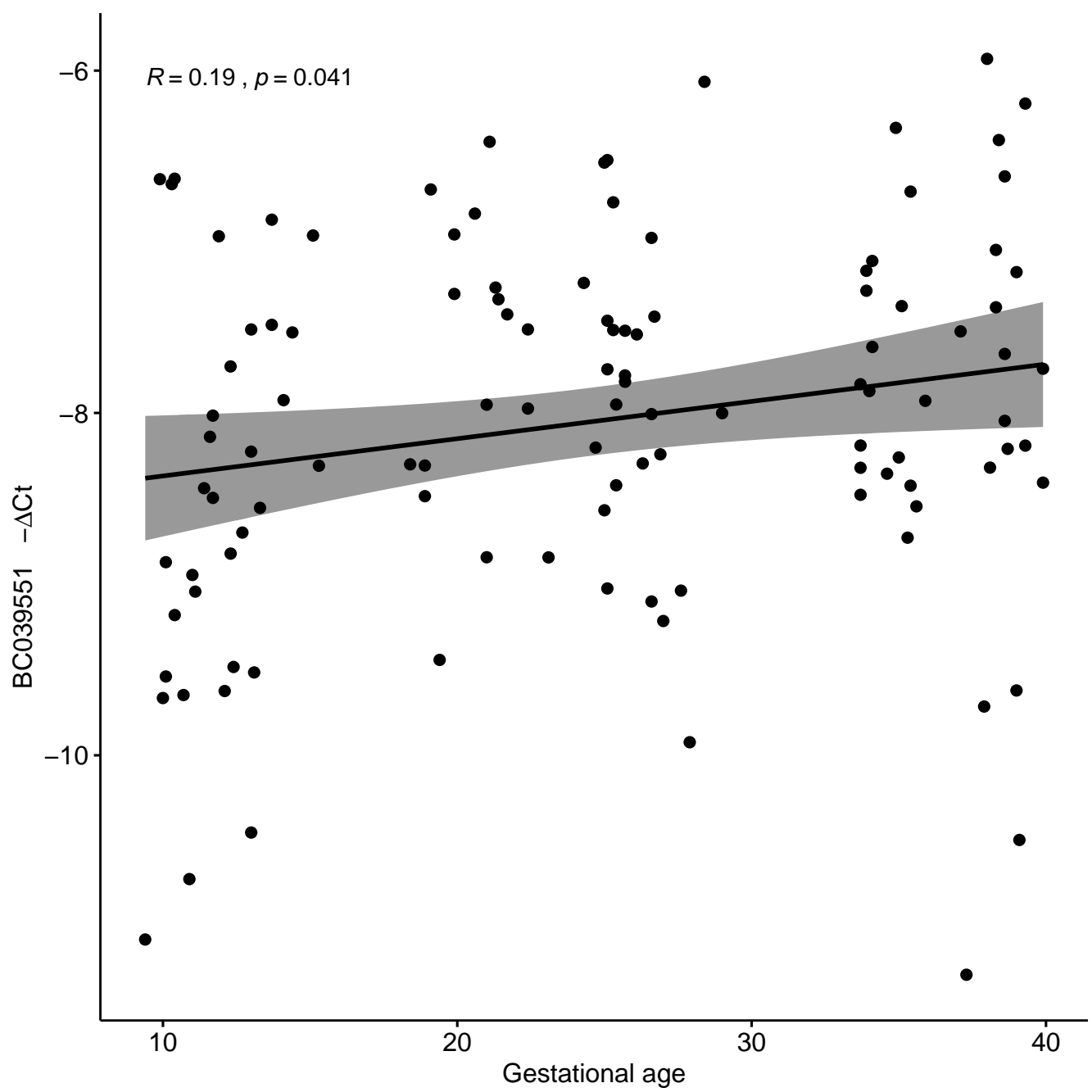

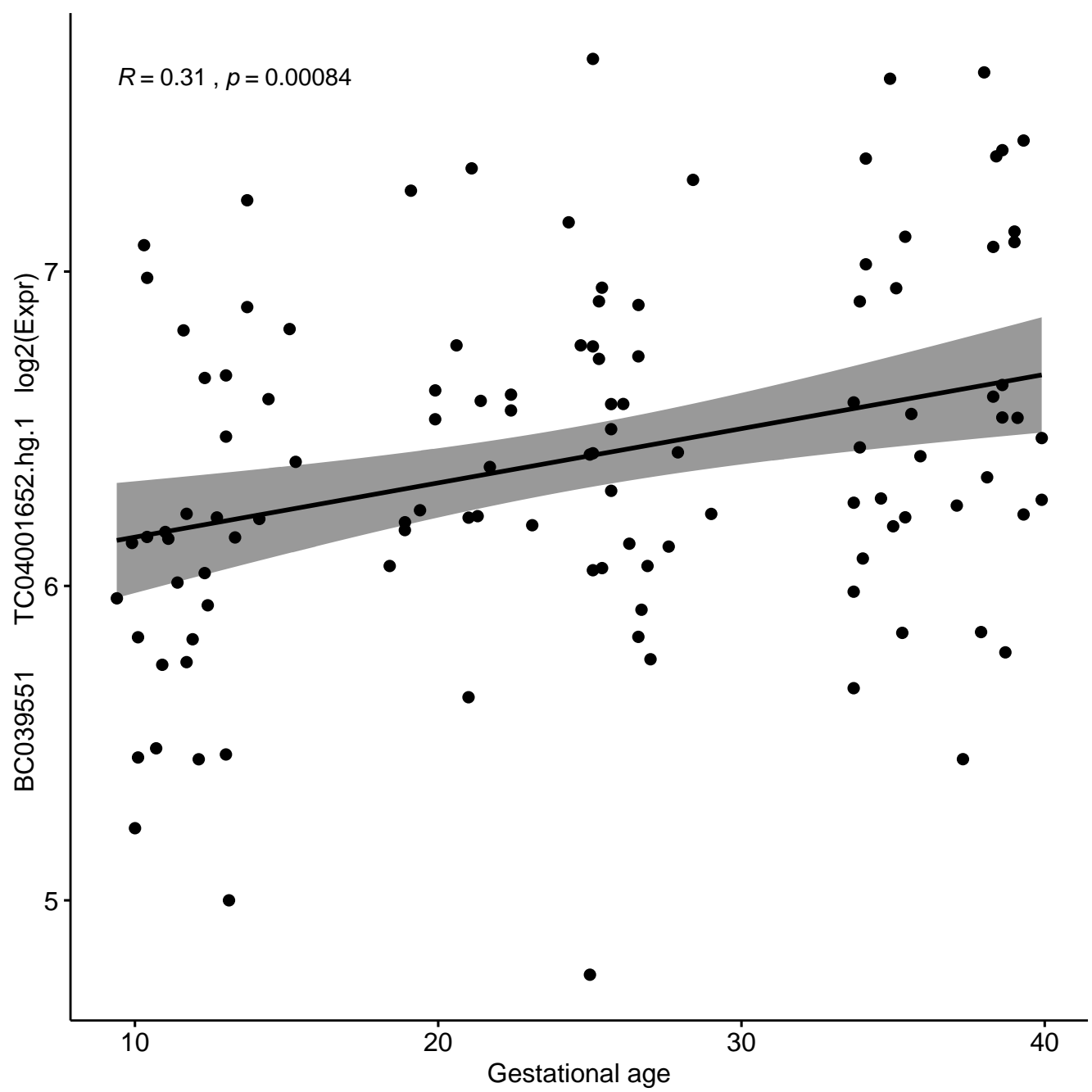

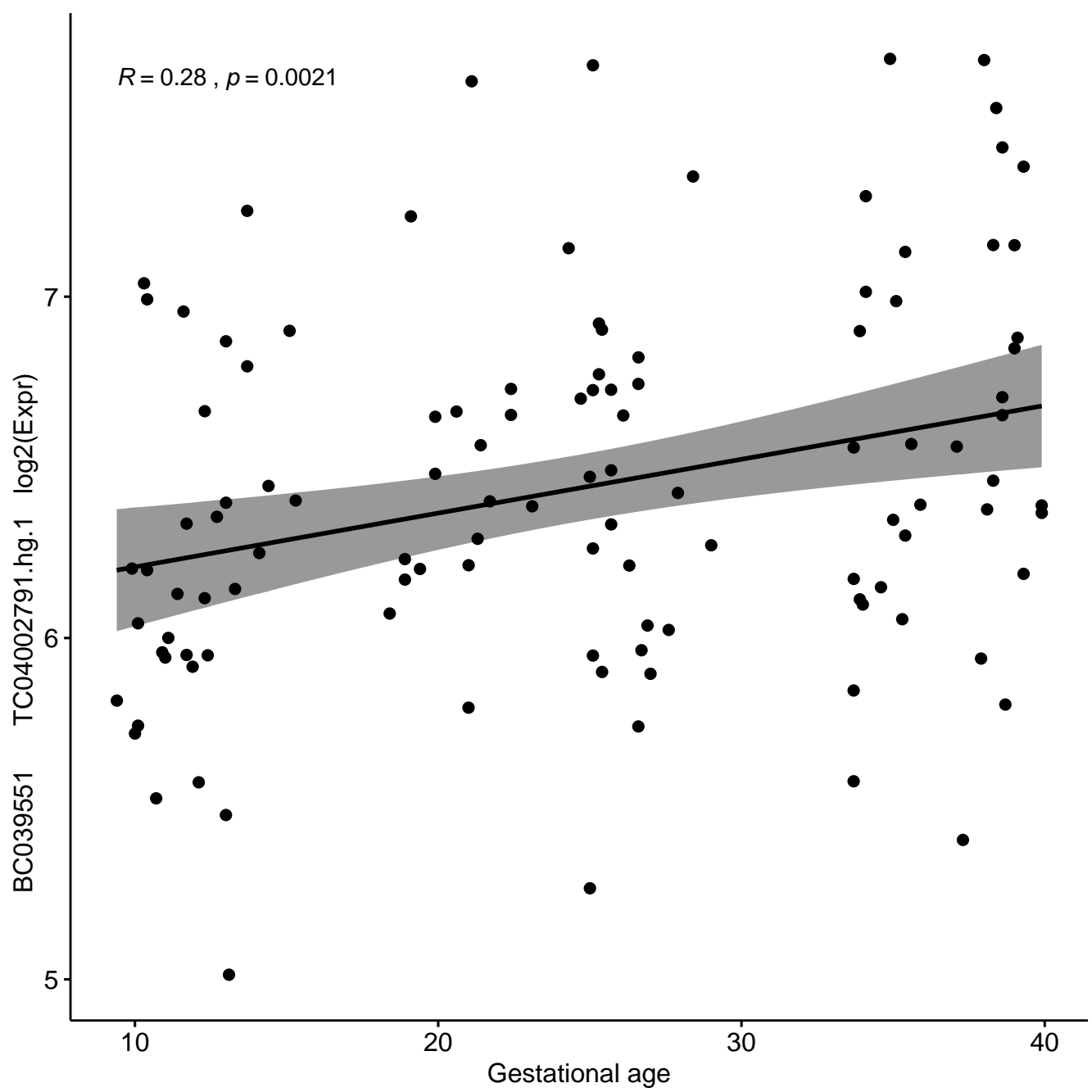

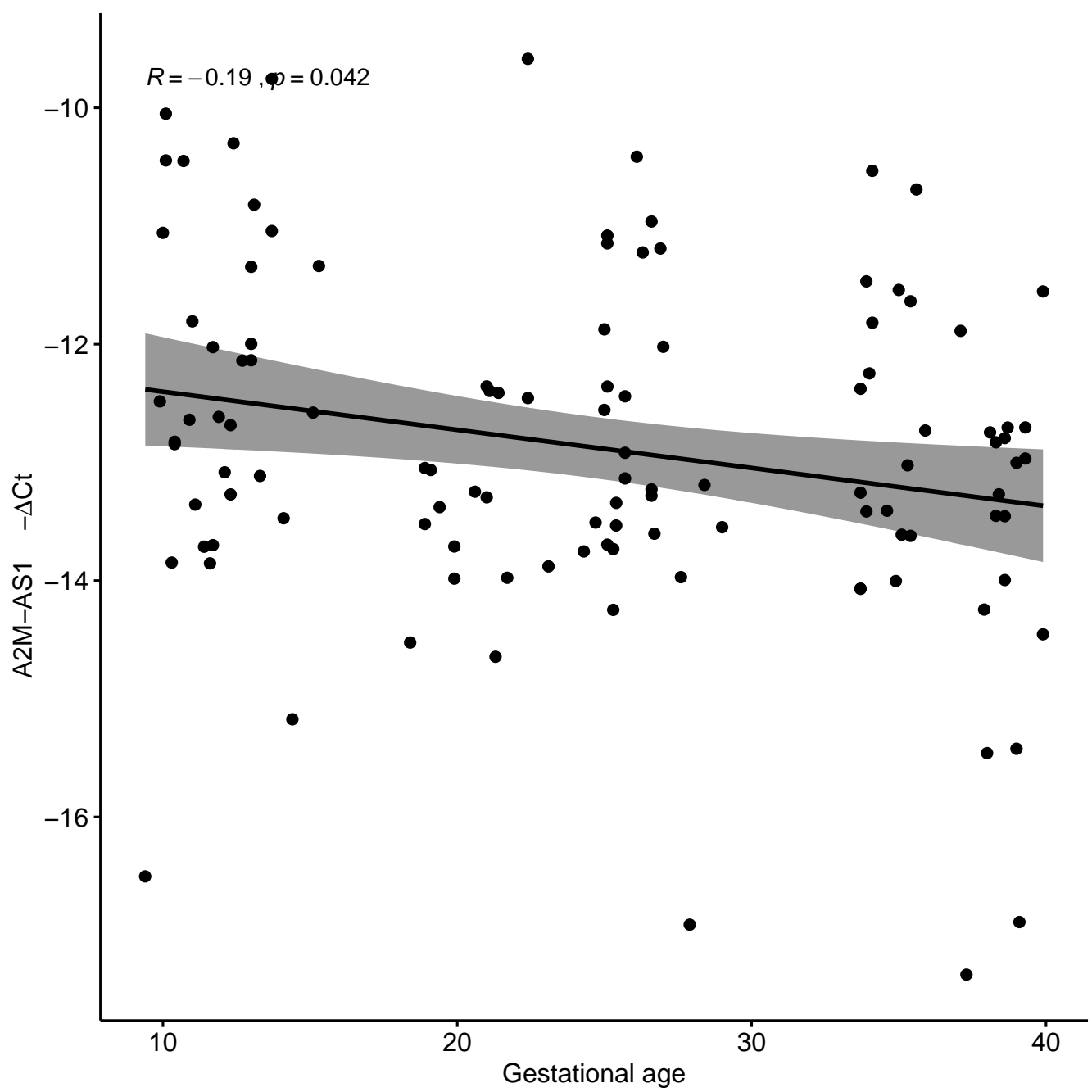

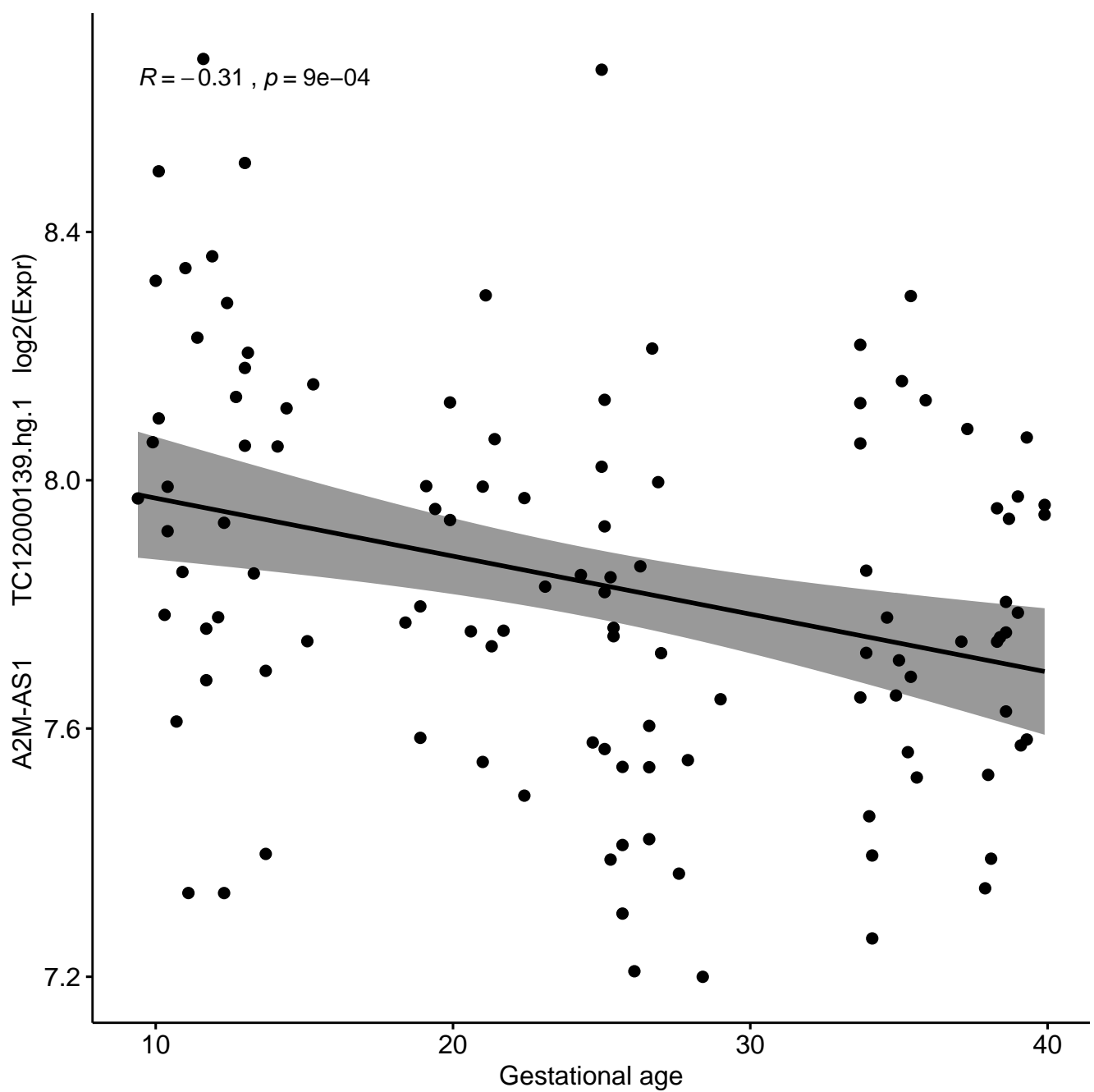

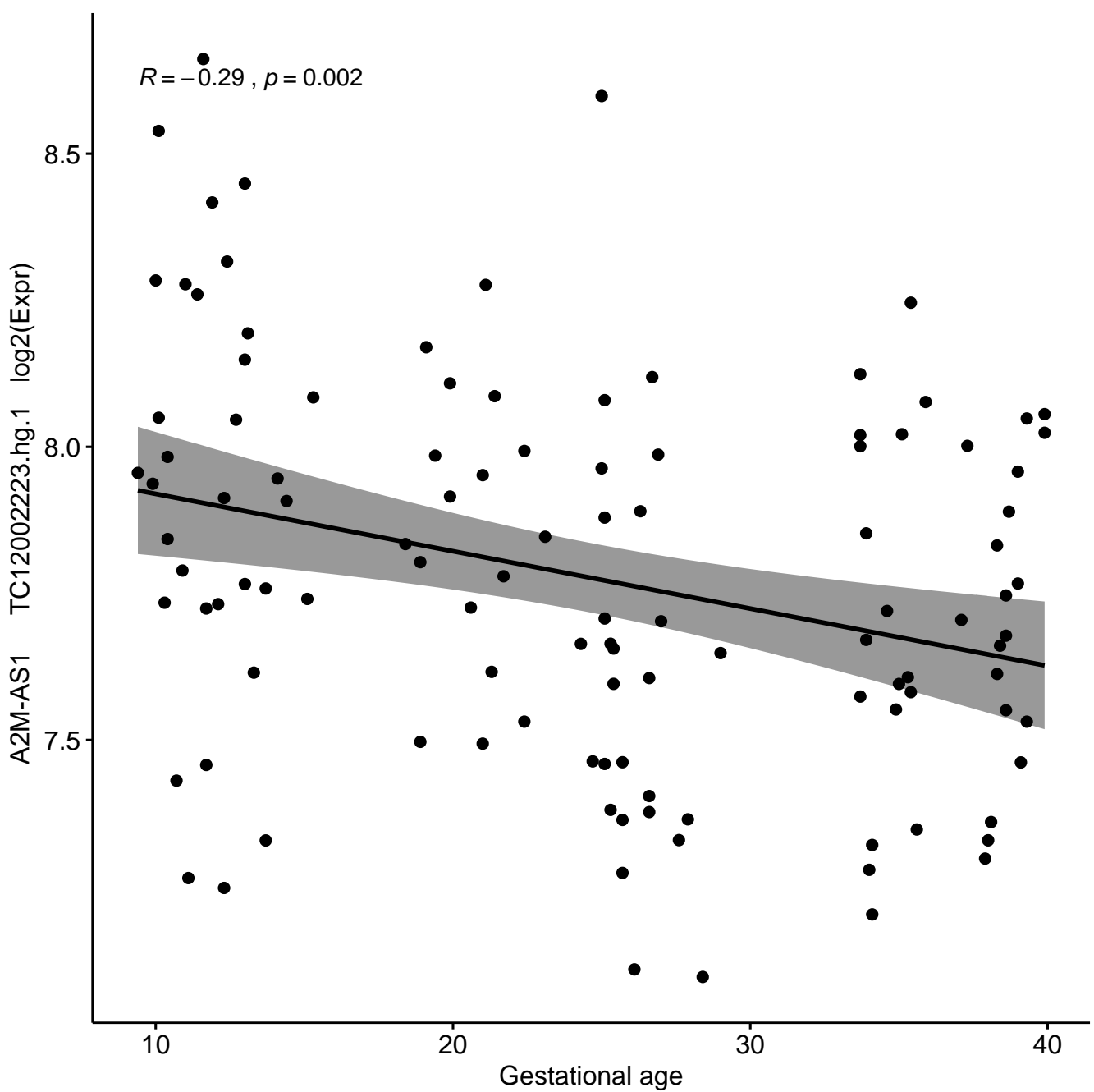

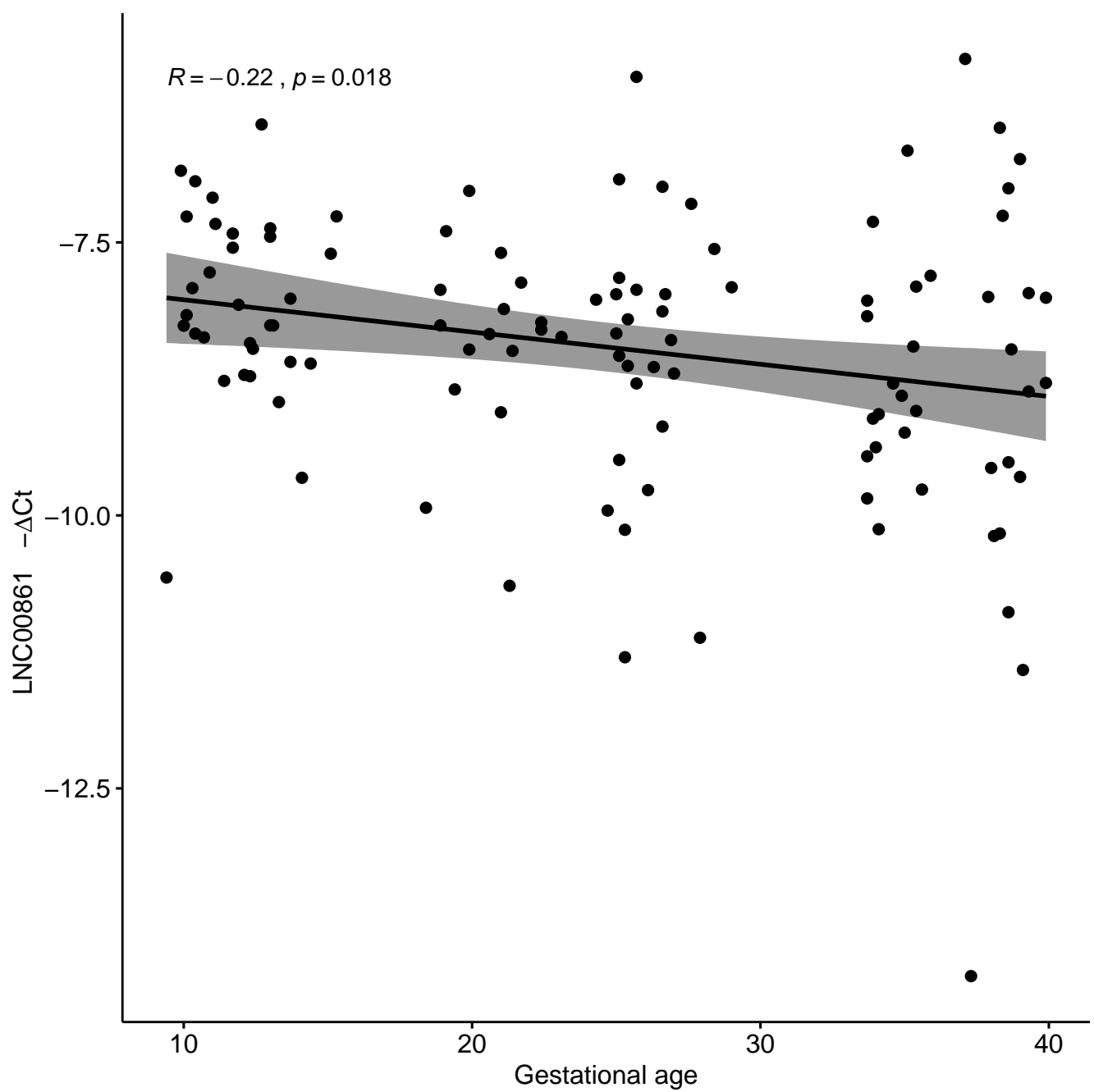

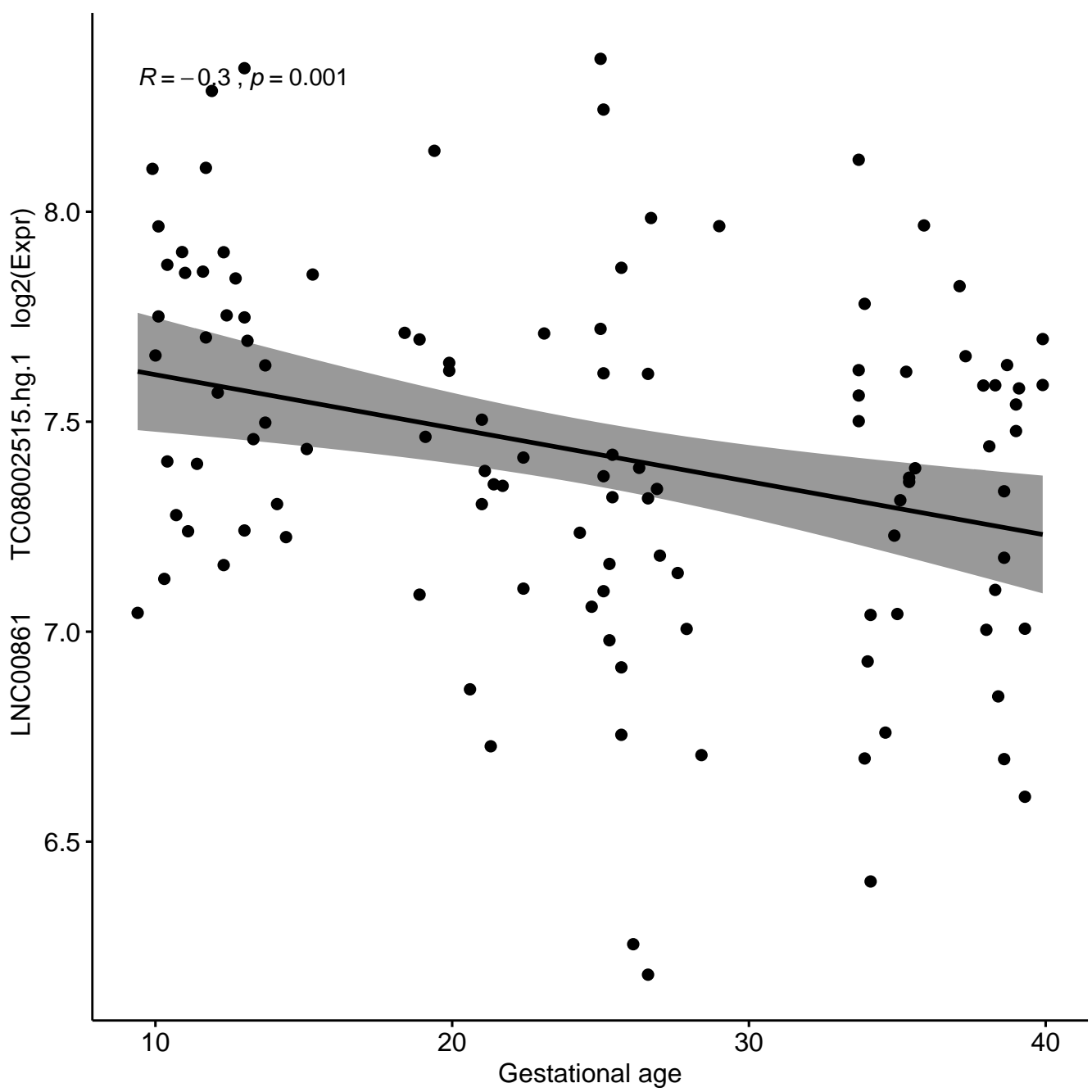

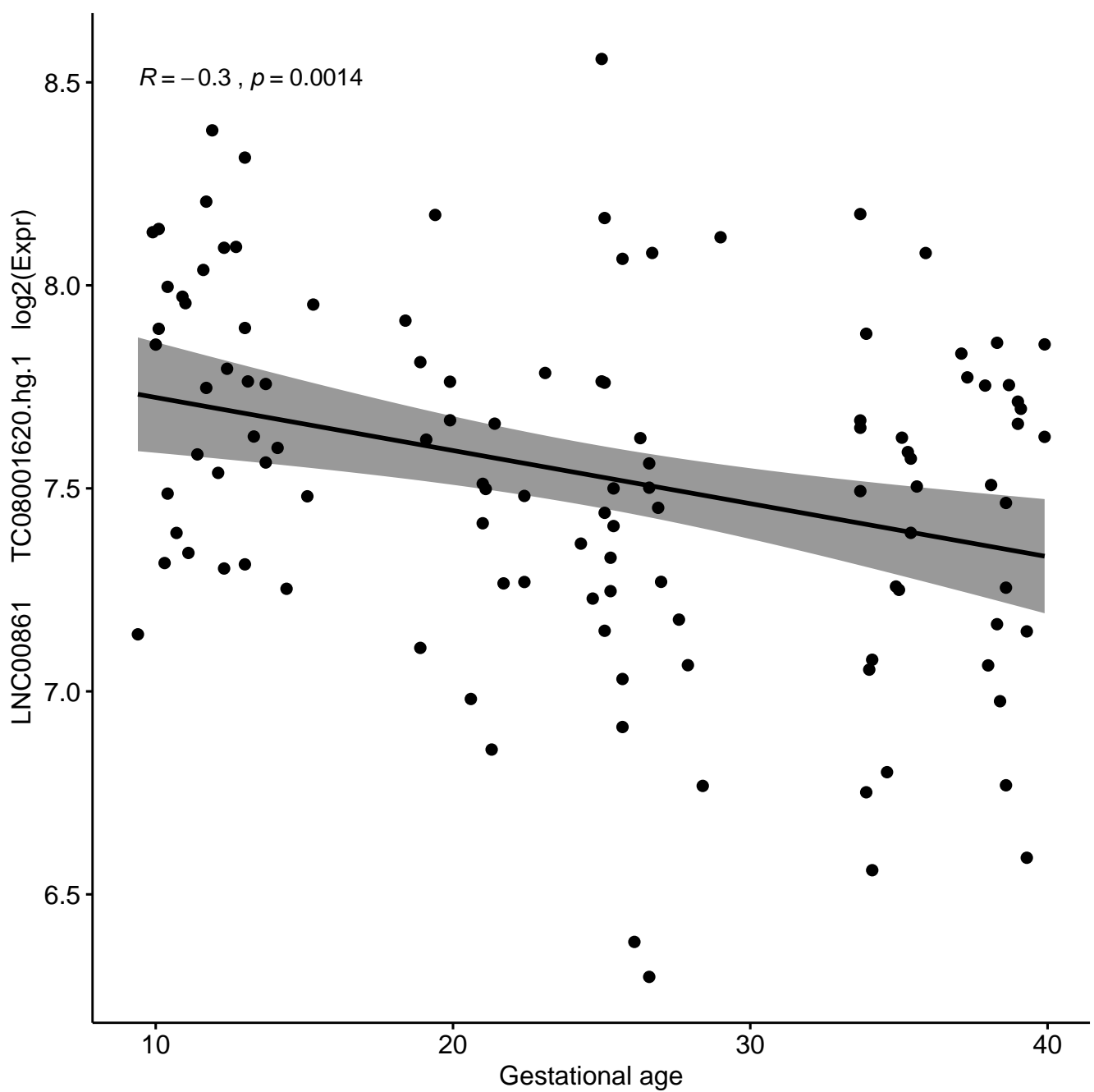

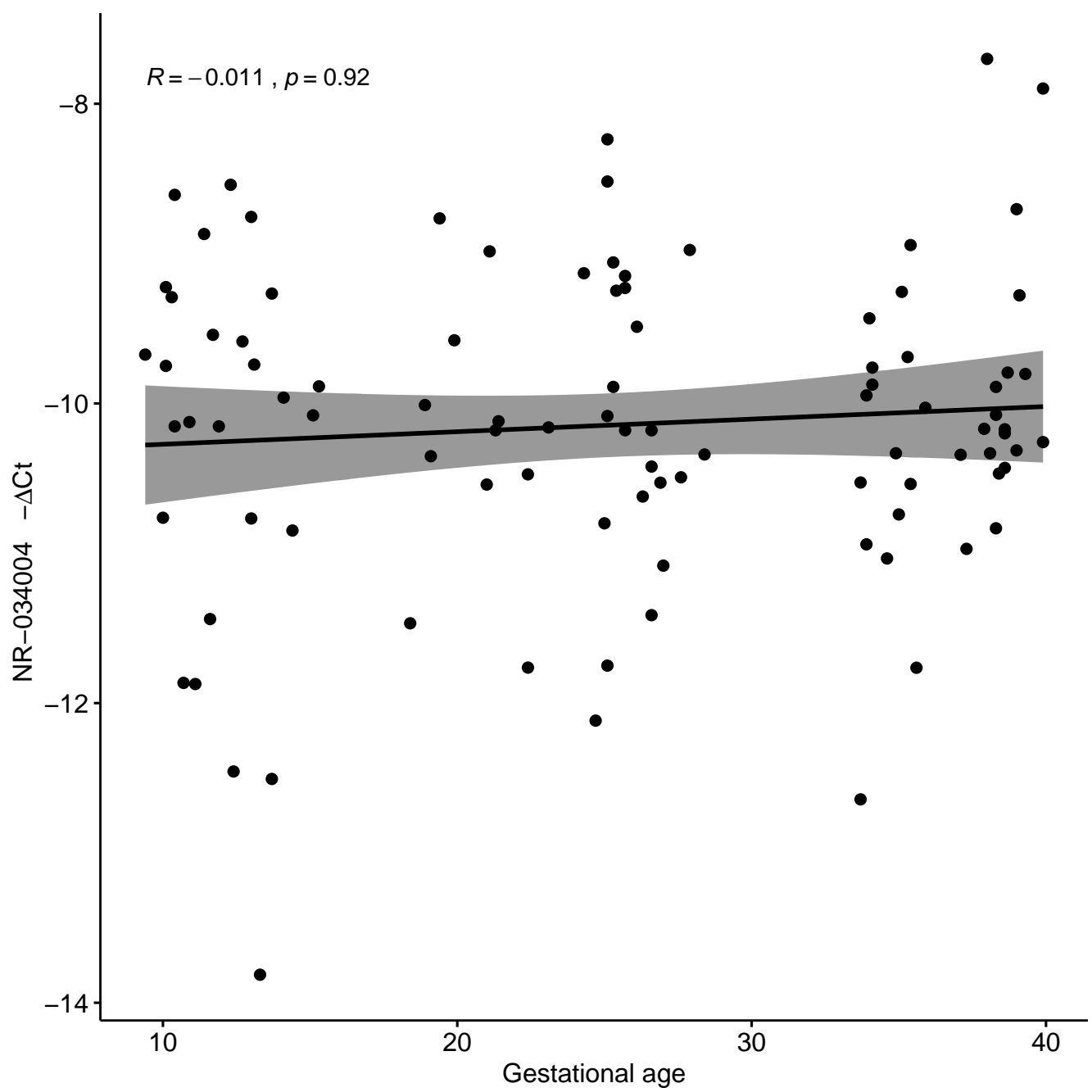

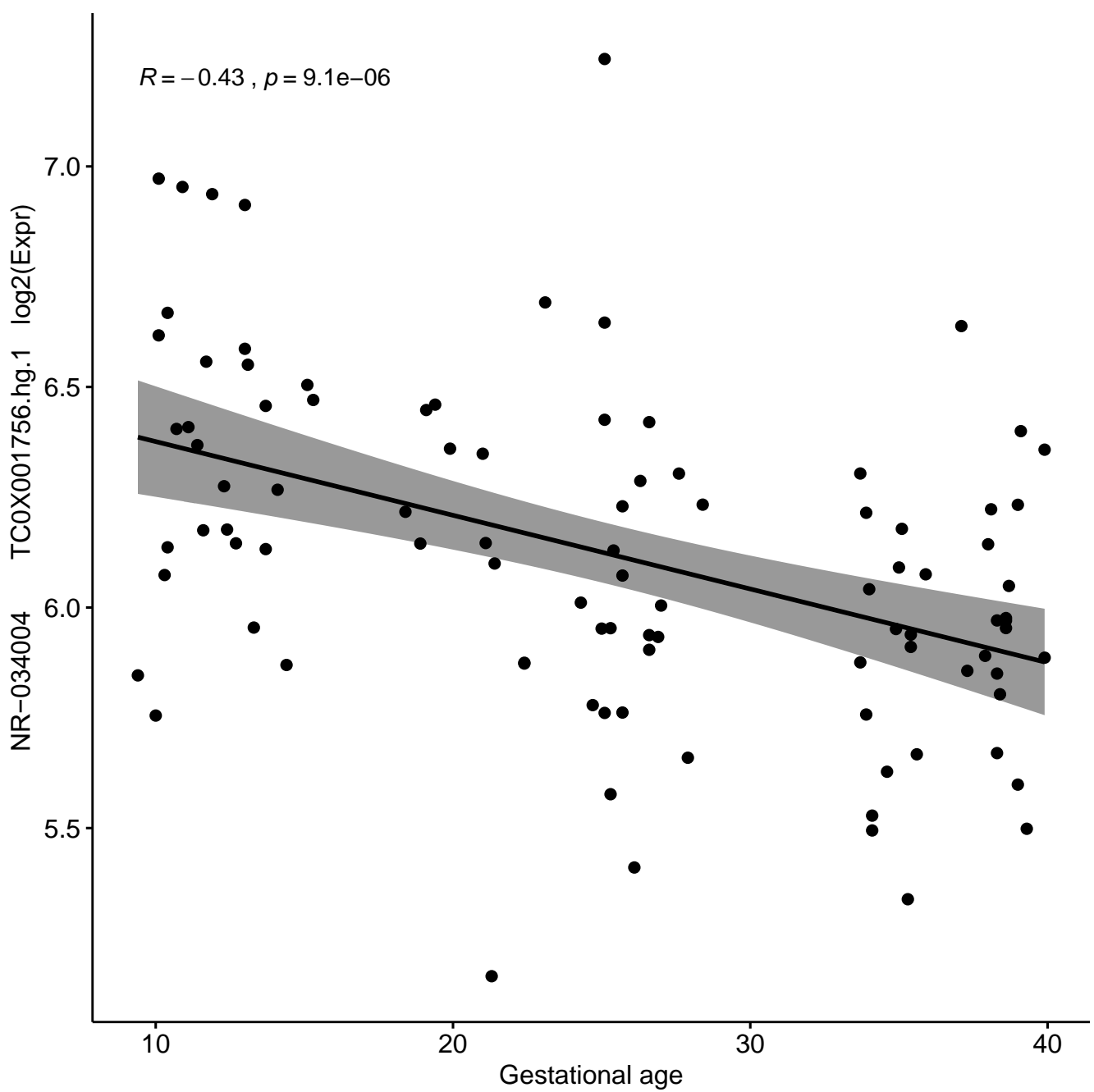

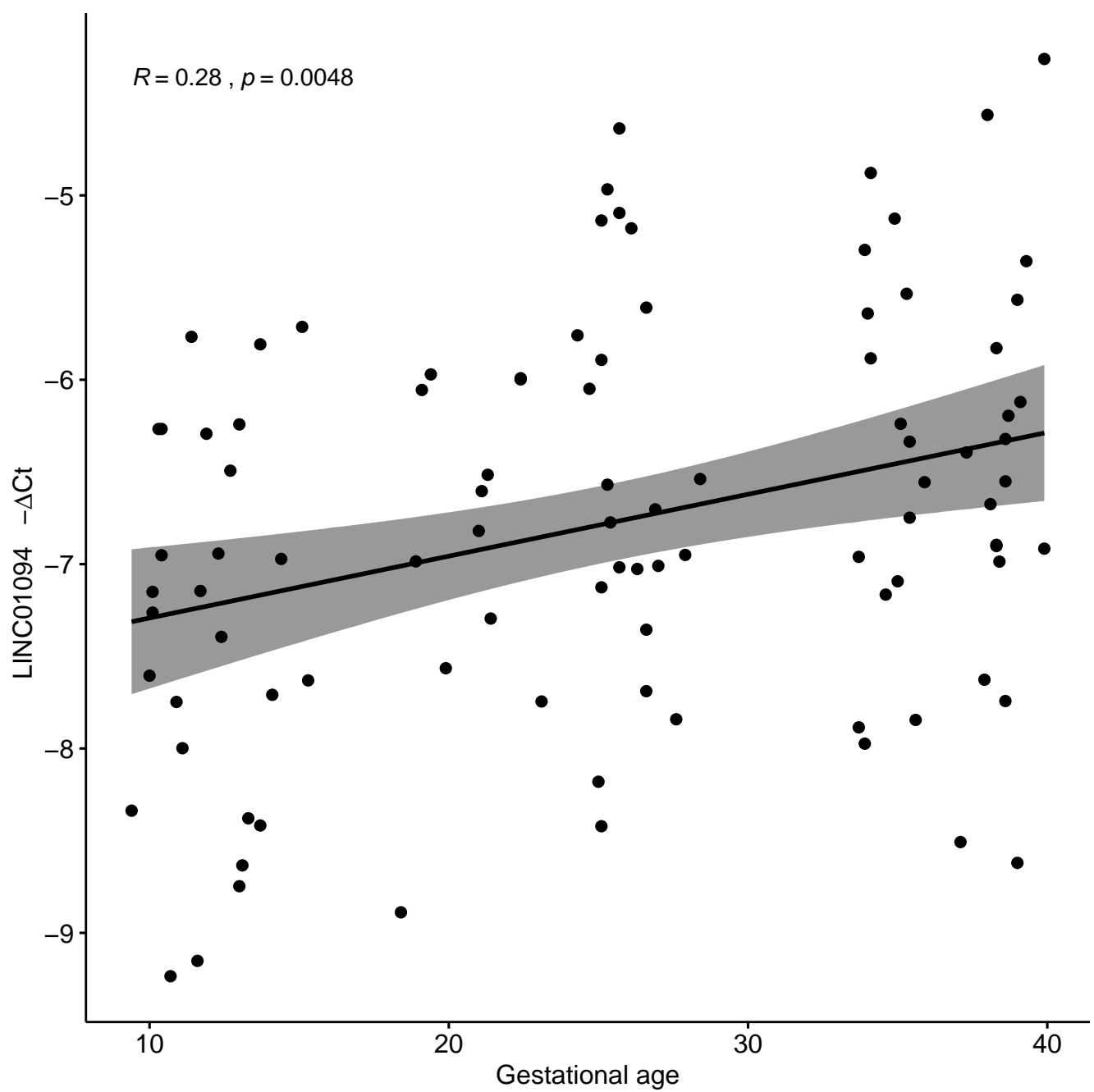

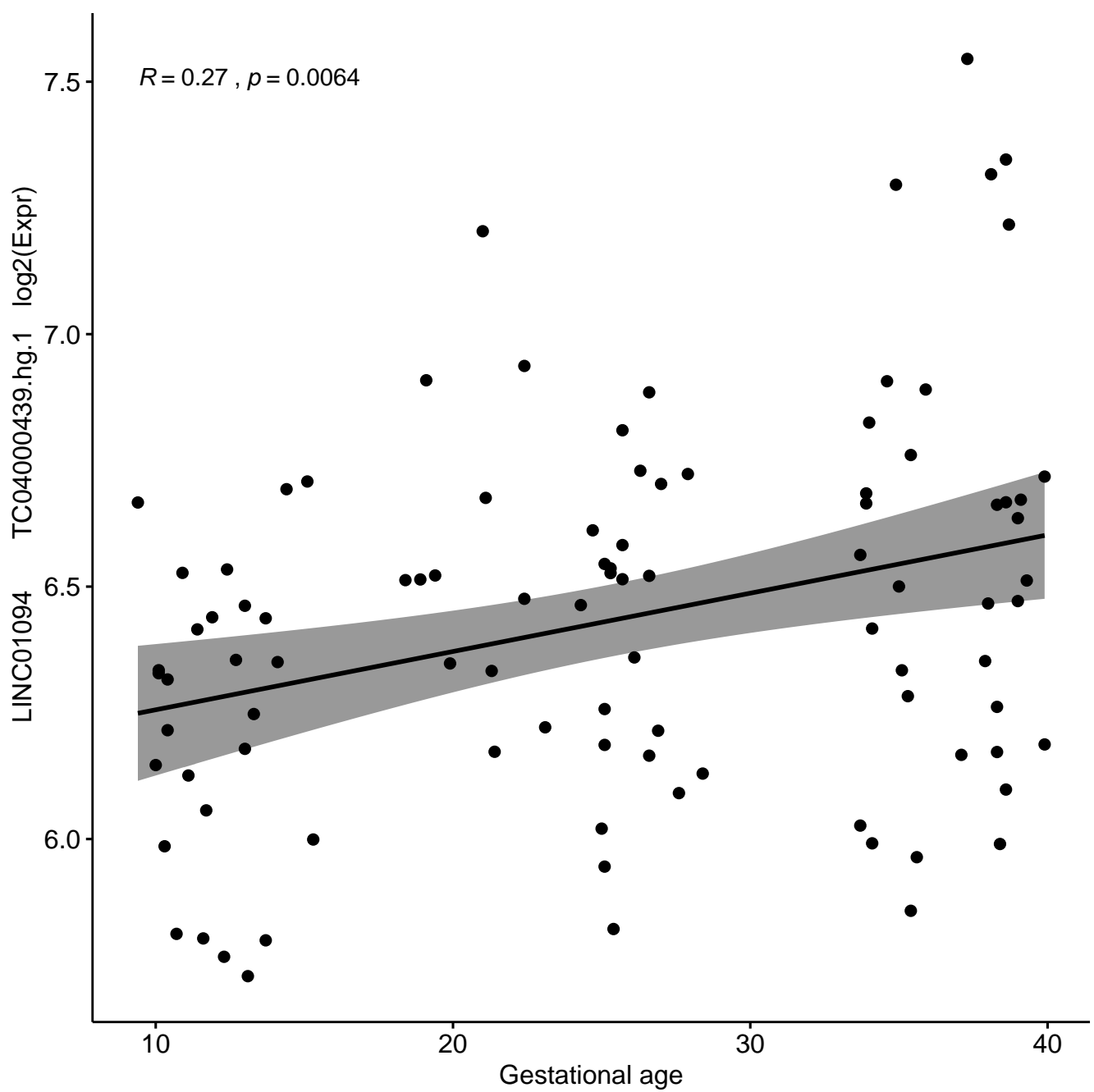

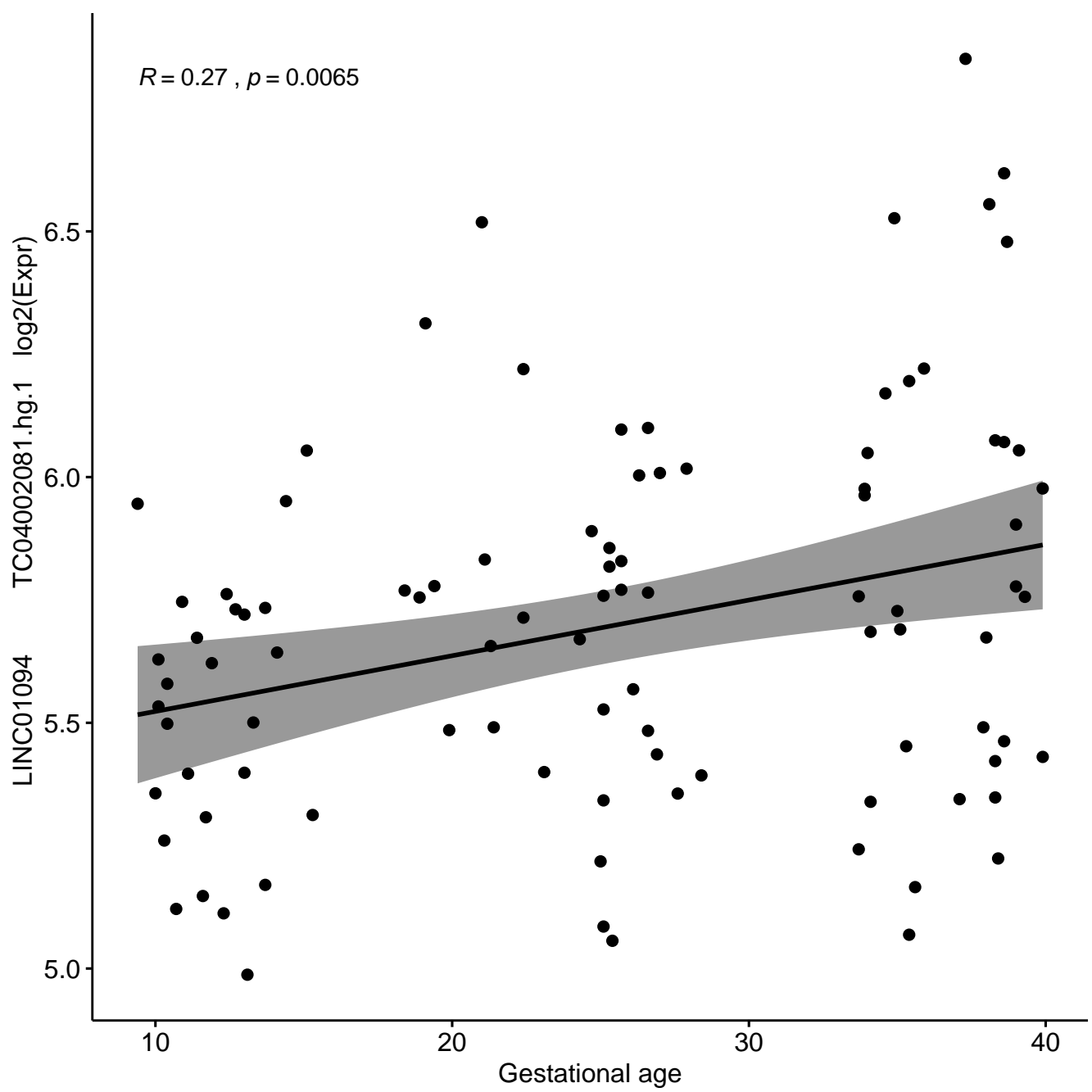

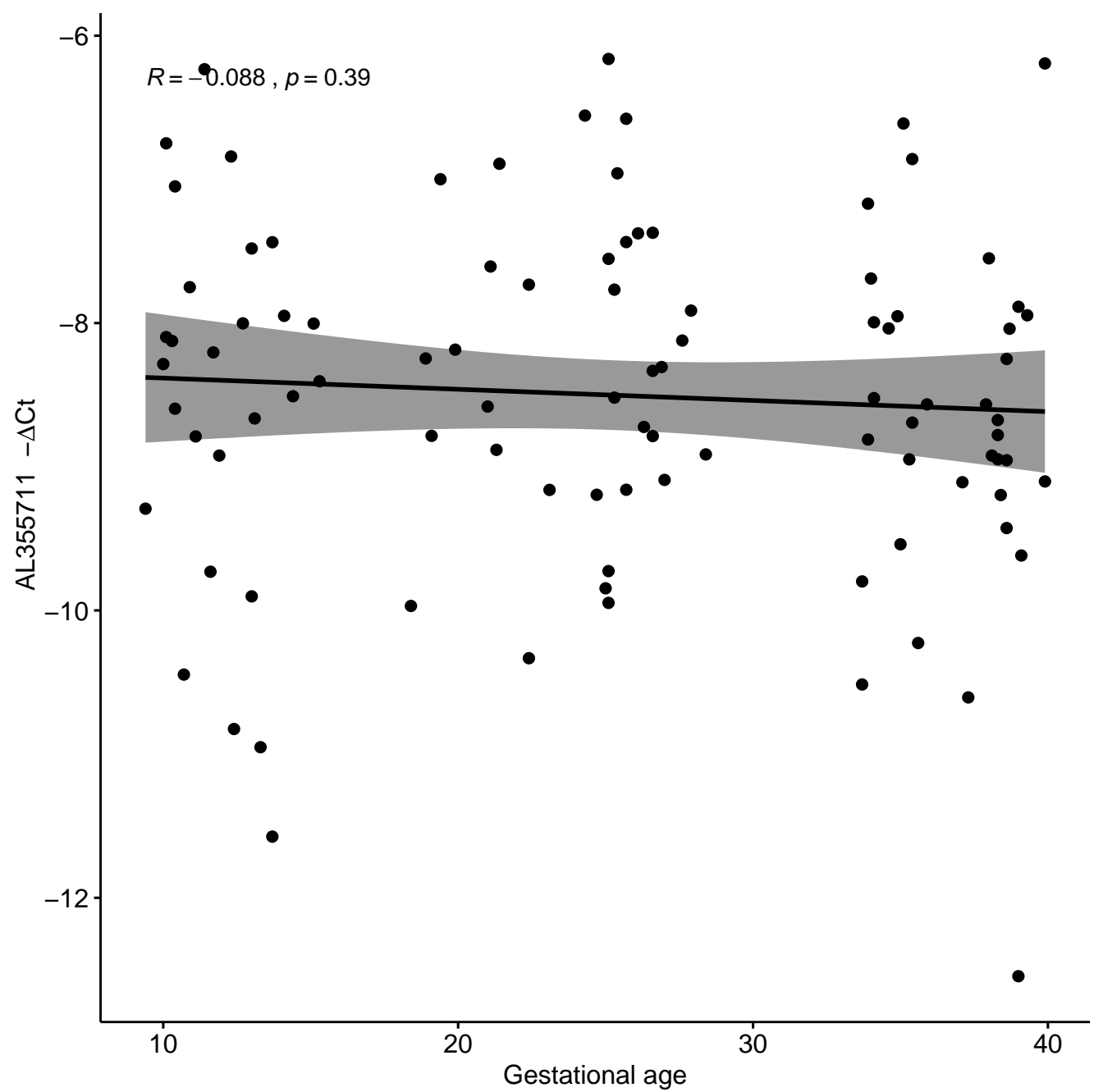

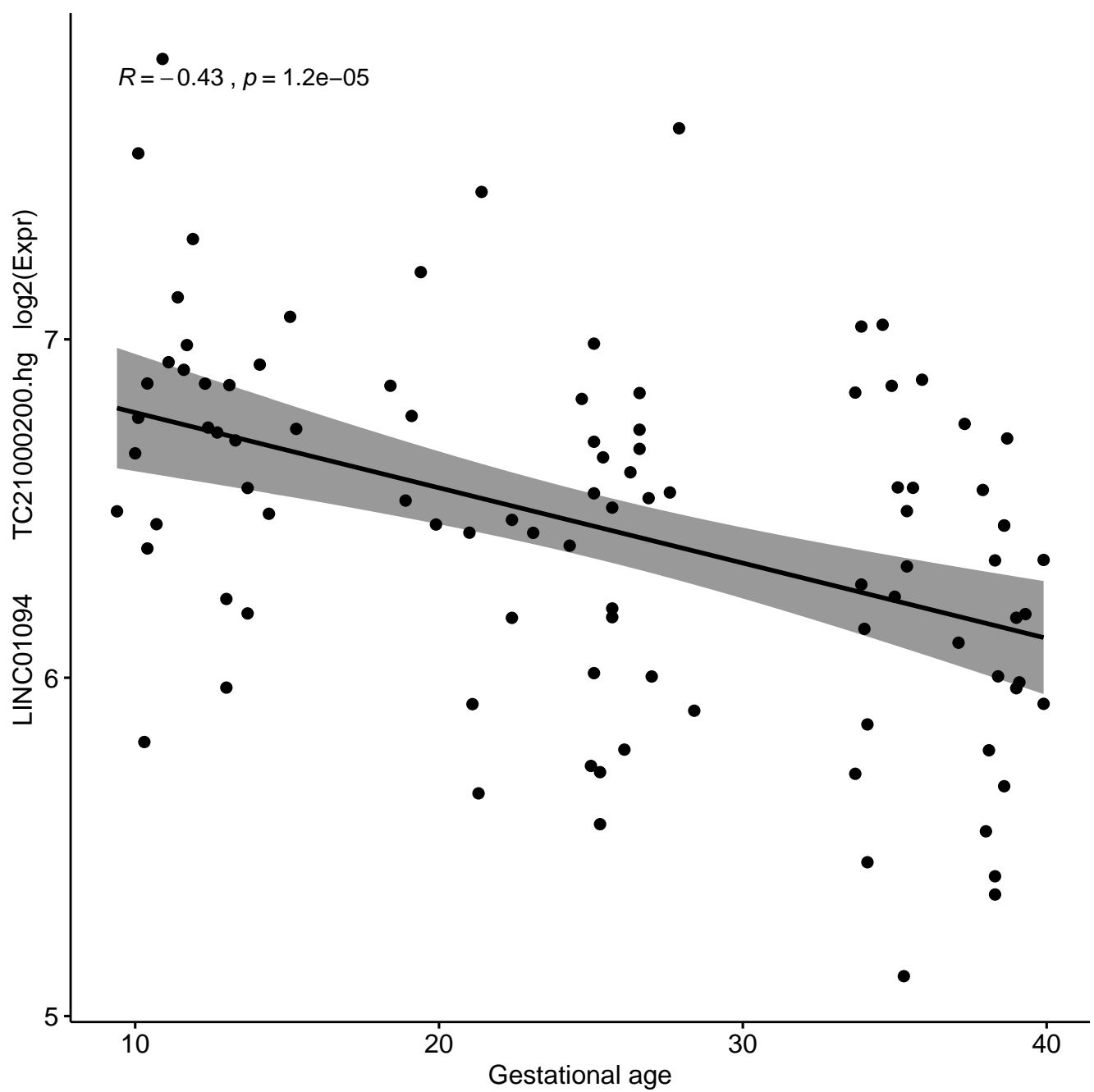

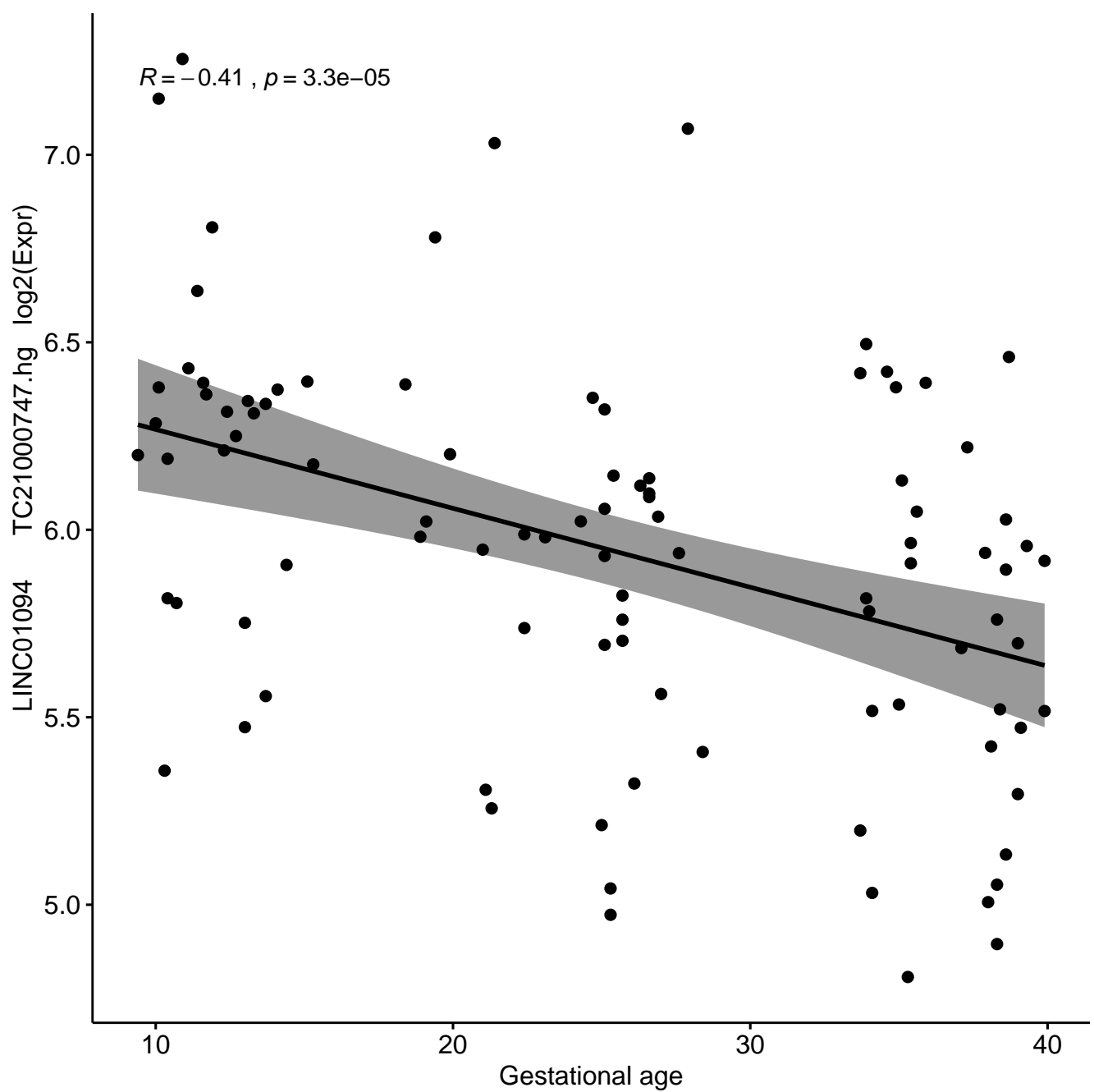

Supplement: Supplementary file 3 [file DataSheet3.PDF]
