# Supplementary material for: Gestational Age Dependence of the Maternal Circulating Long Non-Coding RNA Transcriptome During Normal Pregnancy Highlights Antisense and Pseudogene Transcripts
Source: Front Genet. 2021 Nov 22;12:760849. doi: 10.3389/fgene.2021.760849 (PMC8645989; doi:10.3389/fgene.2021.760849)
Supplement: Supplementary file 4 [file DataSheet1.PDF]

**Supplementary File 1**

| Assay ID | Gene Symbol | Dye | |
|---|---|---|---|
| Hs04939142_m1 | BC039551 | VIC-MGB | |
| Hs02786624_g1 | GAPDH | JUN-QSY | |
| Hs01394095_g1 | A2M-AS1 | ABY-QSY | |
| Hs04274413_m1 | LNC00861 | FAM-MGB | |
| ARRWGTF | NR_034004 | FAM-MGB | Custom designed use NR_034004 seq |
| Hs04232660_s1 | SCARNA2 | FAM-MGB | |
| Hs04976040_s1 | LINC01094 | FAM-MGB | |
| Hs01065959_s1 | AL355711 | FAM-MGB | |
