# Supplementary material for: Gestational Age Dependence of the Maternal Circulating Long Non-Coding RNA Transcriptome During Normal Pregnancy Highlights Antisense and Pseudogene Transcripts
Source: Front Genet. 2021 Nov 22;12:760849. doi: 10.3389/fgene.2021.760849 (PMC8645989; doi:10.3389/fgene.2021.760849)
Supplement: Supplementary file 5 [file DataSheet5.PDF]

Gestational age dependence of the maternal circulating long non-coding RNA transcriptome during normal pregnancy highlights antisense and pseudogene transcripts

Erica L. Kleinbrink[1], Nardhy Gomez-Lopez[2,3,4], Donghong Ju[1], Bogdan Done[2],
Anton-Scott Goustin[1], Adi L. Tarca[2,3,5], Roberto Romero[1,2,6,7,8*] and Leonard Lipovich[9*]

[1] Center for Molecular Medicine and Genetics, Wayne State University, Detroit, MI, United States.
[2] Perinatology Research Branch, Division of Obstetrics and Maternal-Fetal Medicine, Division of Intramural Research, *Eunice Kennedy Shriver* National Institute of Child Health and Human Development, National Institutes of Health, U.S. Department of Health and Human Services, Bethesda, MD, and Detroit, MI, United States.
[3] Department of Obstetrics and Gynecology, Wayne State University School of Medicine, Detroit, MI, United States.
[4] Department of Biochemistry, Microbiology, and Immunology, Wayne State University School of Medicine, Detroit, MI, United States.
[5] Department of Computer Science, Wayne State University College of Engineering, Detroit, MI, United States.
[6] Department of Obstetrics and Gynecology, University of Michigan, Ann Arbor, MI, United States.
[7] Department of Epidemiology and Biostatistics, Michigan State University, East Lansing, MI, United States.
[8] Detroit Medical Center, Detroit, MI, United States.
[9] Department of Basic Sciences, College of Medicine, Mohammed Bin Rashid University of Medicine and Health Sciences, Dubai, United Arab Emirates.

***Correspondence:**

Leonard Lipovich, leonard.lipovich@mbru.ac.ae;
Roberto Romero, prbchiefstaff@med.wayne.edu

Supplement Table of Contents

Supplementary Figures

Supplementary Figure 1. FANTOM5 Consortium ZENBU Browser view of human tissues and cell types with the highest expression of AL355711, ranked from highest (top) to lower CAGE RNA-seq expression (tags per million, tpm) values (chr21:43719104-43720919). 4

Supplementary Figure 2. FANTOM5 Consortium ZENBU Browser view of human tissues and cell types with the highest expression of BC039551 expressed in -and also relatively restricted to- placental tissue (chr4:153855668-153857989). Expression ranked from highest (top) to lower CAGE RNA-seq expression (tags per million, tpm). b. Log2 intensity vs GA comparison of BC039551 in maternal whole blood (probe#1 on left, and probe #2 on right). 4

Supplementary Figure 3. FANTOM5 Consortium ZENBU Browser view of human tissues and cell types with the highest expression of JHDM1D-AS1 ranked from highest (top) to lower CAGE RNA-seq expression (tags per million, tpm) values (chr7:139876984-139879440). 5

Supplementary Figure 4. FANTOM5 Consortium ZENBU Browser view of human tissues and cell types expression of A2M-AS1, ranked from highest (top) to lower CAGE RNA-seq expression (tags per million, tpm) values (chr12:9217773-9220651). 5

Supplementary Figure 5. FANTOM5 Consortium ZENBU Browser view of human tissues and cell types with the highest expression of NR_034004, ranked from highest (top) to lower CAGE RNA-seq expression (tags per million, tpm) values (chrX:70998019-71004228). 6

Supplementary Figure 6. FANTOM5 Consortium ZENBU Browser view of human tissues and cell types with the highest expression of LINC00649, ranked from highest (top) to lower CAGE RNA-seq expression (tags per million, tpm) values (chr21:35303516-35343487). 6

Supplementary Figure 7. *FANTOM5 Consortium ZENBU Browser view of human tissues and cell types with the highest expression of LINC00861, ranked from highest (top) to lower CAGE RNA-seq expression (tags per million, tpm) values (chr8:126953376-126963441).* 7

Supplementary Figure 8. FANTOM5 Consortium ZENBU Browser view of human tissues and cell types with the highest expression of LINC01094, ranked from highest (top) to lower CAGE RNA-seq expression (tags per million, tpm) values (chr4:79567148-79605655). 8

Supplementary Figure 9. FANTOM5 Consortium ZENBU Browser view of human tissues and cell types with the highest expression of MANEA-AS1, ranked from highest (top) to lower CAGE RNA-seq expression (tags per million, tpm) values (chr6:96023059-96025326). 9

Supplementary Figure 10. FANTOM5 Consortium ZENBU Browser view of human tissues and cell types with the highest expression of UBBP4, ranked from highest (top) to lower CAGE RNA-seq expression (tags per million, tpm) values (chr17:21729873-21731760). 9

Supplementary Figure 11. FANTOM5 Consortium ZENBU Browser view of human tissues and cell types with the highest expression of FOXO3B, ranked from highest (top) to lower CAGE RNA-seq expression (tags per million, tpm) values (chr17:18569236-18576494). 10

Supplementary Figure 12. FANTOM5 Consortium ZENBU Browser view of human tissues and cell types with the highest expression of MKRN9P, ranked from highest (top) to lower CAGE RNA-seq expression (tags per million, tpm) values (chr12:88176663-88178488). 10

Supplementary Figure 13. FANTOM5 Consortium ZENBU Browser view of human tissues and cell types with the highest expression of PSME2P2 ranked from highest (top) to lower CAGE RNA-seq expression (tags per million, tpm) values (chr13:49345232-49346006) 11

Supplementary Figure 14. FANTOM5 Consortium ZENBU Browser view of human tissues and cell types with the highest expression of YBX3P1 ranked from highest (top) to lower CAGE RNA-seq expression (tags per million, tpm) values (chr16:31579088-31580845) 11

Supplementary Figure 15. FANTOM5 Consortium ZENBU Browser view of human tissues and cell types with the highest expression of MIR4439 ranked from highest (top) to lower CAGE RNA-seq expression (tags per million, tpm) values (chr2:225875178-225875257). 12
Supplementary Figure 16. FANTOM5 Consortium ZENBU Browser view of human tissues and cell types with the highest expression of SNORD41 ranked from highest (top) to lower CAGE RNA-seq expression (tags per million, tpm) values (chr19:12817263-12817332). 12
Supplementary Figure 17. FANTOM5 Consortium ZENBU Browser view of human tissues and cell types with the highest expression of SCARNA2 ranked from highest (top) to lower CAGE RNA-seq expression (tags per million, tpm) values (chr1:109642815-109643234). 13
Supplementary Figure 18. Trend in expression overtime with GA for 3 regulatory non-coding RNAs differentially expressed during gestation: MIR4439 (regulatory ncRNA) chr2:225875178-225875257, SNORD41 (regulatory ncRNA) chr19:12817263-12817332, and SCARNA2 (ncRNA) chr1:109642815-109643234, also shown above. 14
Supplementary Figure 19. GTEx for MIR4439 ranked from highest (top) to lower RNA-seq expression data (tags per million, tpm) values. 15
Supplementary Figure 20. GTEx for SNORD41 ranked from highest (top) to lower RNA-seq expression data (tags per million, tpm) values. 15
Supplementary Figure 21. GTEx for SCARNA2 ranked from highest (top) to lower RNA-seq expression data (tags per million, tpm) values. 16
Supplementary Figure 22. GTEx Portal view of human tissues and cell types with the highest expression of PSME2P2 ranked from highest (top) to lower RNA-seq expression data (tags per million, tpm) values. 16
Supplementary Figure 23. GTEx Portal view of human tissues and cell types with the highest expression of MANEA-AS1 ranked from highest (top) to lower RNA-seq expression data (tags per million, tpm) values. 16
Supplementary Figure 24. GTEx Portal view of human tissues and cell types with the highest expression of UBBP4 ranked from highest (top) to lower RNA-seq expression data (tags per million, tpm) values. 17
Supplementary Figure 25. GTEx Portal view of human tissues and cell types with the highest expression of FOXO3B ranked from highest (top) to lower RNA-seq expression data (tags per million, tpm) values. 17
Supplementary Figure 26. GTEx Portal view of human tissues and cell types with the highest expression of MKRN9P ranked from highest (top) to lower RNA-seq expression data (tags per million, tpm) values. 18
Supplementary Figure 27. GTEx Portal view of human tissues and cell types with the highest expression of RP11-535M15.2 ranked from highest (top) to lower RNA-seq expression data (tags per million, tpm) values. 18
Supplementary Figure 28. GTEx Portal view of human tissues and cell types with the highest expression of CSDAP1 ranked from highest (top) to lower RNA-seq expression data (tags per million, tpm) values. 19
Supplementary Figure 29. GTEx Portal view of human tissues and cell types with the highest expression of LINC00861ranked from highest (top) to lower RNA-seq expression data (tags per million, tpm) values. 19

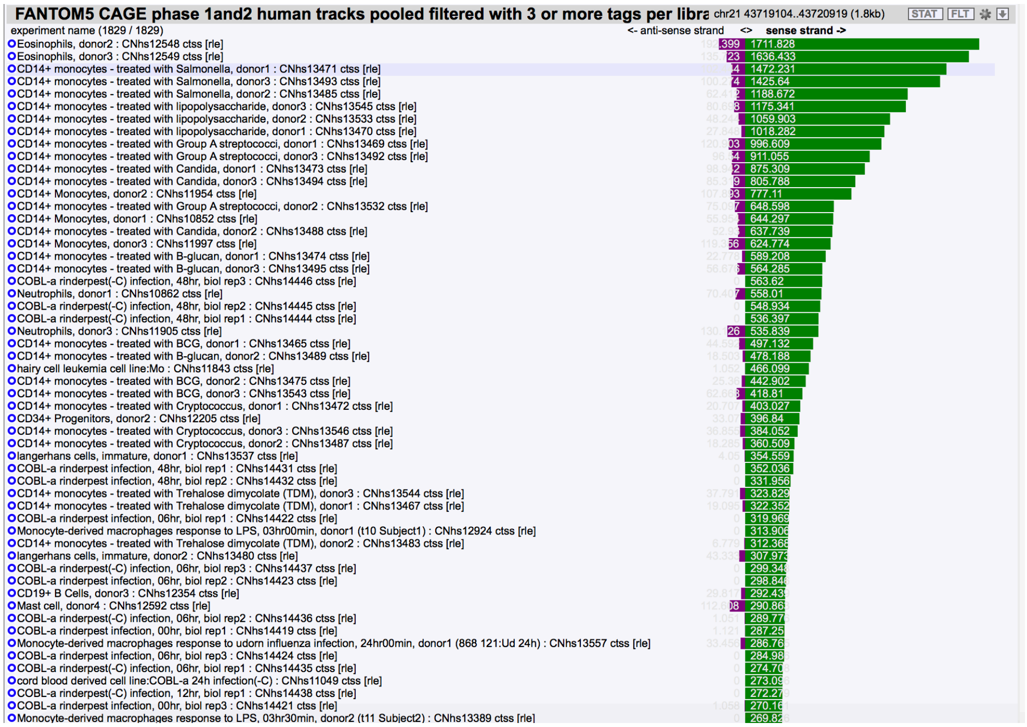

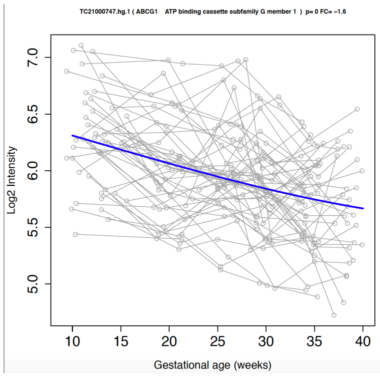

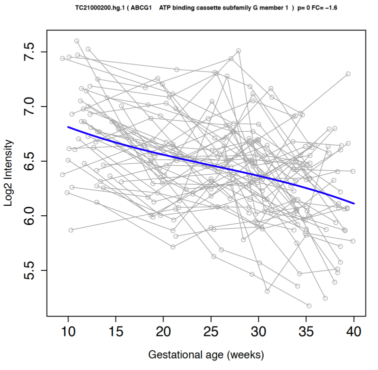

Supplementary Figure 1*. FANTOM5 Consortium ZENBU Browser view of human tissues and cell types with the highest expression of AL355711, ranked from highest (top) to lower CAGE RNA-seq expression (tags per million, tpm) values (chr21:43719104-43720919).*

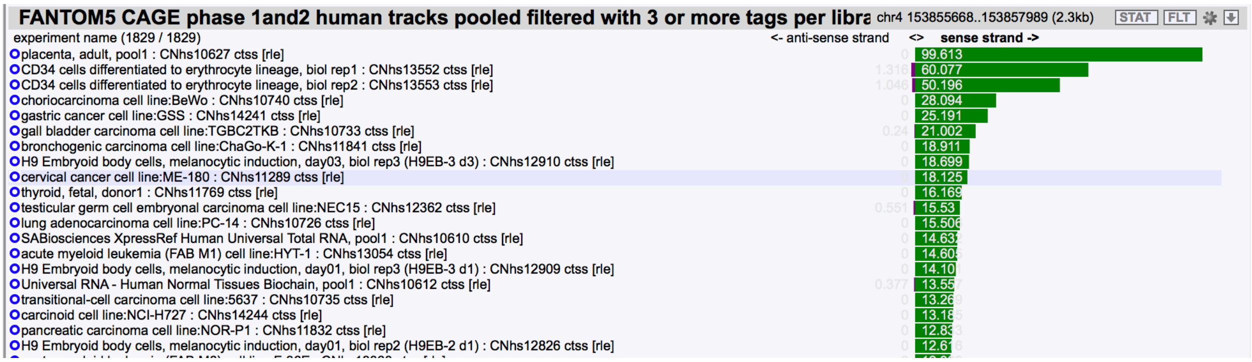

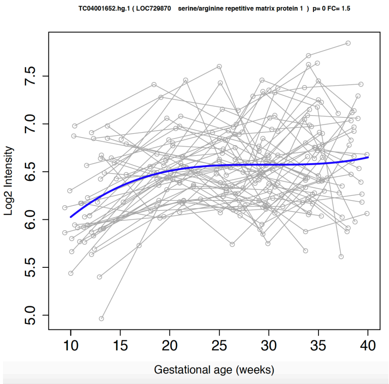

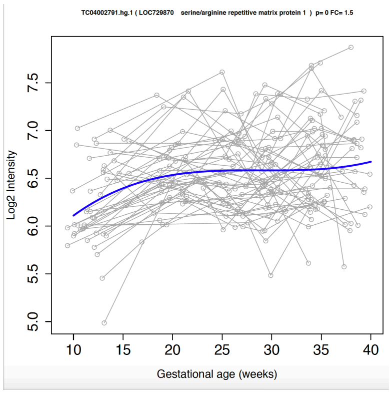

Supplementary Figure 2*. FANTOM5 Consortium ZENBU Browser view of human tissues and cell types with the highest expression of BC039551 expressed in -and also relatively restricted to- placental tissue (chr4:153855668-153857989). Expression ranked from highest (top) to lower CAGE RNA-seq expression (tags per million, tpm). b. Log2 intensity vs GA comparison of BC039551 in maternal whole blood (probe#1 on left, and probe #2 on right).*

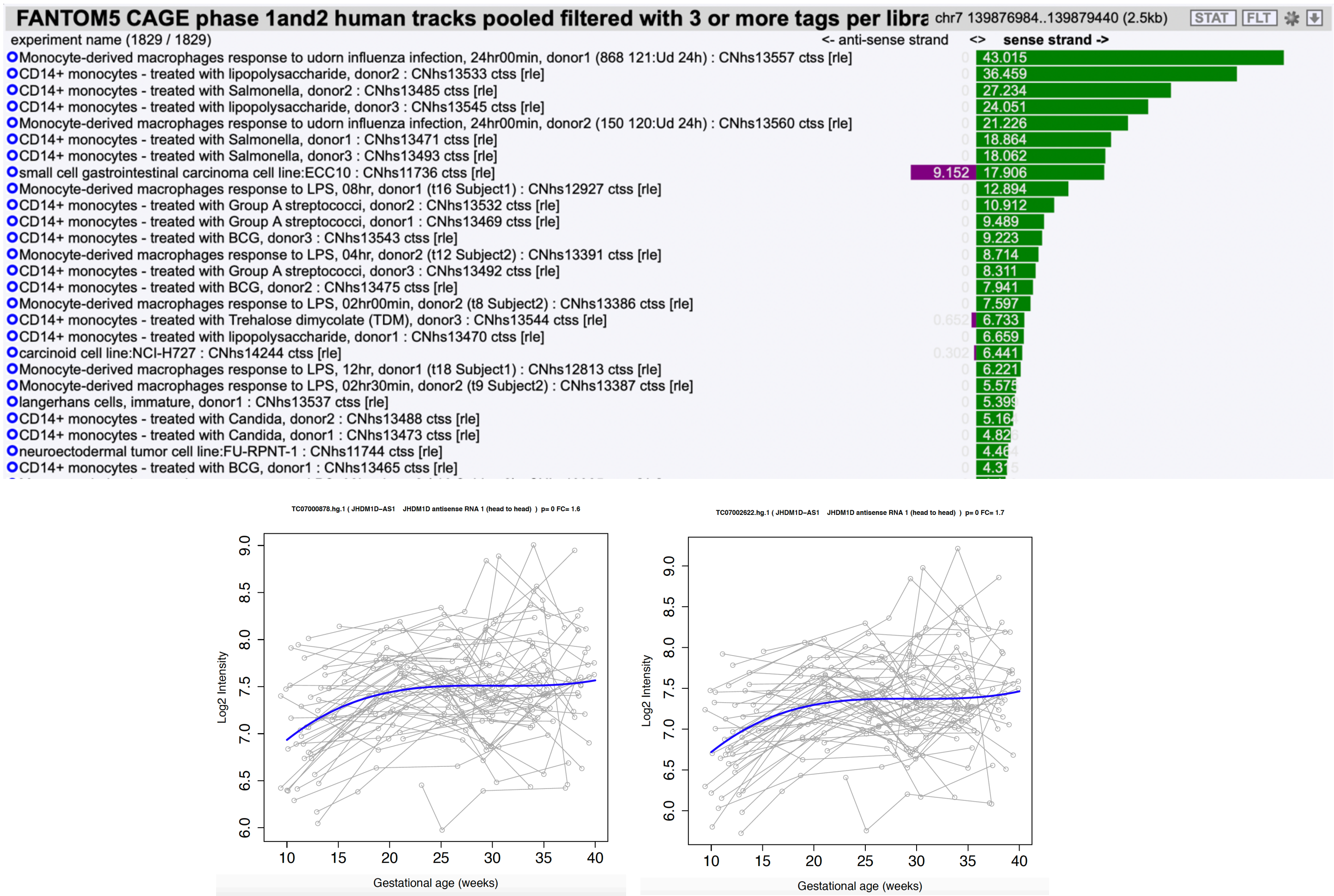

Supplementary Figure 3. *FANTOM5 Consortium ZENBU Browser view of human tissues and cell types with the highest expression of JHDM1D-AS1 ranked from highest (top) to lower CAGE RNA-seq expression (tags per million, tpm) values (chr7:139876984-139879440).*

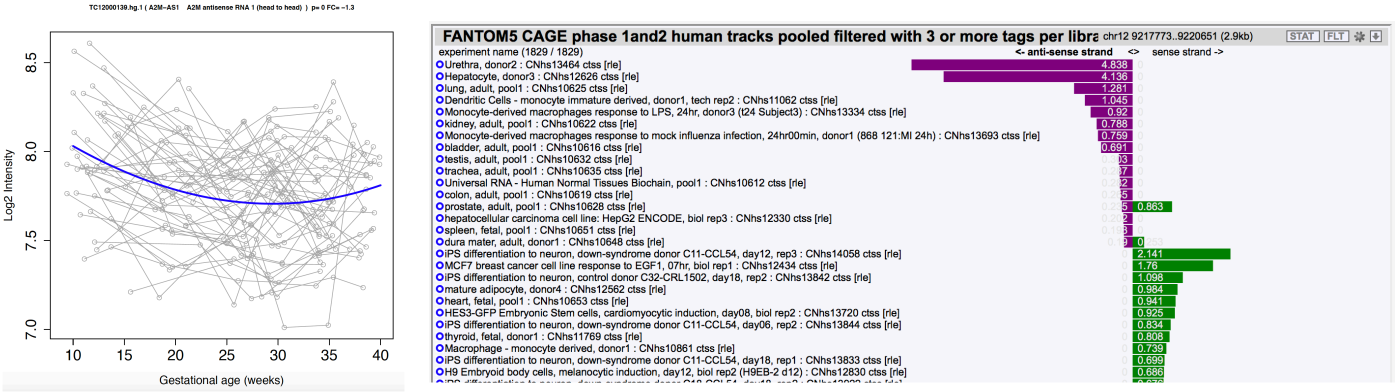

Supplementary Figure 4. *FANTOM5 Consortium ZENBU Browser view of human tissues and cell types expression of A2M-AS1, ranked from highest (top) to lower CAGE RNA-seq expression (tags per million, tpm) values (chr12:9217773-9220651).*

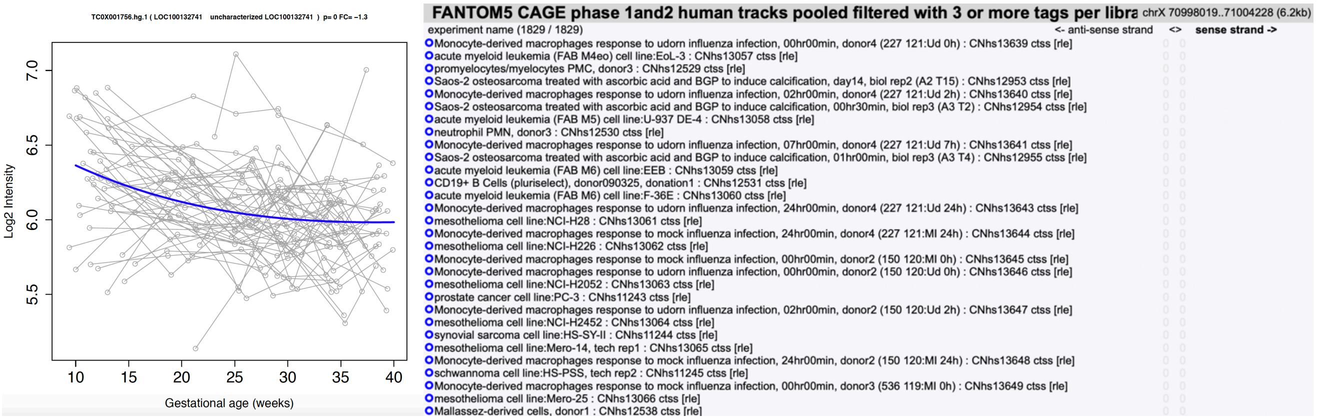

Supplementary Figure 5. *FANTOM5 Consortium ZENBU Browser view of human tissues and cell types with the highest expression of NR_034004, ranked from highest (top) to lower CAGE RNA-seq expression (tags per million, tpm) values (chrX:70998019-71004228).*

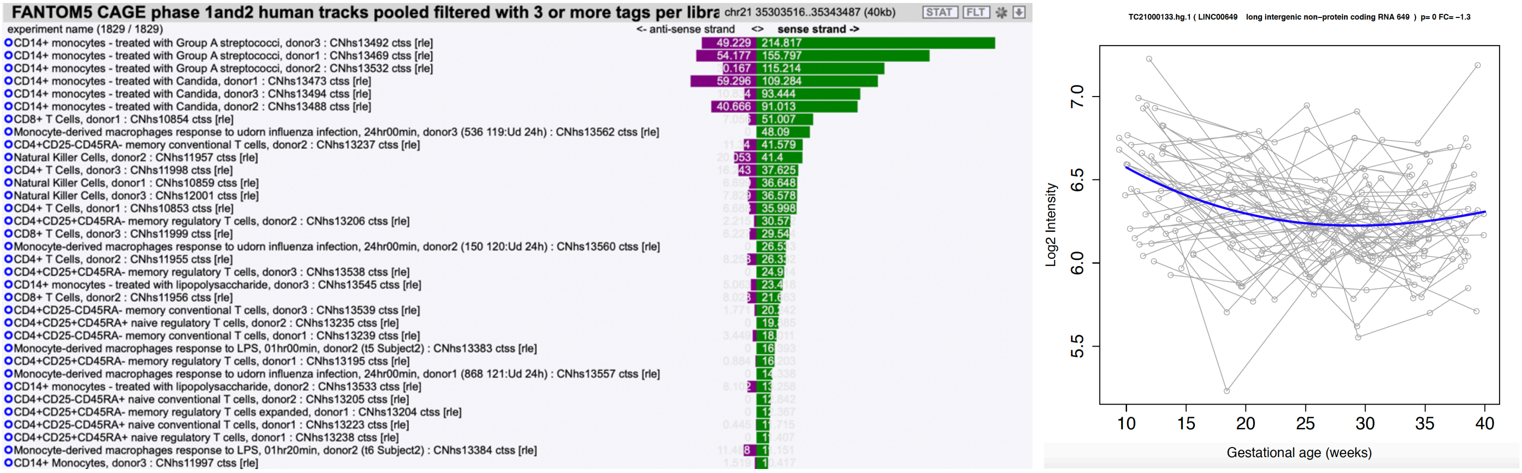

Supplementary Figure 6. *FANTOM5 Consortium ZENBU Browser view of human tissues and cell types with the highest expression of LINC00649, ranked from highest (top) to lower CAGE RNA-seq expression (tags per million, tpm) values (chr21:35303516-35343487).*

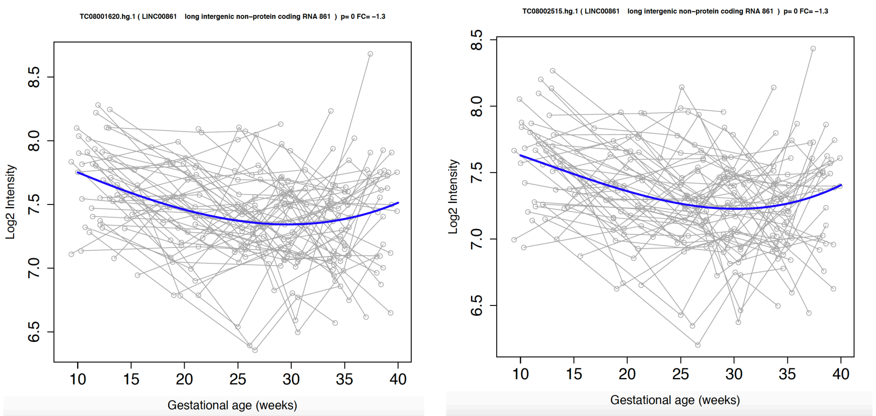

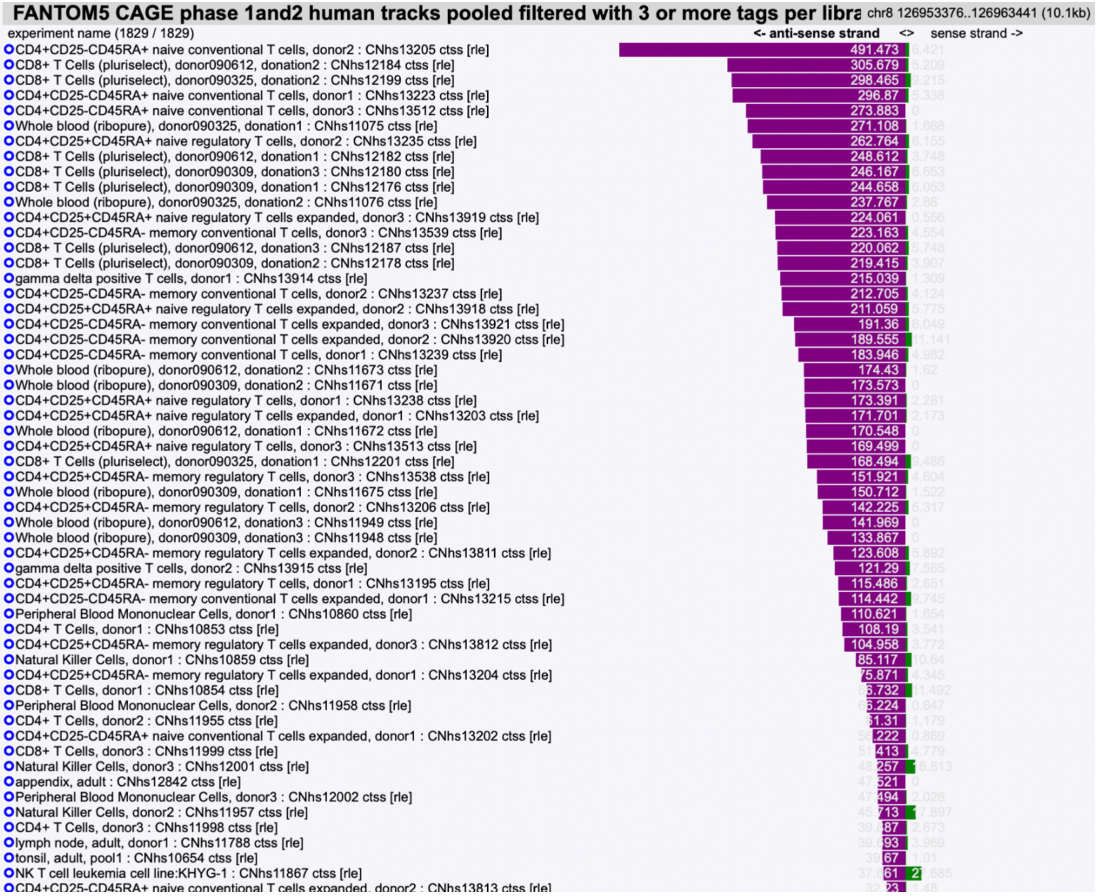

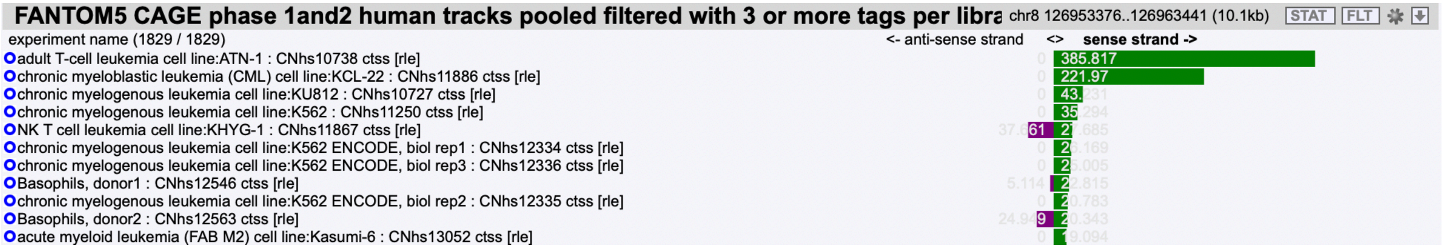

Supplementary Figure 7. *FANTOM5 Consortium ZENBU Browser view of human tissues and cell types with the highest expression of LINC00861, ranked from highest (top) to lower CAGE RNA-seq expression (tags per million, tpm) values (chr8:126953376-126963441).*

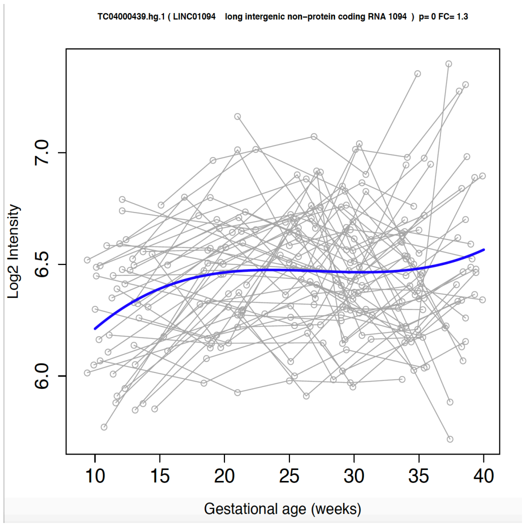

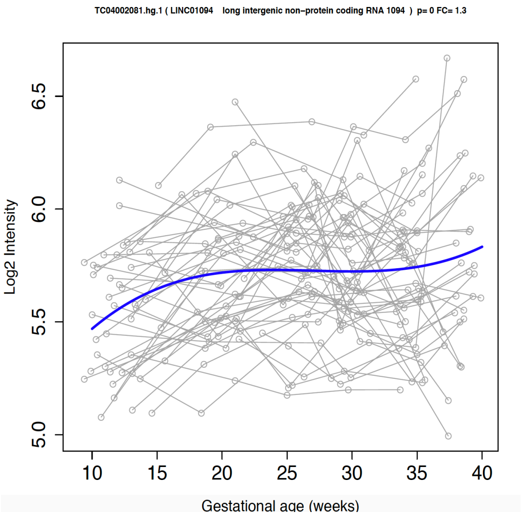

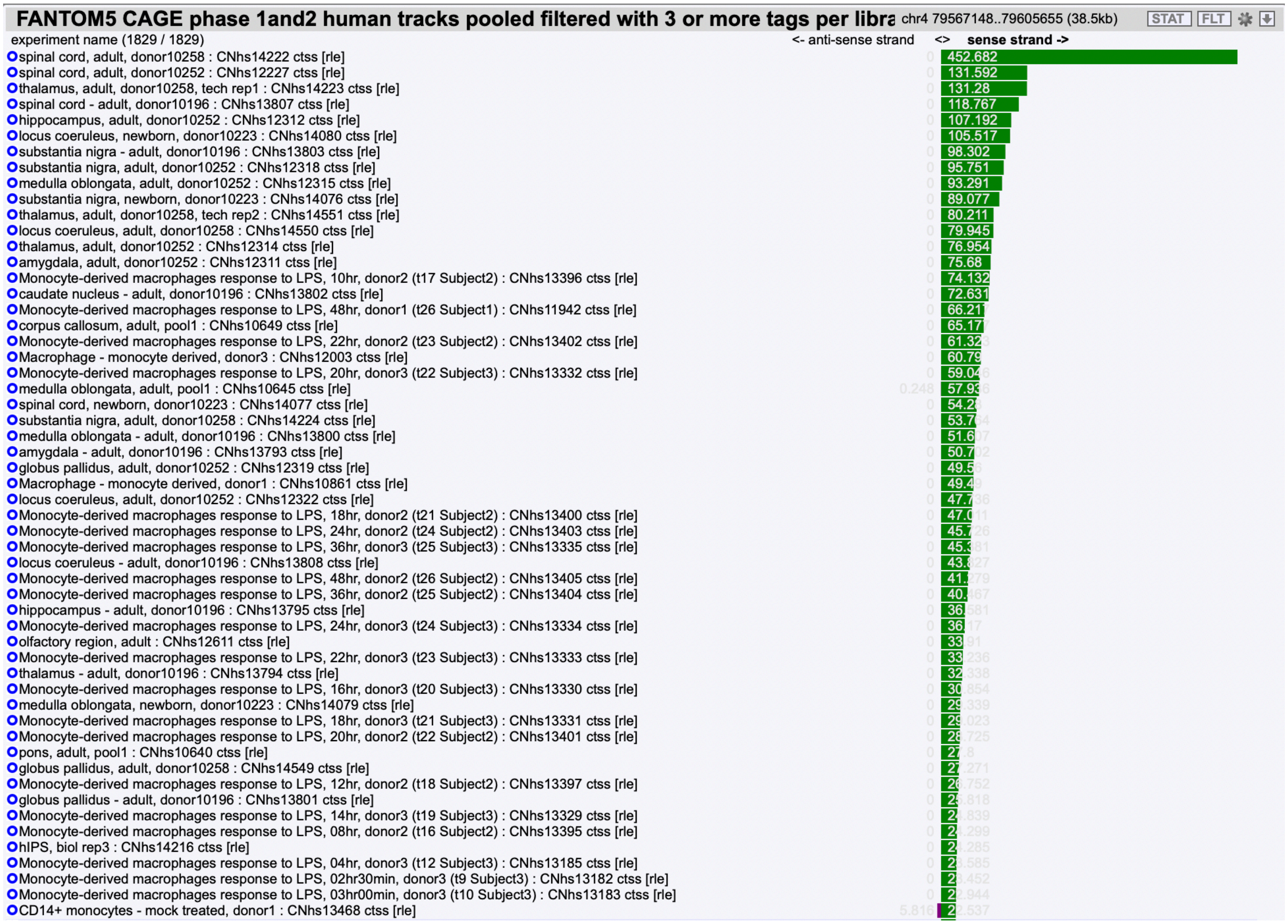

Supplementary Figure 8. *FANTOM5 Consortium ZENBU Browser view of human tissues and cell types with the highest expression of LINC01094, ranked from highest (top) to lower CAGE RNA-seq expression (tags per million, tpm) values (chr4:79567148-79605655).*

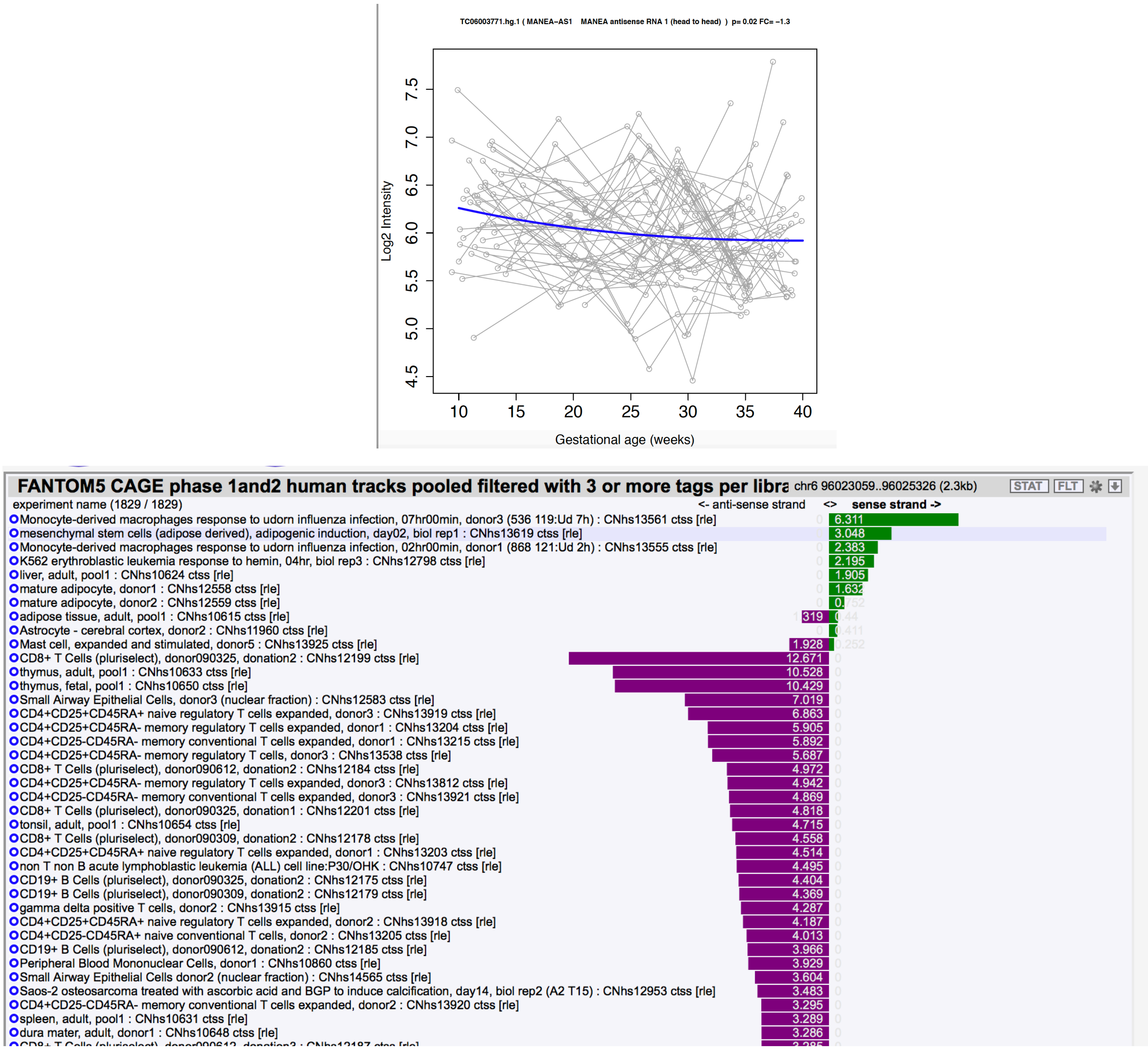

Supplementary Figure 9. *FANTOM5 Consortium ZENBU Browser view of human tissues and cell types with the highest expression of MANEA-AS1, ranked from highest (top) to lower CAGE RNA-seq expression (tags per million, tpm) values (chr6:96023059-96025326).*

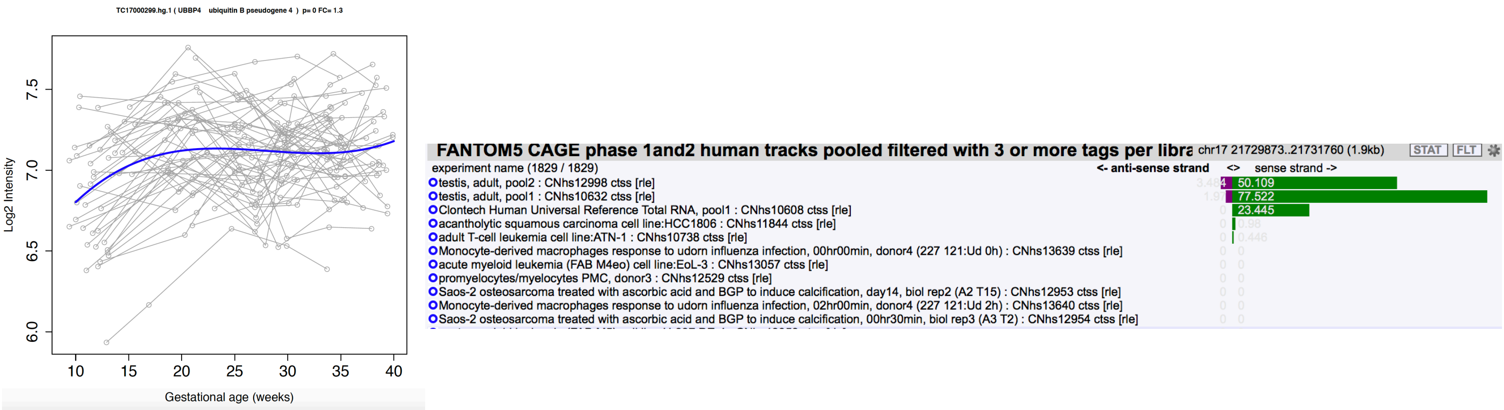

Supplementary Figure 10. *FANTOM5 Consortium ZENBU Browser view of human tissues and cell types with the highest expression of UBBP4, ranked from highest (top) to lower CAGE RNA-seq expression (tags per million, tpm) values (chr17:21729873-21731760).*

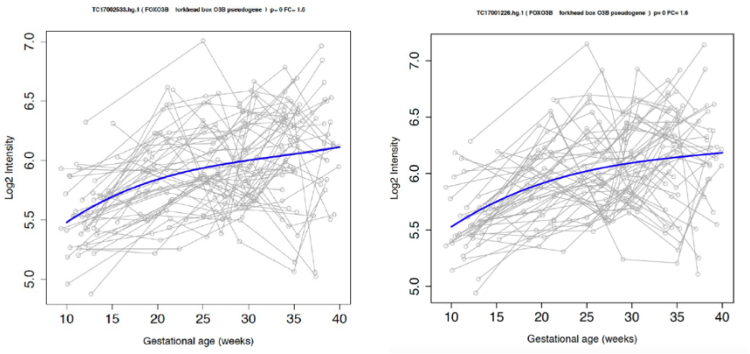

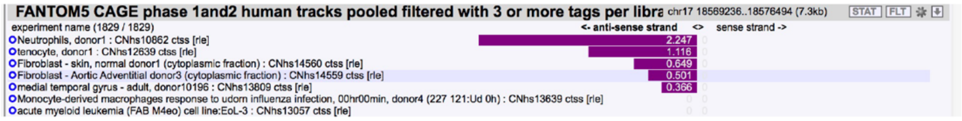

Supplementary Figure 11. *FANTOM5 Consortium ZENBU Browser view of human tissues and cell types with the highest expression of FOXO3B, ranked from highest (top) to lower CAGE RNA-seq expression (tags per million, tpm) values (chr17:18569236-18576494).*

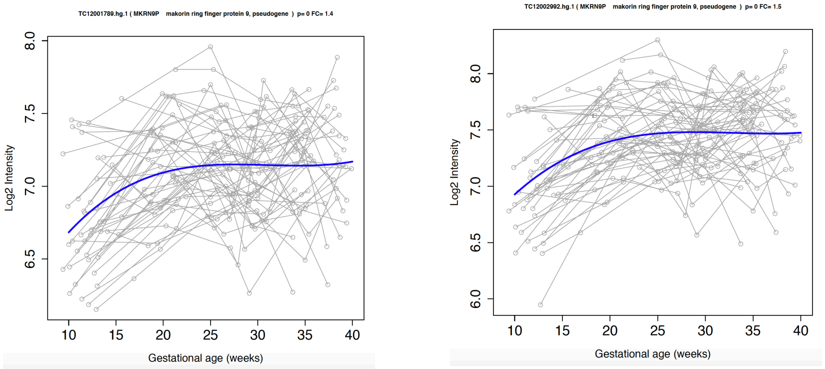

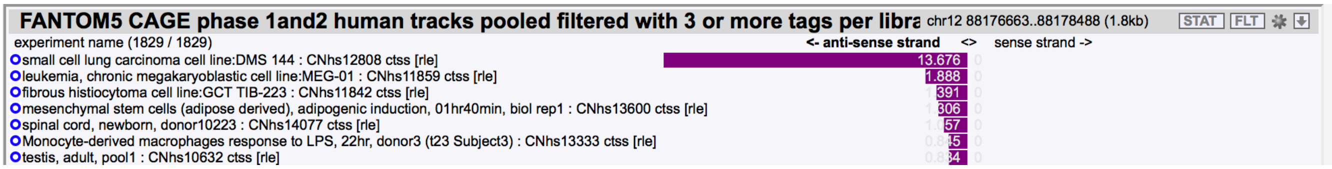

Supplementary Figure 12. *FANTOM5 Consortium ZENBU Browser view of human tissues and cell types with the highest expression of MKRN9P, ranked from highest (top) to lower CAGE RNA-seq expression (tags per million, tpm) values (chr12:88176663-88178488).*

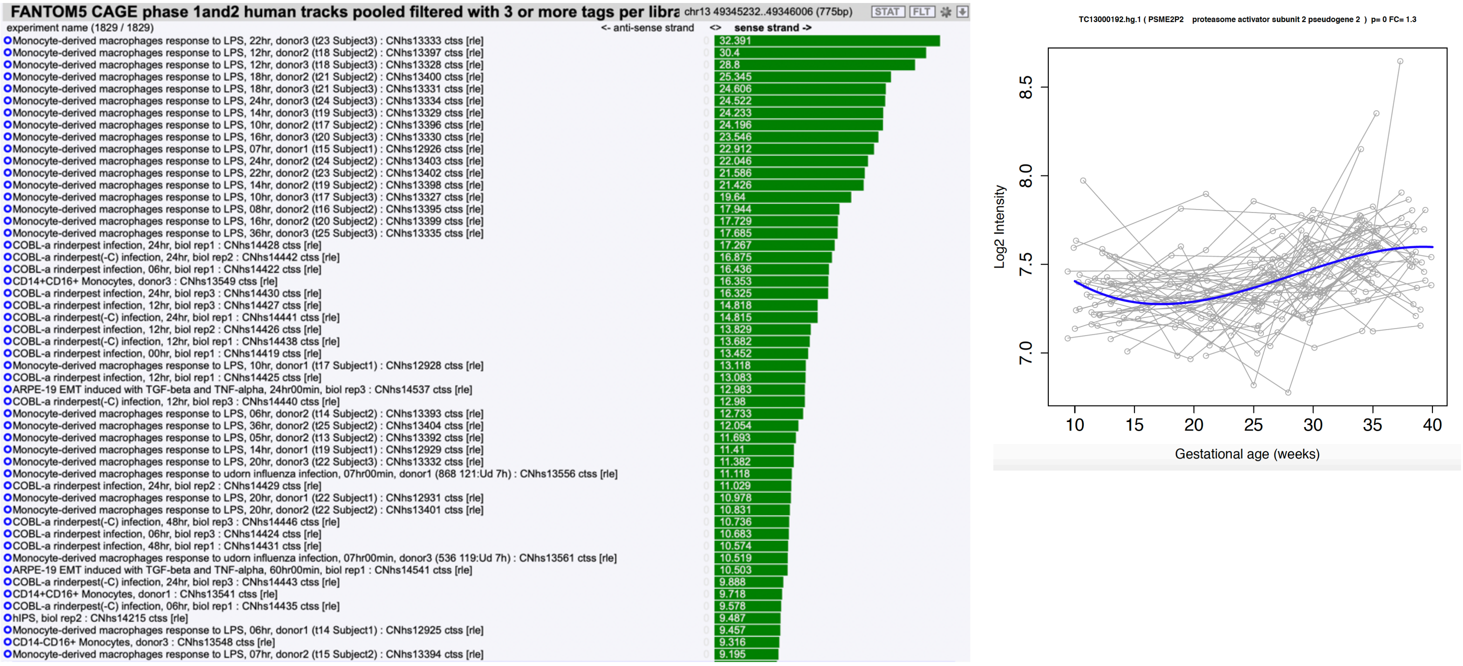

Supplementary Figure 13. *FANTOM5 Consortium ZENBU Browser view of human tissues and cell types with the highest expression of PSME2P2 ranked from highest (top) to lower CAGE RNA-seq expression (tags per million, tpm) values (chr13:49345232-49346006)*

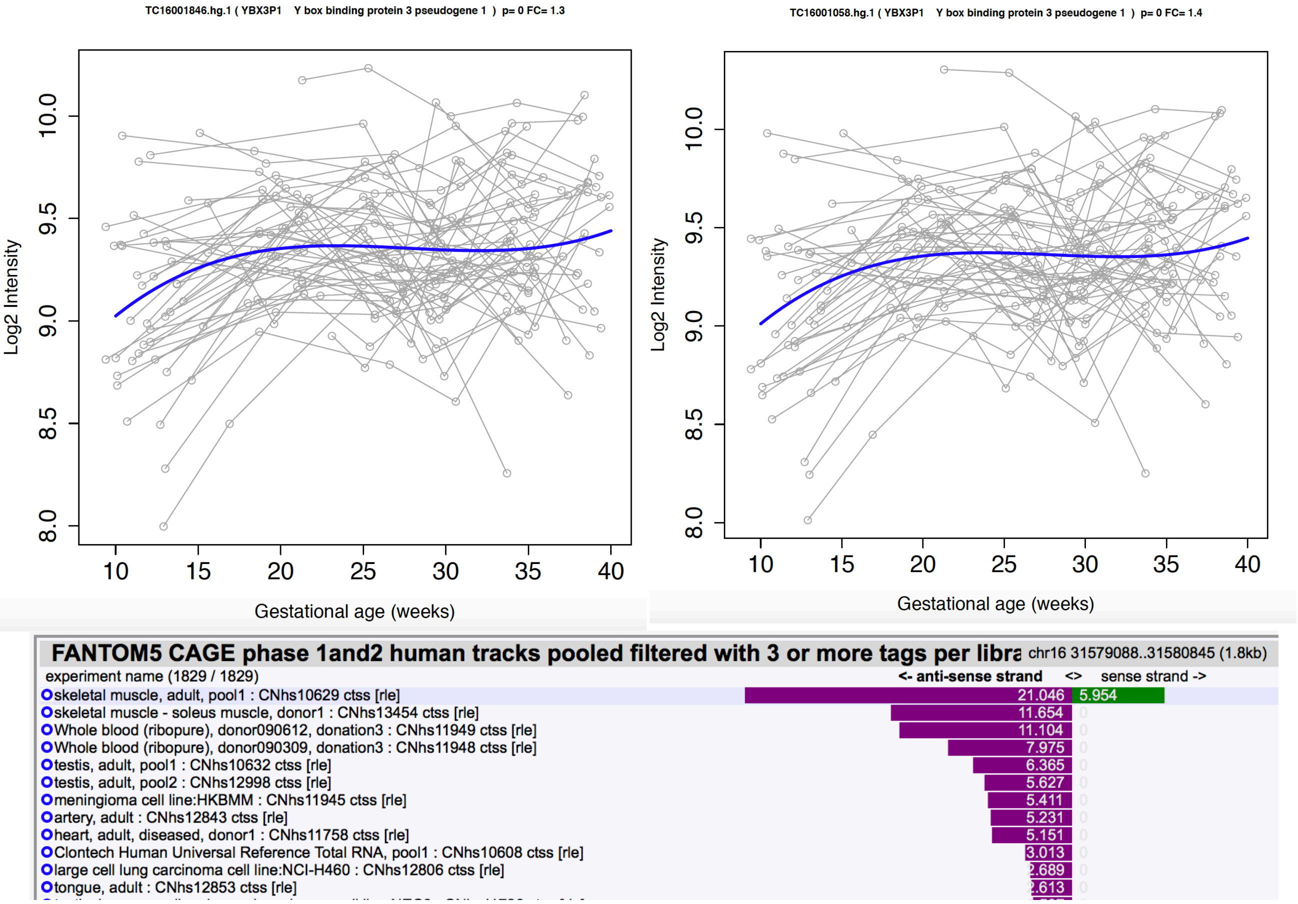

Supplementary Figure 14. *FANTOM5 Consortium ZENBU Browser view of human tissues and cell types with the highest expression of YBX3P1 ranked from highest (top) to lower CAGE RNA-seq expression (tags per million, tpm) values (chr16:31579088-31580845)*

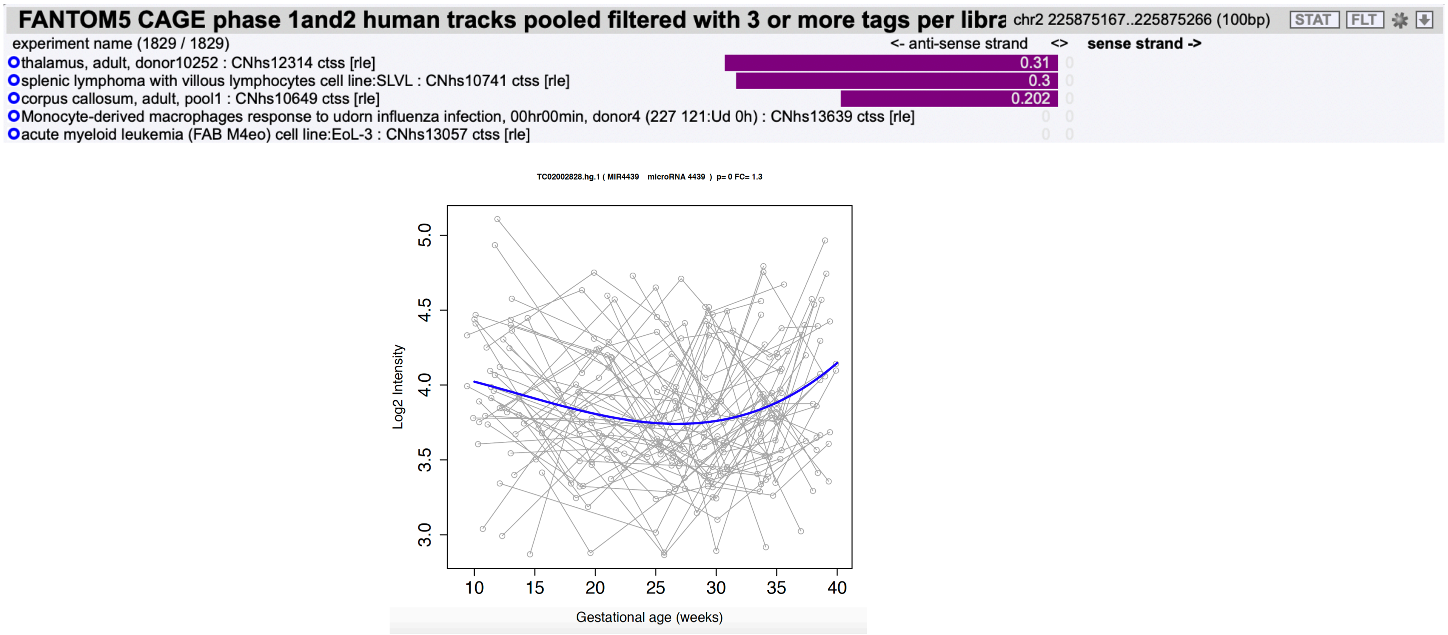

Supplementary Figure 15. *FANTOM5 Consortium ZENBU Browser view of human tissues and cell types with the highest expression of MIR4439 ranked from highest (top) to lower CAGE RNA-seq expression (tags per million, tpm) values (chr2:225875178-225875257).*

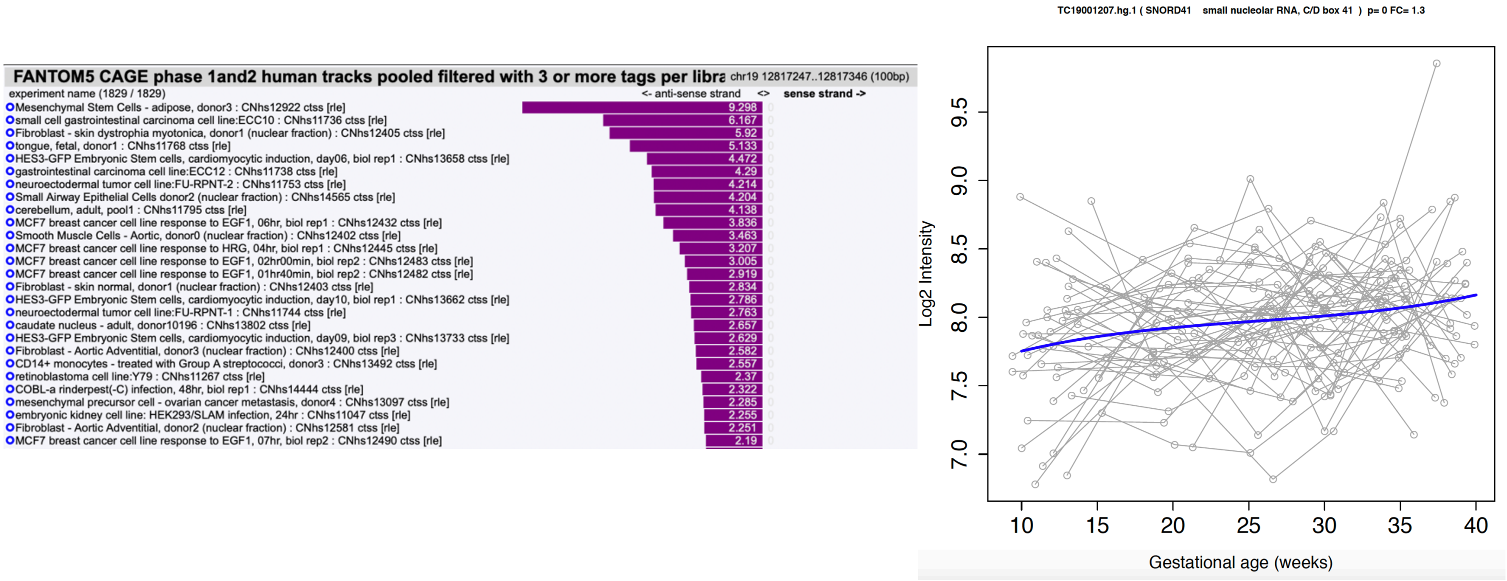

Supplementary Figure 16*. FANTOM5 Consortium ZENBU Browser view of human tissues and cell types with the highest expression of SNORD41 ranked from highest (top) to lower CAGE RNA-seq expression (tags per million, tpm) values (chr19:12817263-12817332).*

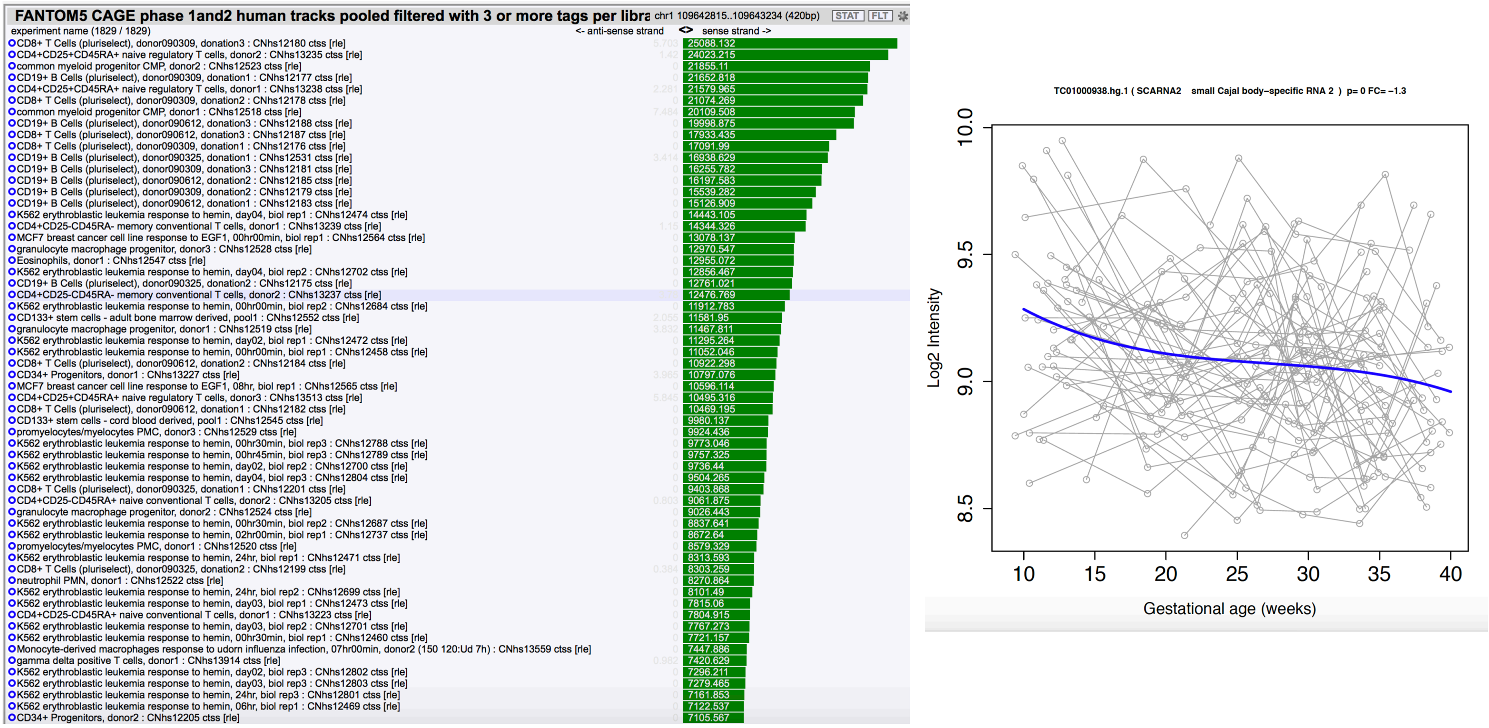

Supplementary Figure 17. *FANTOM5 Consortium ZENBU Browser view of human tissues and cell types with the highest expression of SCARNA2 ranked from highest (top) to lower CAGE RNA-seq expression (tags per million, tpm) values (chr1:109642815-109643234).*

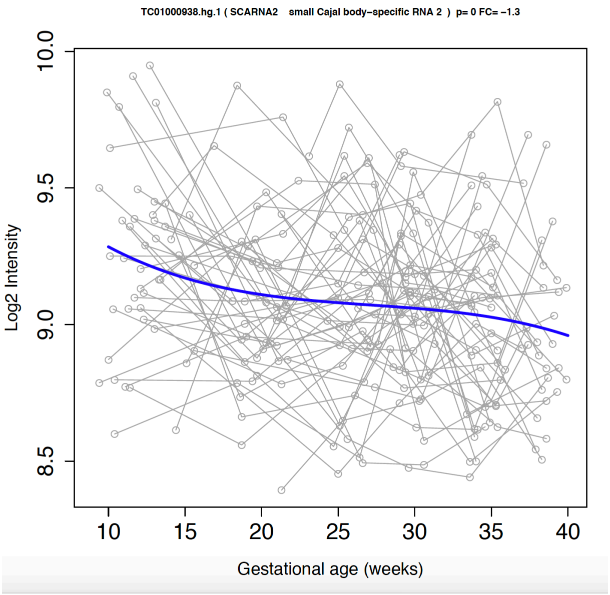

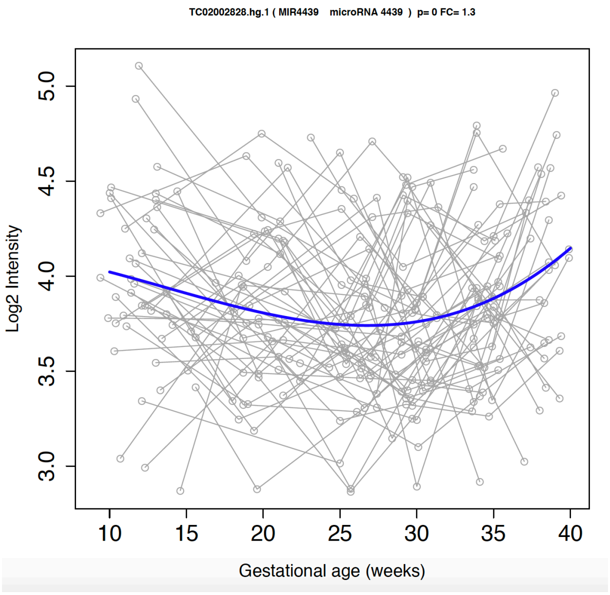

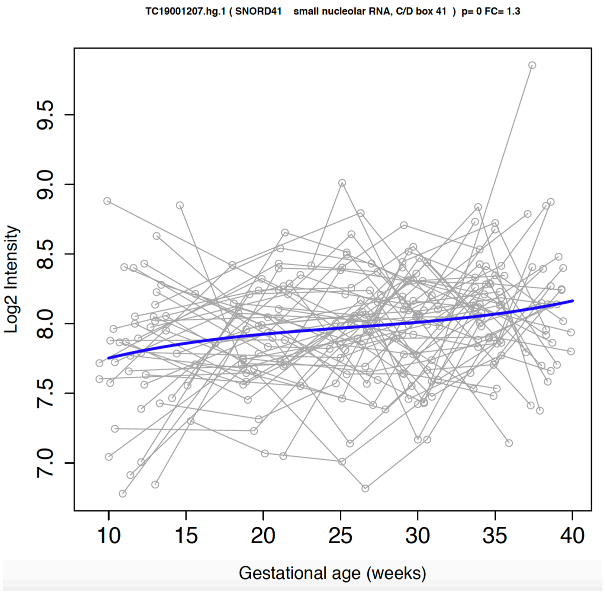

***Supplementary Figure 18**. Trend in expression overtime with GA for 3 regulatory non-coding RNAs differentially expressed during gestation: MIR4439 (regulatory ncRNA) chr2:225875178-225875257, SNORD41 (regulatory ncRNA) chr19:12817263-12817332, and SCARNA2 (ncRNA) chr1:109642815-109643234, also shown above.*

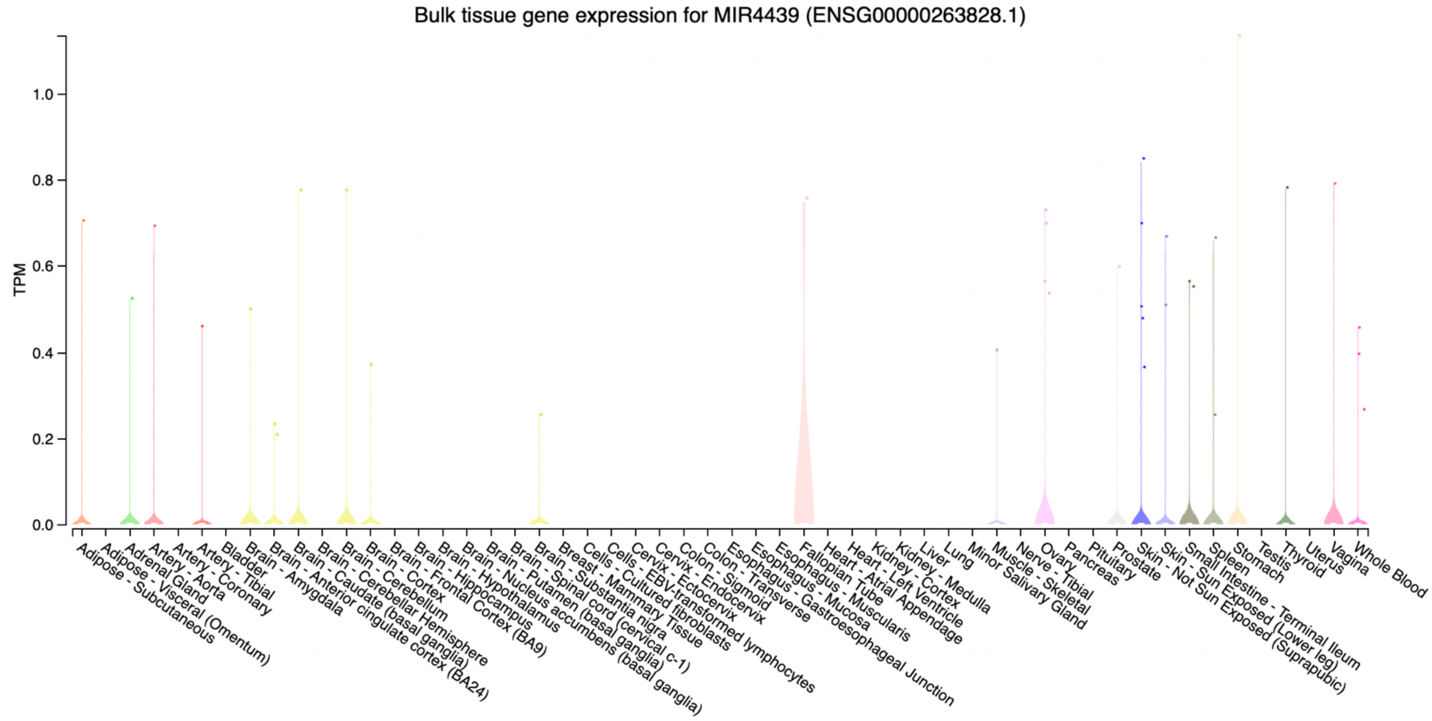

Supplementary Figure 19. *GTEx for MIR4439 ranked from highest (top) to lower RNA-seq expression data (tags per million, tpm) values.*

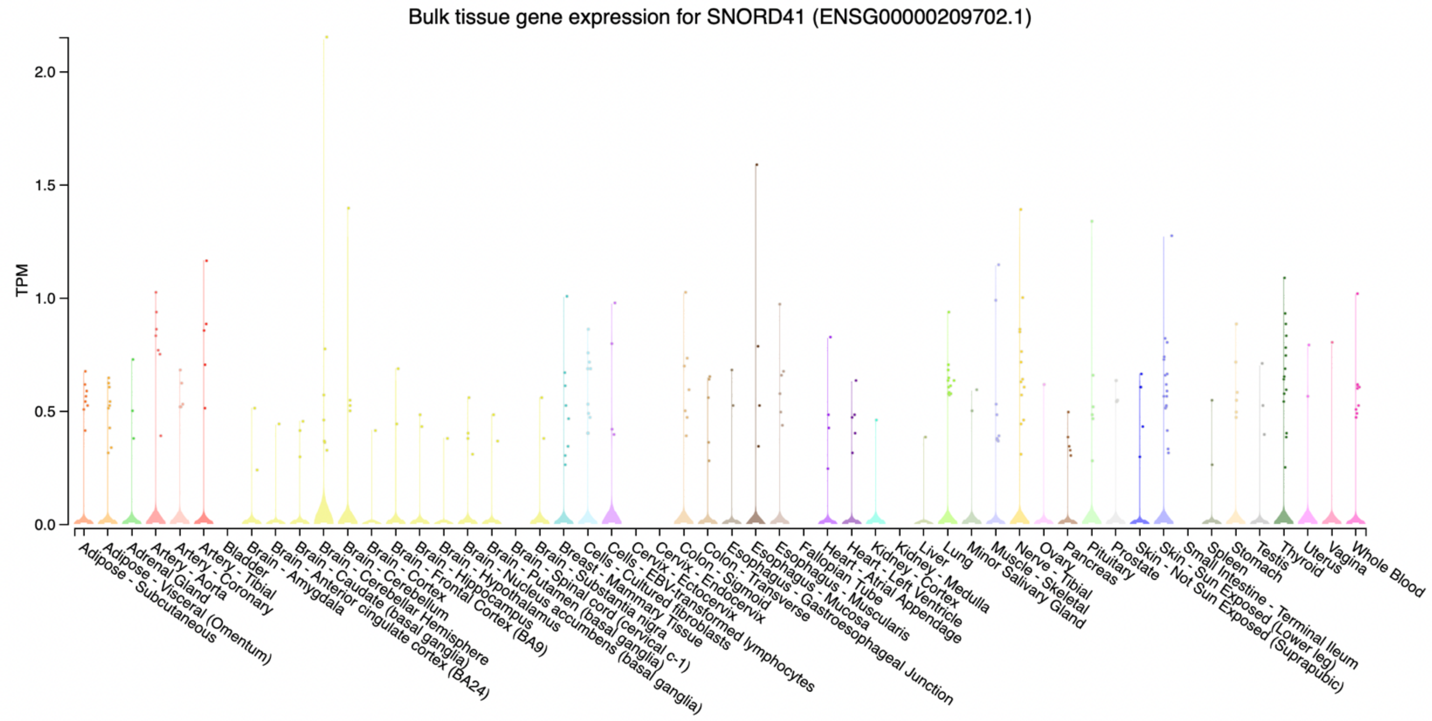

Supplementary Figure 20. *GTEx for SNORD41 ranked from highest (top) to lower RNA-seq expression data (tags per million, tpm) values.*

Supplementary Figure 21*. No bulk tissue expression was reported in GTEx data* for *SCARNA2* at the time of publication.

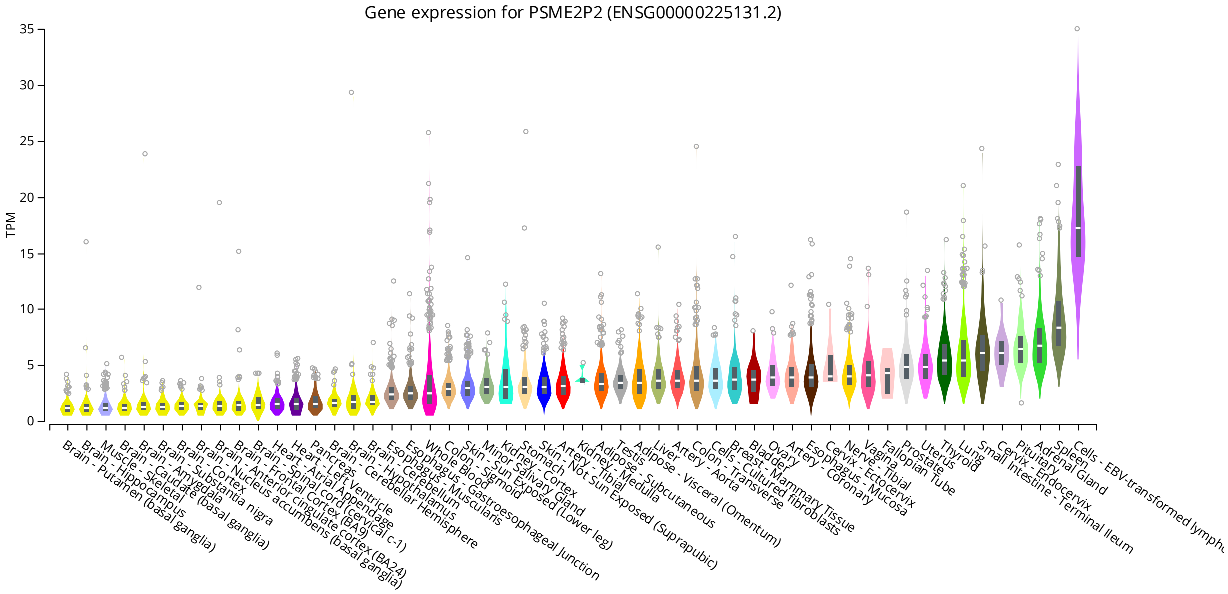

Supplementary Figure 22. *GTEx Portal view of human tissues and cell types with the highest expression of PSME2P2 ranked from highest (top) to lower RNA-seq expression data (tags per million, tpm) values.*

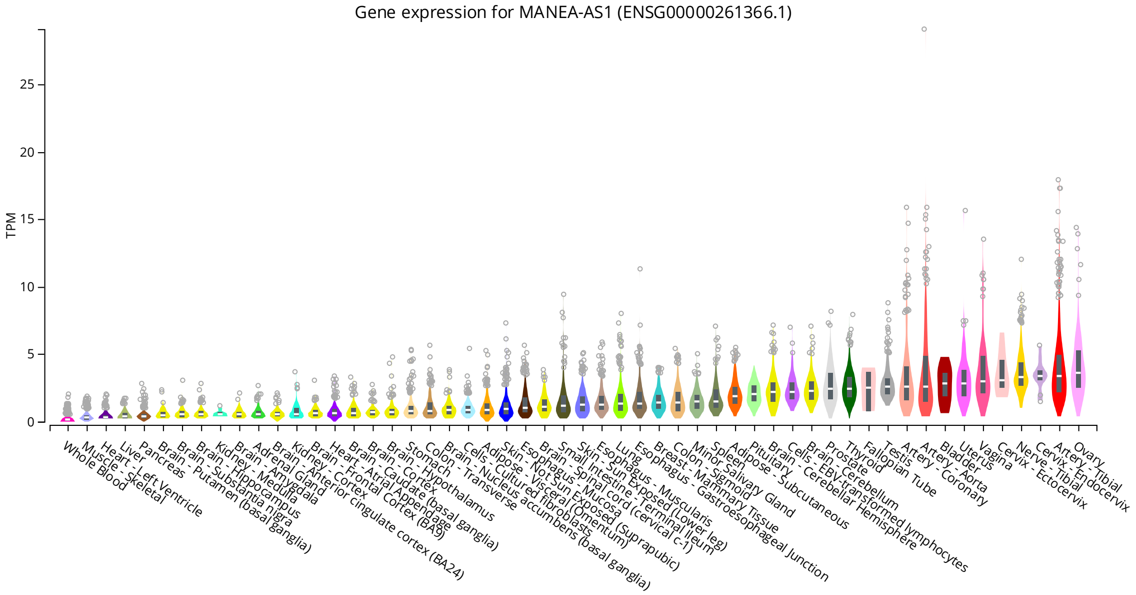

Supplementary Figure 23. *GTEx Portal view of human tissues and cell types with the highest expression of MANEA-AS1 ranked from highest (top) to lower RNA-seq expression data (tags per million, tpm) values.*

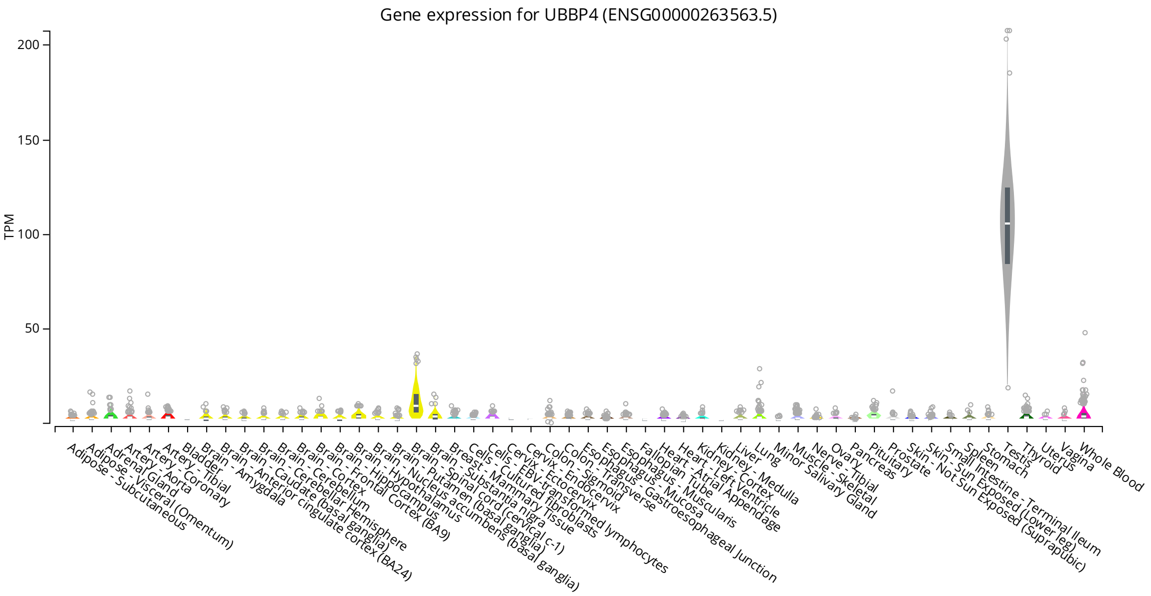

Supplementary Figure 24. *GTEx Portal view of human tissues and cell types with the highest expression of UBBP4 ranked from highest (top) to lower RNA-seq expression data (tags per million, tpm) values.*

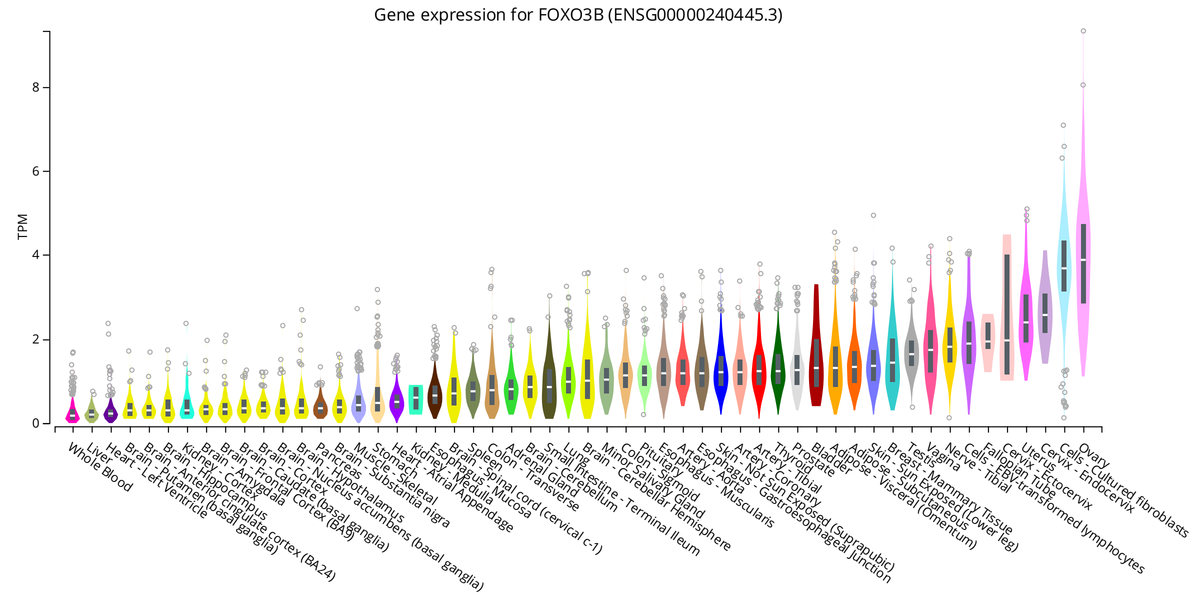

Supplementary Figure 25. *GTEx Portal view of human tissues and cell types with the highest expression of FOXO3B ranked from highest (top) to lower RNA-seq expression data (tags per million, tpm) values.*

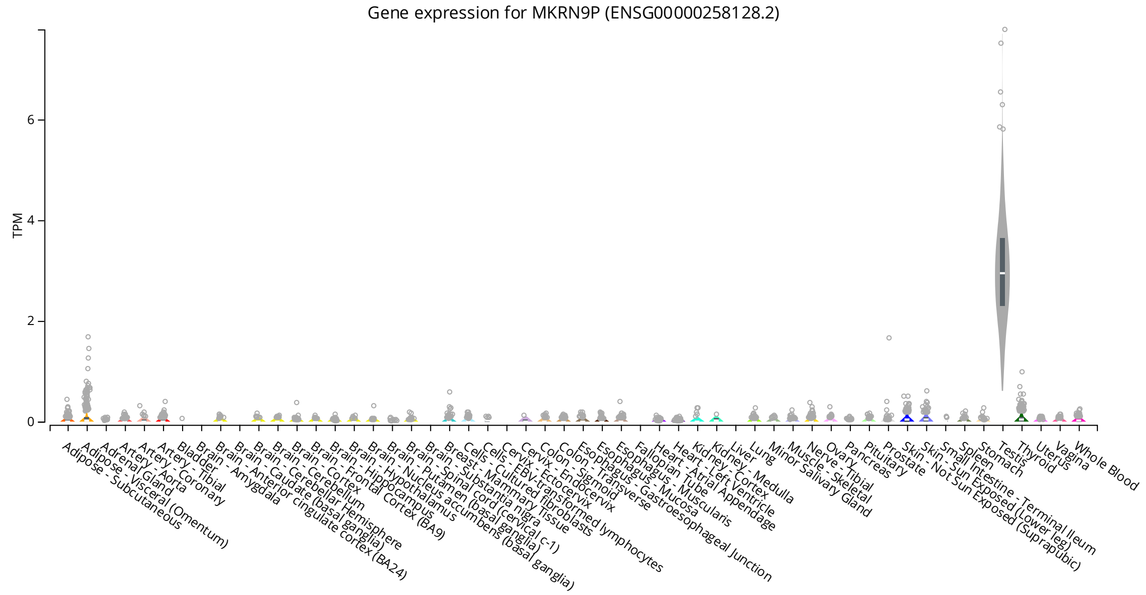

Supplementary Figure 26. *GTEx Portal view of human tissues and cell types with the highest expression of MKRN9P ranked from highest (top) to lower RNA-seq expression data (tags per million, tpm) values.*

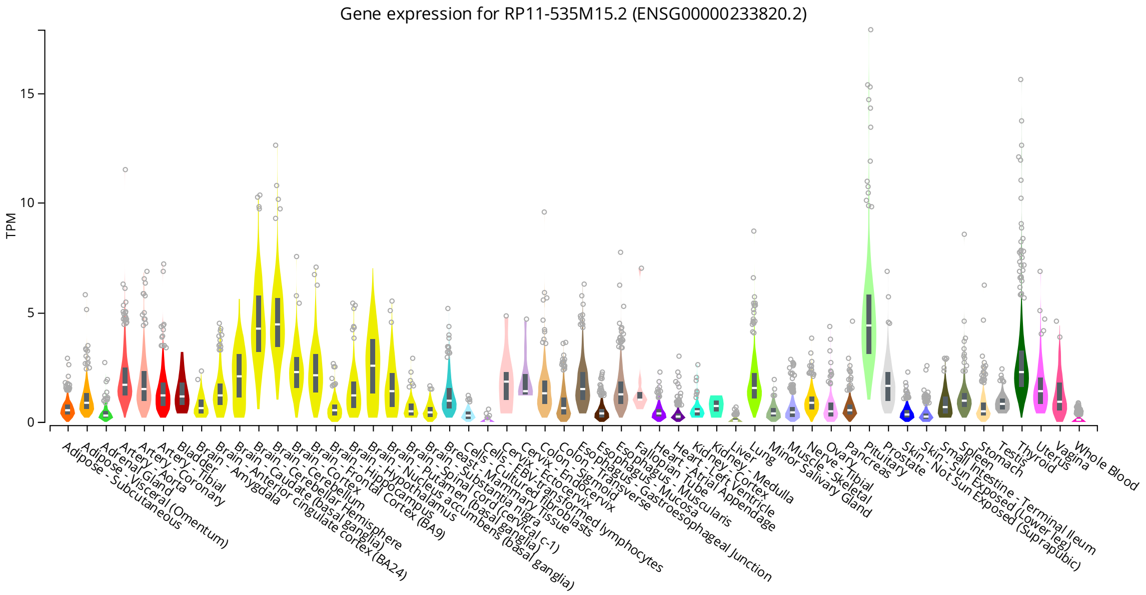

Supplementary Figure 27*. GTEx Portal view of human tissues and cell types with the highest expression of RP11-535M15.2 ranked from highest (top) to lower RNA-seq expression data (tags per million, tpm) values.*

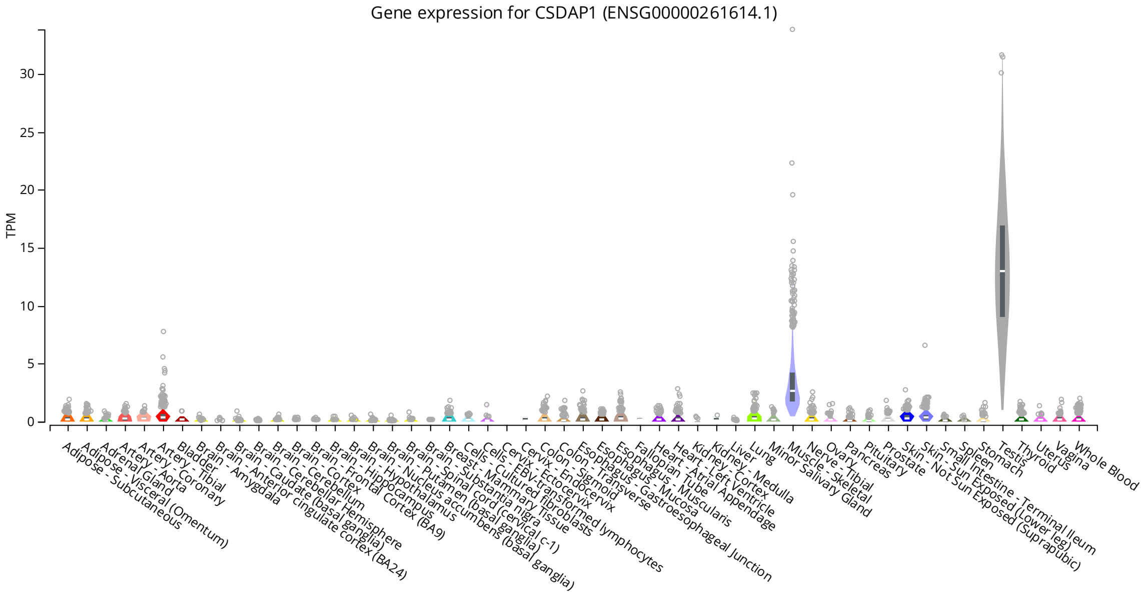

***Supplementary Figure 28**. GTEx Portal view of human tissues and cell types with the highest expression of CSDAP1 ranked from highest (top) to lower RNA-seq expression data (tags per million, tpm) values.*

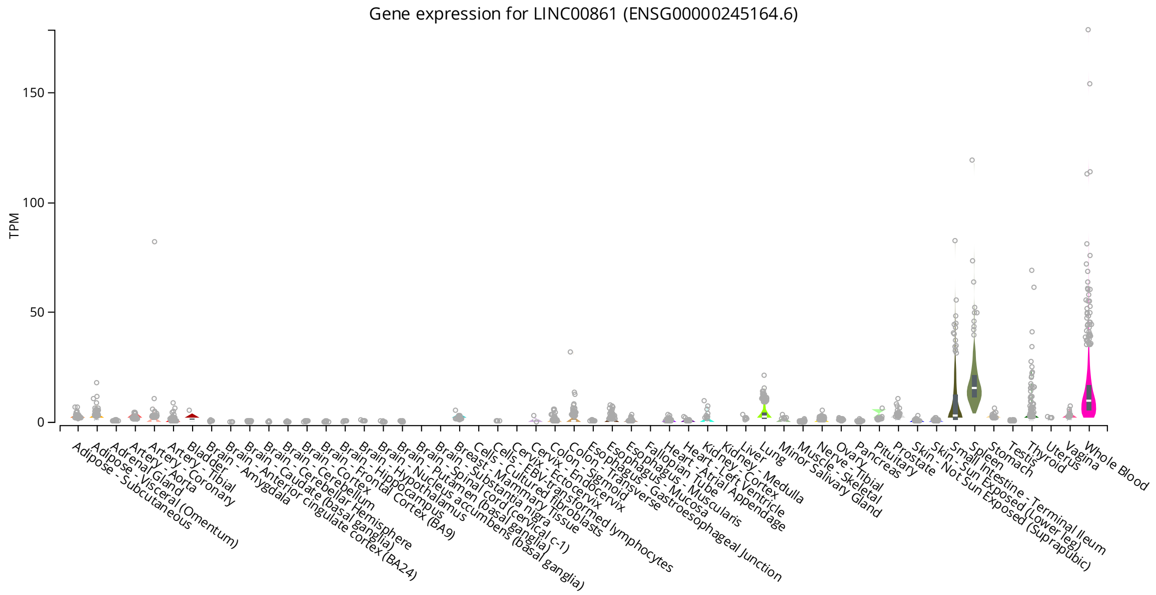

***Supplementary Figure 29.** GTEx Portal view of human tissues and cell types with the highest expression of LINC00861ranked from highest (top) to lower RNA-seq expression data (tags per million, tpm) values.*

Supplementary Figure 30: UCSC Genome Browser Profiles of Long non-coding RNAs (lncRNAs) and long intergenic non-coding RNAs (lincRNAs) differentially expressed over the GA timecourse of normal pregnancy.

Supplementary Figure 30. A. DNAseI Hypersensitivity Sites (display mode, dense), Transcription Factor ChIP Seq (ENCODE) Binding Sites (display mode, dense), and Epigenetic Signatures (display modes, full) of AL355711, BC039551, and JHDM1D-AS1.

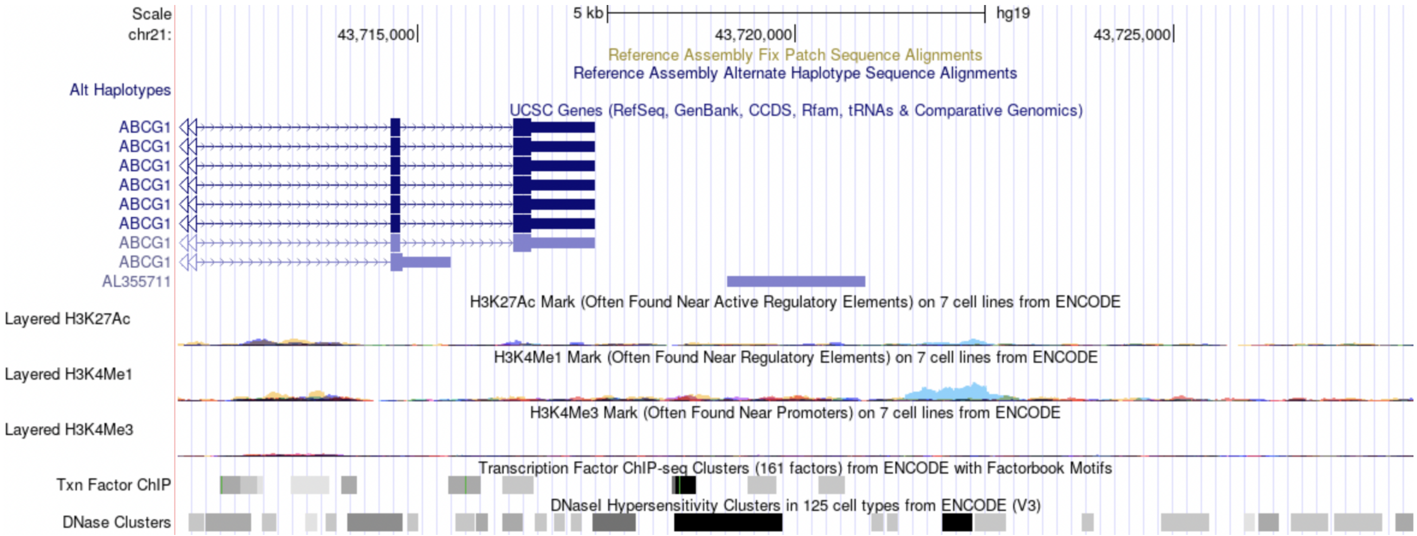

AL355711

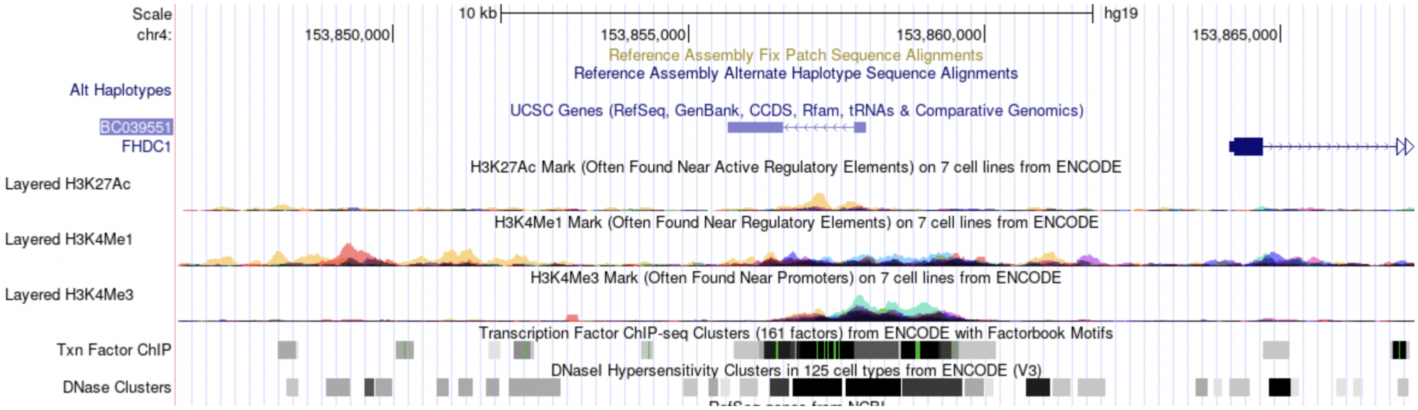

BC039551

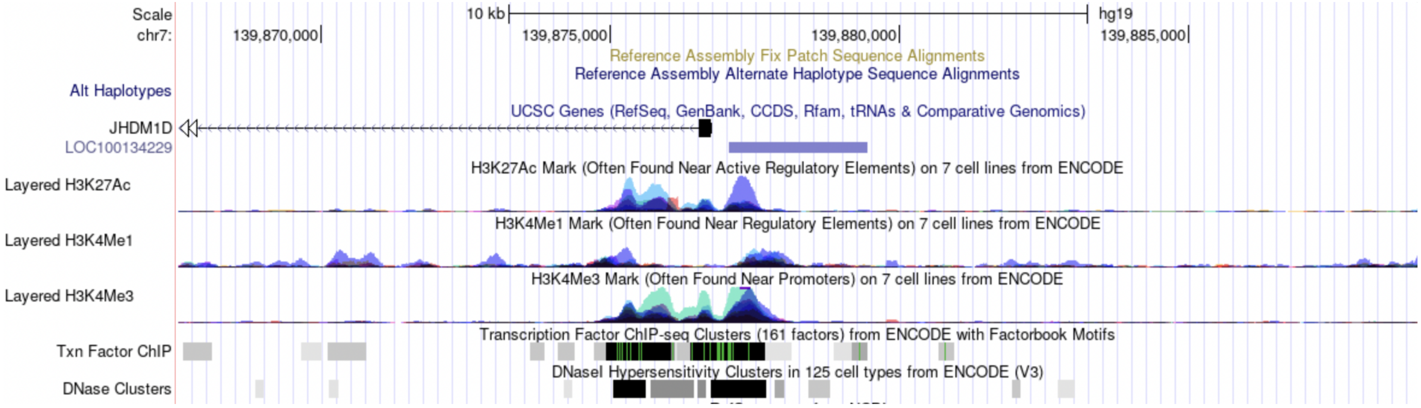

JHDM1D-AS1

Supplementary Figure 30. B. DNAseI Hypersensitivity Sites (display mode, dense), Transcription Factor ChIP Seq (ENCODE) Binding Sites (display mode, dense), and Epigenetic Signatures (display modes, full) of A2M-AS1, NR_034004, and LINC00649.

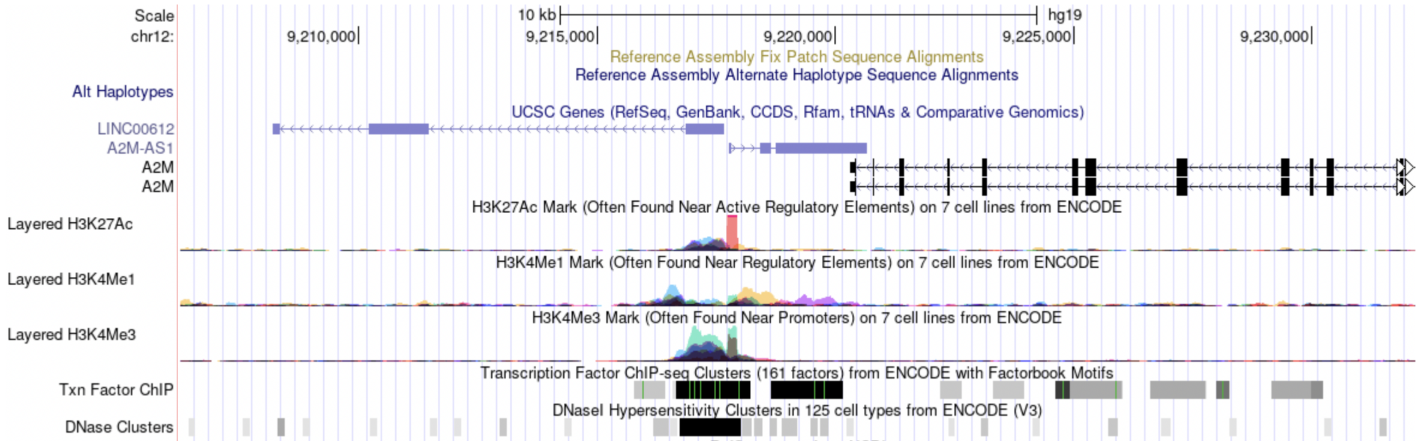

A2M-AS1

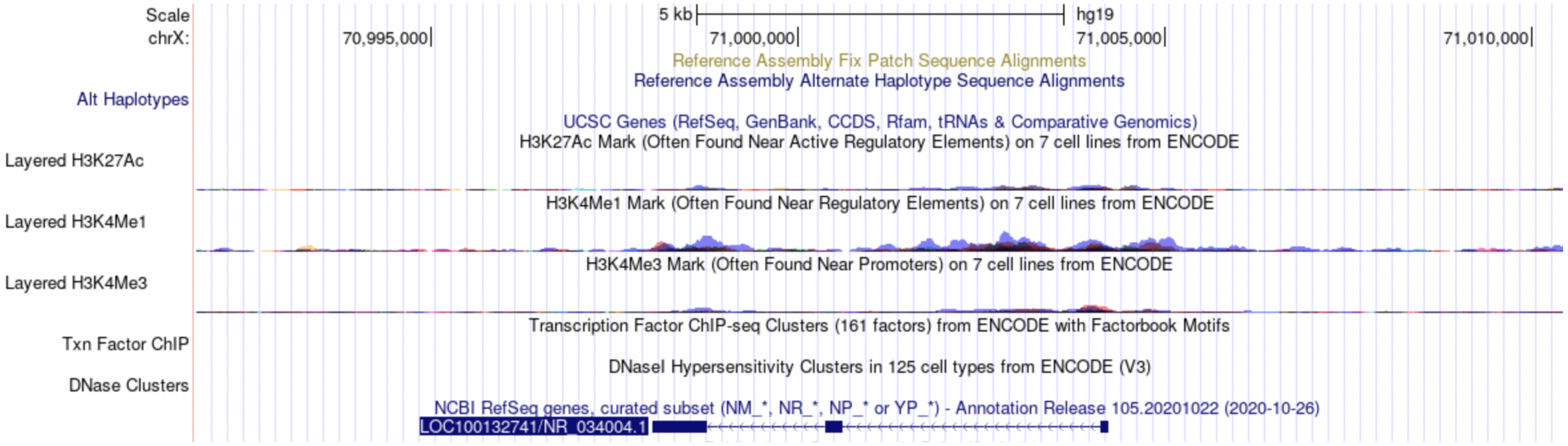

NR_034004

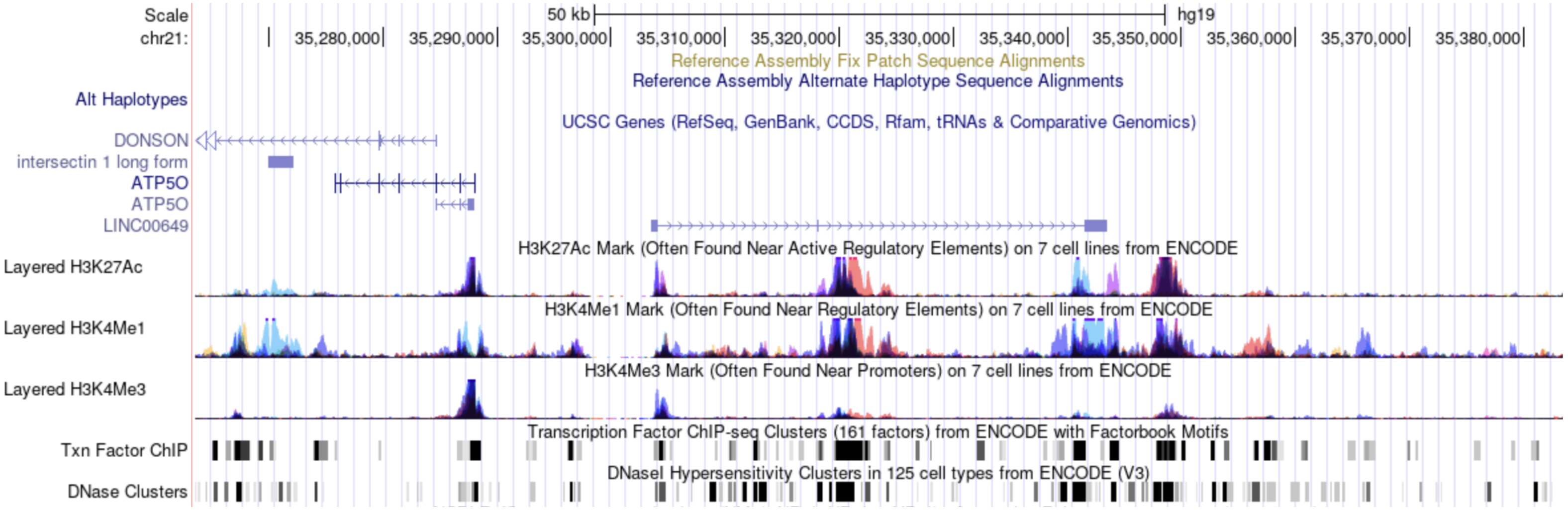

LINC00649

Supplementary Figure 30. C. DNAsel Hypersensitivity Sites (display mode, dense), Transcription Factor ChIP Seq (ENCODE) Binding Sites (display mode, dense), and Epigenetic Signatures (display modes, full) of LINC00861, LINC01094, and MANEA-AS1.

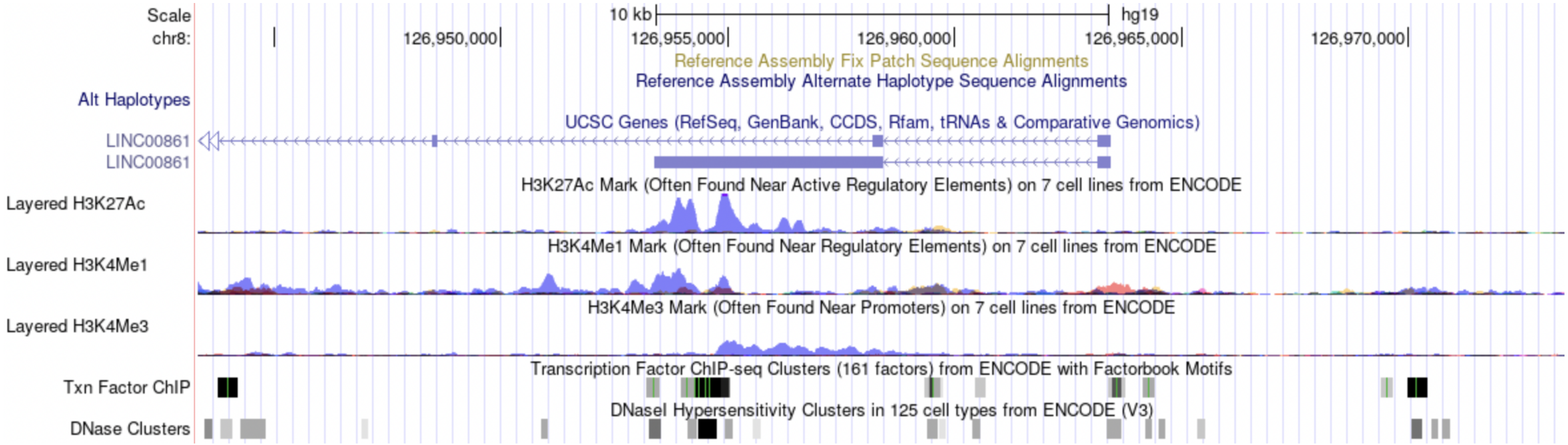

LINC00861

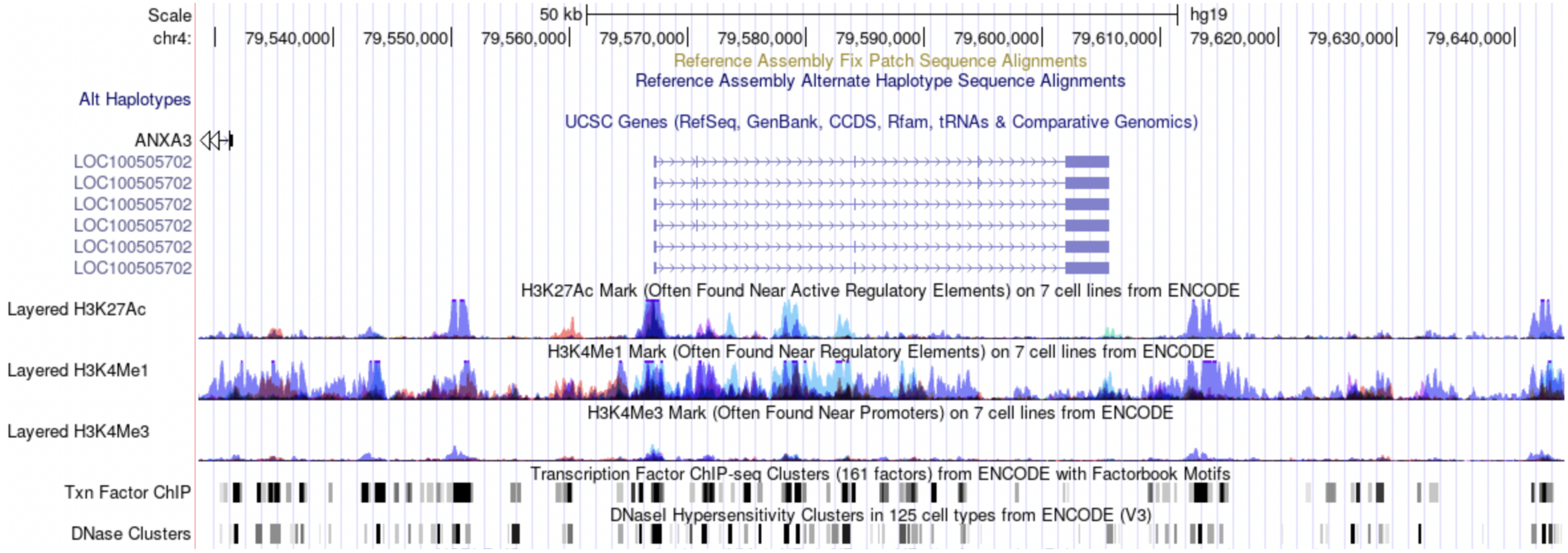

LINC01094

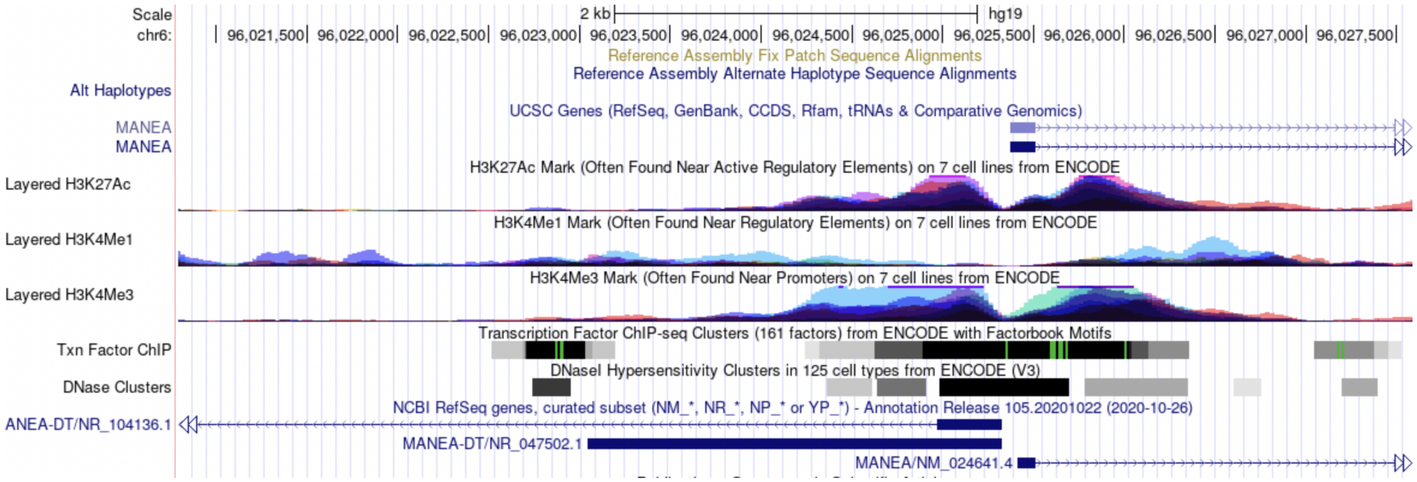

MANEA-AS1

Supplementary Figure 31: UCSC Genome Browser Profiles of TDE transcribed pseudogenes during normal pregnancy.

Supplementary Figure 31. A. DNAseI Hypersensitivity Sites (display mode, dense), Transcription Factor ChIP Seq (ENCODE) Binding Sites (display mode, dense), and Epigenetic Signatures (display modes, full) of UBBP4, FOXO3B, and MKRN9P .

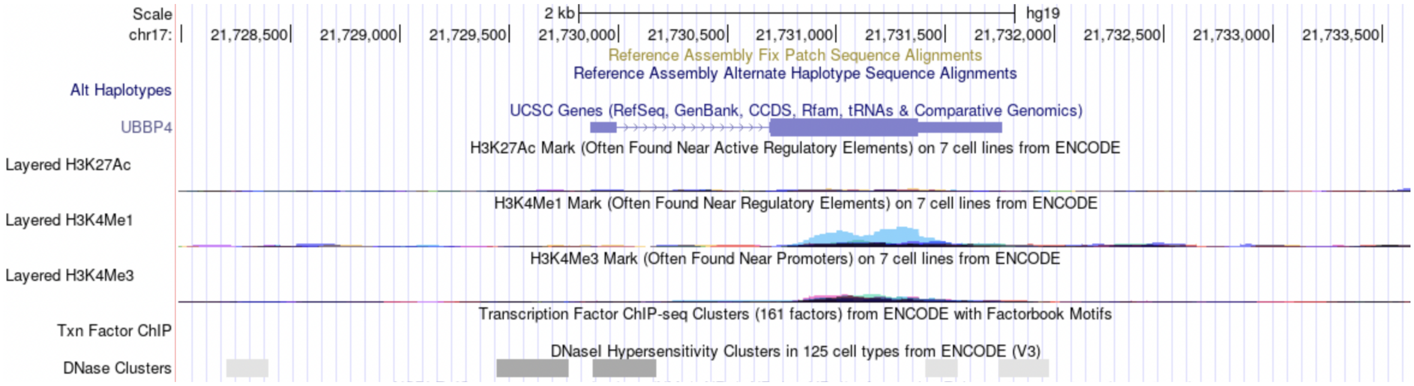

UBBP4

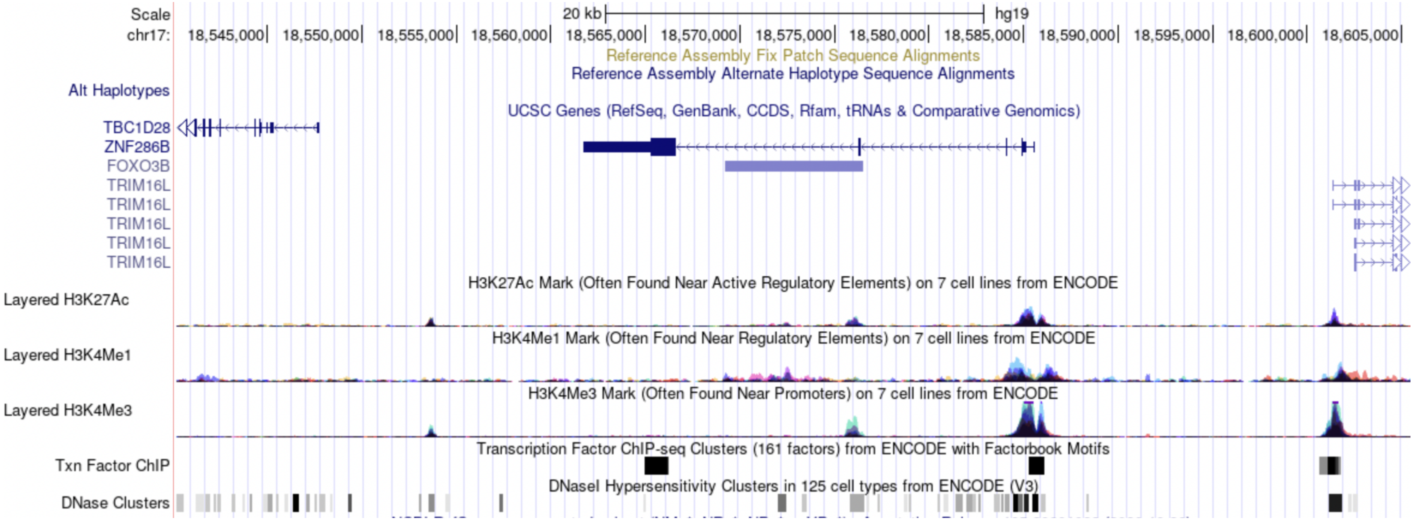

FOXO3B

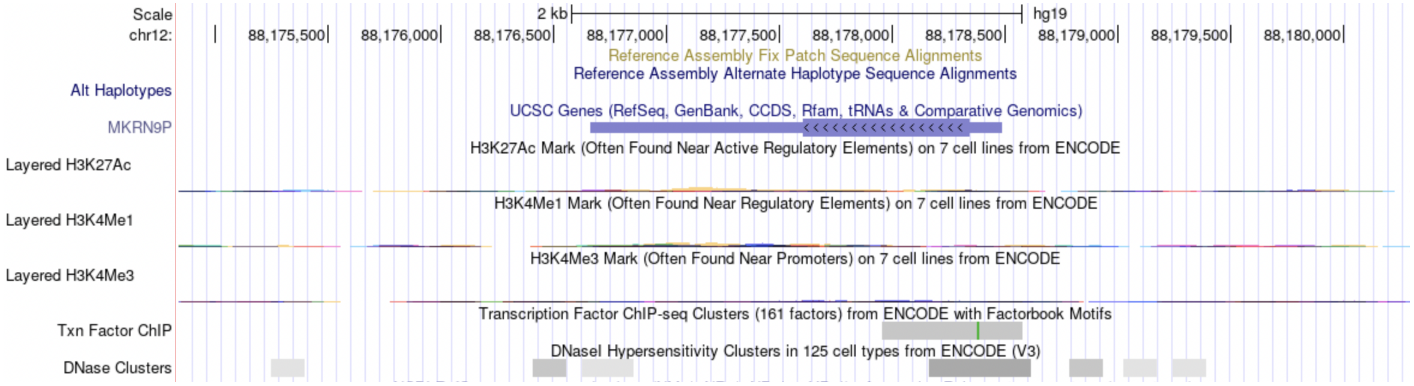

MKRN9P

Supplementary Figure 31. B. DNAseI Hypersensitivity Sites (display mode, dense), Transcription Factor ChIP Seq (ENCODE) Binding Sites (display mode, dense), and Epigenetic Signatures (display modes, full) of LOC441455, PSME2P2 , and YBX3P1 .

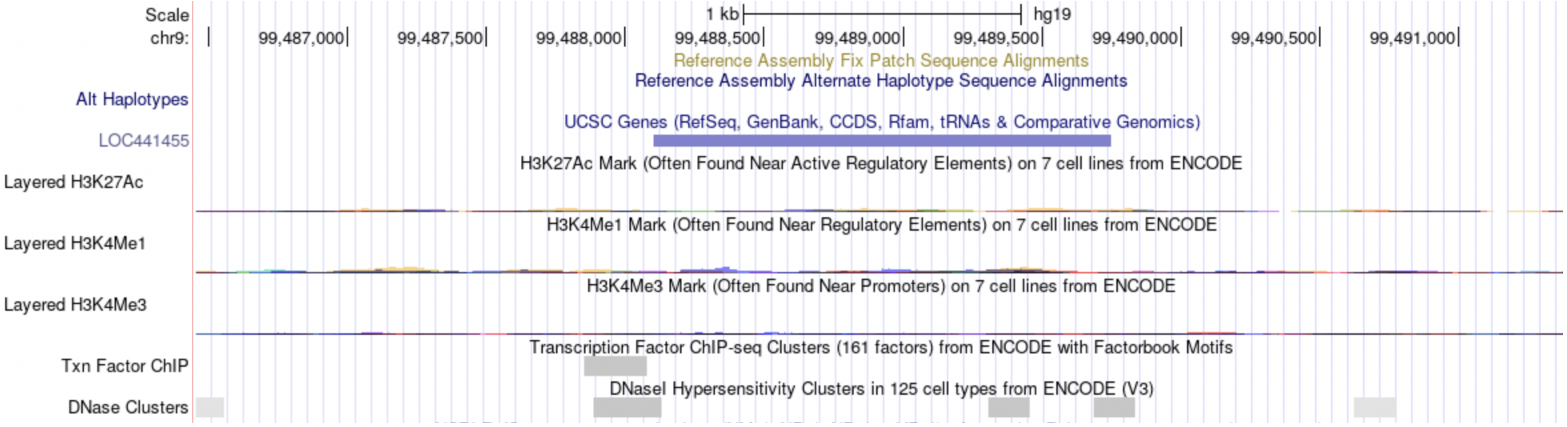

LOC441455

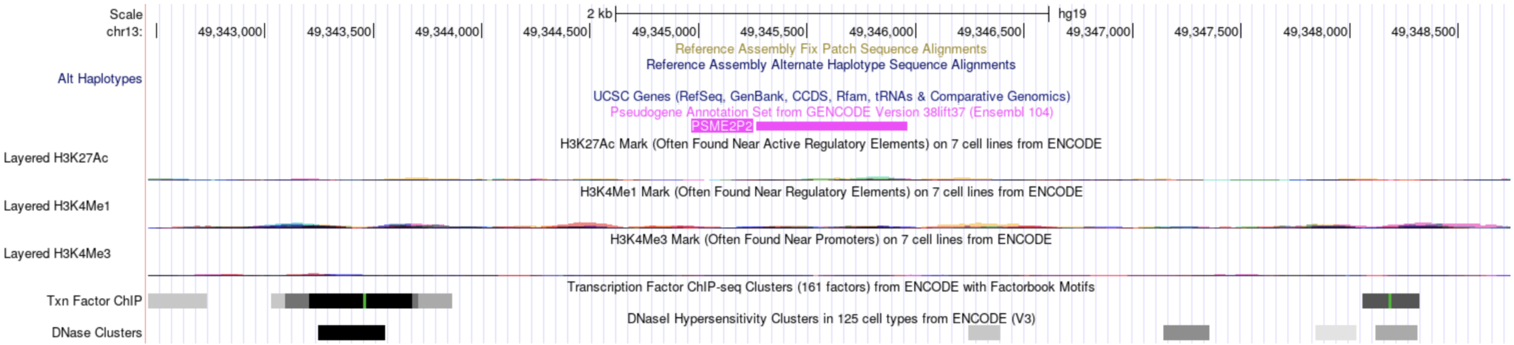

PSME2P2

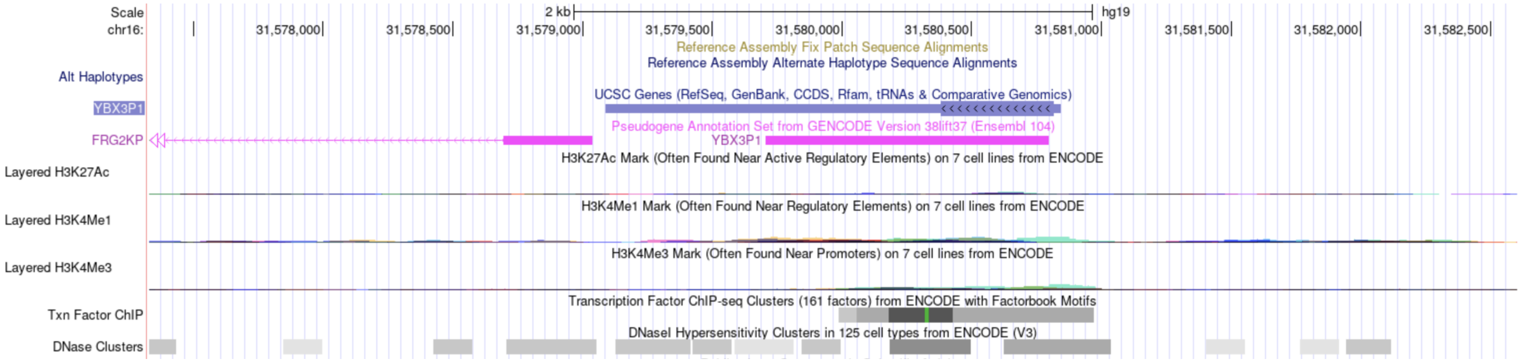

YBX3P1

Supplementary Figure 32: UCSC Genome Browser Profiles of TDE Small and classical non-coding RNAs (ncRNAs) in normal pregnancy.

DNAseI Hypersensitivity Sites (display mode, dense),
Transcription Factor ChIP Seq (ENCODE) Binding Sites (display mode, dense), and Epigenetic Signatures (display modes, full) of MIR4439 , SNORD41 , and SCARNA2 .

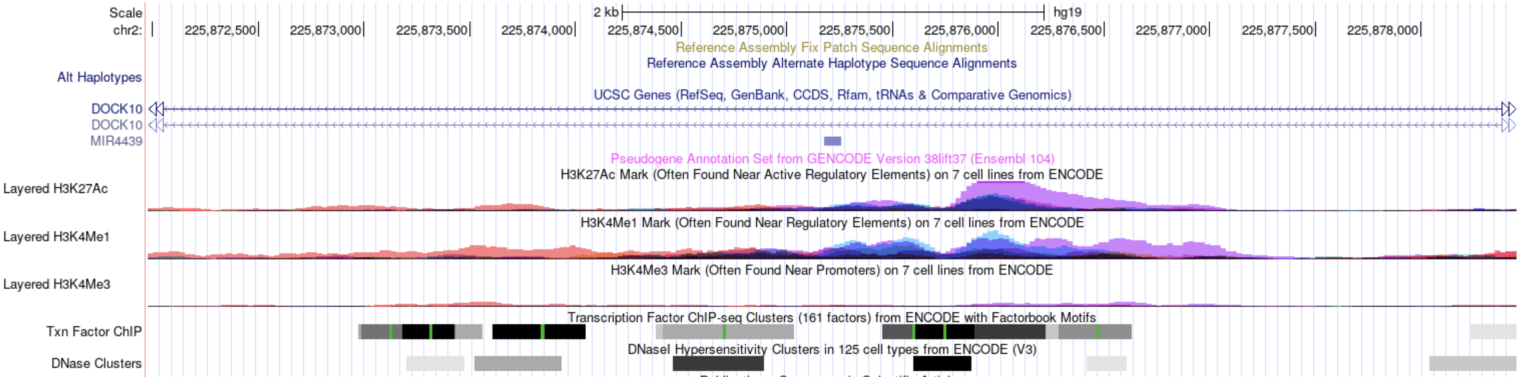

MIR4439

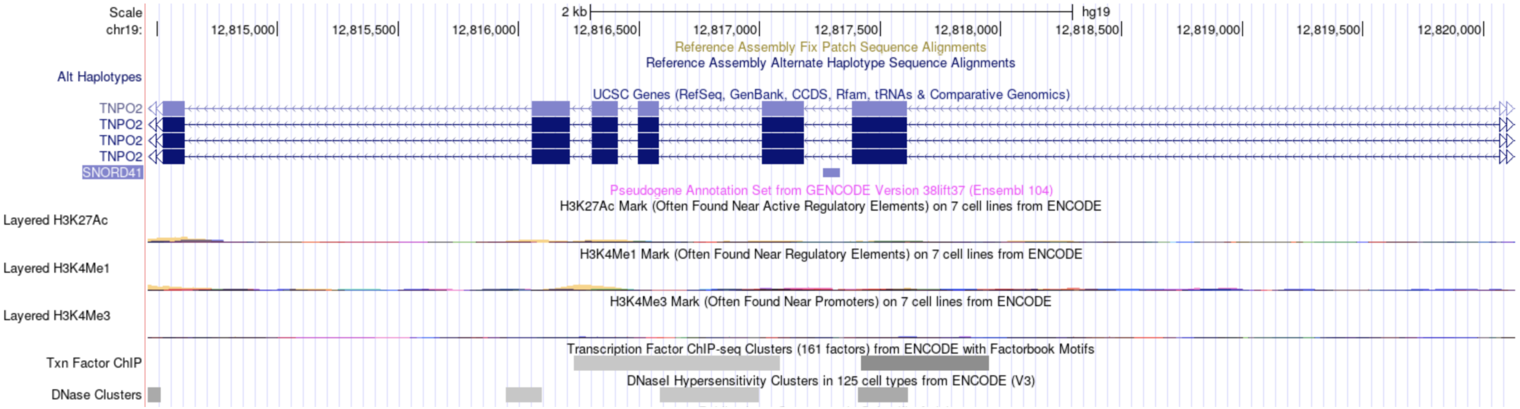

SNORD41

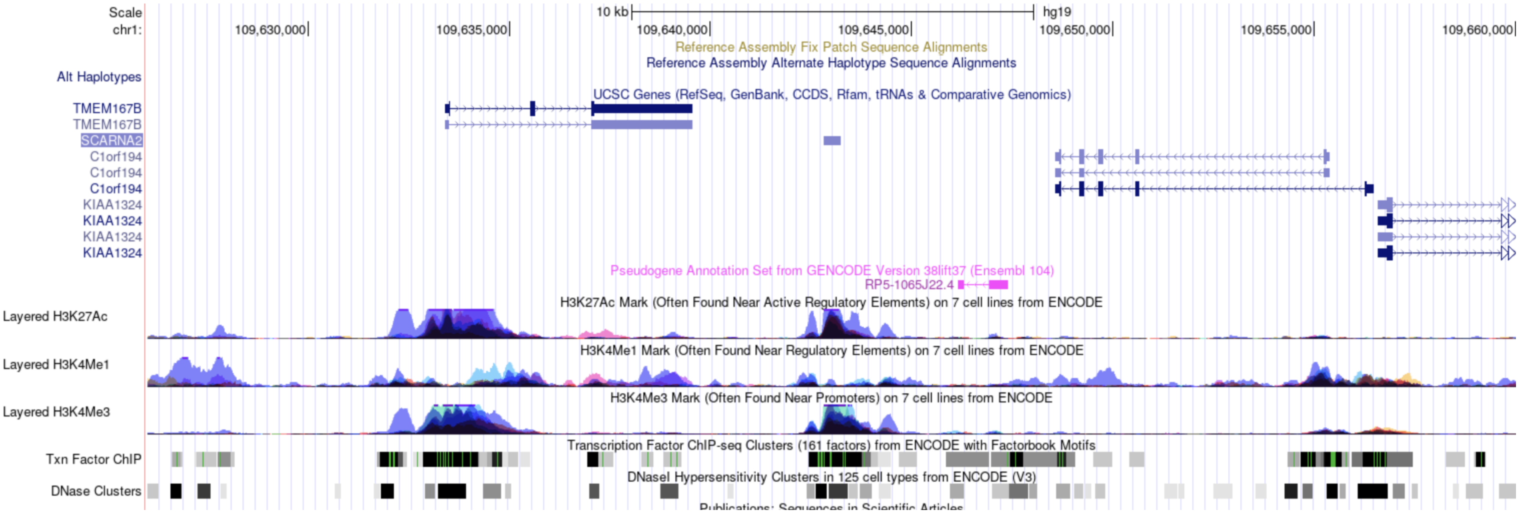

SCARNA2

NONCODING HITLIST

ID	SYMBOL	Name	mRNA	Source	ENTREZ	locus.type	strand	start	stop	Chromosome
TC21000747.hg.1	ABCG1	ATP binding cassette subfamily G member 1	AL355711	NONCODE	9619	NonCoding	+	43719104	43720919	chr21
TC21000200.hg.1	ABCG1	ATP binding cassette subfamily G member 1	AL355711	NONCODE	9619	Coding	+	43719104	43720919	chr21
TC17001226.hg.1	FOXO3B	forkhead box O3B pseudogene	NR_026718	RefSeq	2310	Coding	-	18569236	18576494	chr17
TC17002533.hg.1	FOXO3B	forkhead box O3B pseudogene	BC015367	NONCODE	2310	NonCoding	-	18569236	18576494	chr17
TC07001923.hg.1	MKRN1	makorin ring finger protein 1	NM_001145125	RefSeq	23608	Coding	-	140152840	140179369	chr7
TC12002992.hg.1	MKRN9P	**makorin ring finger protein 9, pseudogene**	NR_033410	NONCODE	400058	NonCoding	-	88176663	88178488	chr12
TC13000192.hg.1	PSME2P2	proteasome activator subunit 2 pseudogene 2	ENST00000419812	ENSEMBL	---	Coding	+	49345759	49345965	chr13
TC09000483.hg.1	LOC441455	**makorin ring finger protein 1 pseudogene**	NR_026792	RefSeq	441455	Coding	+	99488103	99489749	chr9
TC09002124.hg.1	LOC441455	makorin ring finger protein 1 pseudogene	uc011luo.1	NONCODE	441455	NonCoding	+	99488103	99489749	chr9
TC04001652.hg.1	LOC729870	serine/arginine repetitive matrix protein 1	BC039551	NONCODE	729870	Coding	-	153855668	153857989	chr4
TC05001402.hg.1	ISCA1P1	iron-sulfur cluster assembly 1 homolog (S. cerevisiae) p	ENST00000542811	ENSEMBL	---	Coding	-	62072704	62072796	chr5
TC07002622.hg.1	JHDM1D-AS1	JHDM1D antisense RNA 1 (head to head)	AK026372	NONCODE	100134229	NonCoding	+	139877159	139879440	chr7
TC20000923.hg.1	STAU1	staufen double-stranded RNA binding protein 1	NM_001037328	RefSeq	6780	Coding	-	47729876	47805288	chr20
TC07000878.hg.1	JHDM1D-AS1	JHDM1D antisense RNA 1 (head to head)	NR_024451	RefSeq	100134229	Coding	+	139877061	139879440	chr7
TC12001789.hg.1	MKRN9P	makorin ring finger protein 9, pseudogene	NR_033410	RefSeq	400058	Coding	-	88176663	88178488	chr12
TC03000113.hg.1	KAT2B	K(lysine) acetyltransferase 2B	NM_003884	RefSeq	8850	Coding	+	20081515	20195896	chr3
TC16001854.hg.1	SLC6A10P	solute carrier family 6 (neurotransmitter transporter),	NR_003083	NONCODE	386757	NonCoding	-	32888797	32896463	chr16
TC16001066.hg.1	SLC6A10P	solute carrier family 6 (neurotransmitter transporter),	NR_003083	RefSeq	386757	Coding	-	32888797	32896463	chr16
TC04002791.hg.1	LOC729870	serine/arginine repetitive matrix protein 1	BC039551	NONCODE	729870	NonCoding	-	153855668	153857989	chr4
TC16000400.hg.1	SLC6A10PB	solute carrier family 6 (neurotransmitter transporter),	BC068290	GenBankHTC	653562	Coding	+	33778865	33786528	chr16
TC12000139.hg.1	A2M-AS1	A2M antisense RNA 1 (head to head)	NR_026971	RefSeq	144571	Coding	+	9217773	9220651	chr12
TC0X001756.hg.1	LOC100132741	uncharacterized LOC100132741	NR_034004	NONCODE	100132741	NonCoding	+	70917046	70923256	chrX
TC08000911.hg.1	DEFT1P	defensin, theta 1 pseudogene	NR_036686	RefSeq	170949	Coding	-	6844700	6847243	chr8
TC08000913.hg.1	DEFT1P	defensin, theta 1 pseudogene	NR_036686	RefSeq	170949	Coding	-	6863803	6866346	chr8
TC20001622.hg.1	STAU1	staufen double-stranded RNA binding protein 1	BC007532	NONCODE	6780	NonCoding	-	47740922	47804857	chr20
TC21000133.hg.1	LINC00649	long intergenic non-protein coding RNA 649	OTTHUMT00000193777	NONCODE	100506334	Coding	+	35321230	35336260	chr21
TC08002515.hg.1	LINC00861	long intergenic non-protein coding RNA 861	BX648371	NONCODE	100130231	NonCoding	-	126928961	126967898	chr8
TC08001620.hg.1	LINC00861	long intergenic non-protein coding RNA 861	NR_038446	RefSeq	100130231	Coding	-	126934552	126963493	chr8
TC17000299.hg.1	UBBP4	ubiquitin B pseudogene 4	BC070367	GenBankHTC	23666	Coding	+	21729873	21731760	chr17
TC16002075.hg.1	MT1L	metallothionein 1L (gene/pseudogene)	NR_001447	RefSeq	4500	Coding	+	56651373	56652730	chr16
TC16001058.hg.1	YBX3P1	Y box binding protein 3 pseudogene 1	NR_027011	RefSeq	440359	Coding	-	31579088	31580845	chr16
TC21000748.hg.1	ABCG1	ATP binding cassette subfamily G member 1	AF086547	NONCODE	9619	NonCoding	+	43724173	43724497	chr21
TC16001846.hg.1	YBX3P1	Y box binding protein 3 pseudogene 1	NR_027011	NONCODE	440359	NonCoding	-	31579088	31580845	chr16
TC04002081.hg.1	LINC01094	long intergenic non-protein coding RNA 1094	BC044613	NONCODE	100505702	NonCoding	+	79567001	79611023	chr4
TC02000072.hg.1	LINC00570	long intergenic non-protein coding RNA 570	ENST00000417473	ENSEMBL	100874055	Coding	+	11534107	11543203	chr2
TC04000439.hg.1	LINC01094	long intergenic non-protein coding RNA 1094	NR_038303	RefSeq	100505702	Coding	+	79567057	79605655	chr4
TC13000083.hg.1	ATP5EP2	ATP synthase, H+ transporting, mitochondrial F1 comp	NR_002162	RefSeq	432369	Coding	+	28519343	28519727	chr13
TC13000206.hg.1	ST13P4	suppression of tumorigenicity 13 (colon carcinoma) (Hsp	NR_002183	RefSeq	145165	Coding	+	50746154	50747751	chr13
TC19001585.hg.1	CD177P1	CD177 molecule pseudogene 1	ENST00000378007	ENSEMBL	---	Coding	-	43879660	43883276	chr19
TC17001604.hg.1	LRRC37A4P	leucine rich repeat containing 37, member A4, pseudog	uc002ije.3	UCSCGenes	55073	Coding	-	43590734	43593064	chr17
TC07001462.hg.1	SKP1P1	S-phase kinase-associated protein 1 pseudogene 1	ENST00000433793	ENSEMBL	---	Coding	-	65888515	65888655	chr7
TC01000836.hg.1	GBP1P1	guanylate binding protein 1, interferon-inducible pseuc	NR_003133	RefSeq	400759	Coding	+	89873238	89890493	chr1
TC02002828.hg.1	MIR4439	microRNA 4439	NR_039641	RefSeq	100616207	Coding	-	225875178	225875257	chr2
TC01000938.hg.1	SCARNA2	small Cajal body-specific RNA 2	NR_003023	RefSeq	677766	Coding	+	109642815	109643234	chr1
TC19001207.hg.1	SNORD41	small nucleolar RNA, C/D box 41	NR_002751	RefSeq	26810	Coding	-	12817263	12817332	chr19
TC06003771.hg.1	MANEA-AS1	MANEA antisense RNA 1 (head to head)	BC047582	NONCODE	101927288	NonCoding	-	96023059	96025326	chr6
